# The Systems Biology Research Tool: evolvable open-source software

**DOI:** 10.1186/1752-0509-2-55

**Published:** 2008-06-29

**Authors:** Jeremiah Wright, Andreas Wagner

**Affiliations:** 1 Department of Biochemistry, University of Zurich, Zurich, Switzerland; 2 Swiss Institute of Bioinformatics, Lausanne, Switzerland; 3 Sante Fe Institute, Sante Fe, New Mexico, USA

## Abstract

**Background:**

Research in the field of systems biology requires software for a variety of purposes. Software must be used to store, retrieve, analyze, and sometimes even to collect the data obtained from system-level (often high-throughput) experiments. Software must also be used to implement mathematical models and algorithms required for simulation and theoretical predictions on the system-level.

**Results:**

We introduce a free, easy-to-use, open-source, integrated software platform called the *Systems Biology Research Tool *(SBRT) to facilitate the computational aspects of systems biology. The SBRT currently performs 35 methods for analyzing stoichiometric networks and 16 methods from fields such as graph theory, geometry, algebra, and combinatorics. New computational techniques can be added to the SBRT via *process plug-ins*, providing a high degree of evolvability and a unifying framework for software development in systems biology.

**Conclusion:**

The Systems Biology Research Tool represents a technological advance for systems biology. This software can be used to make sophisticated computational techniques accessible to everyone (including those with no programming ability), to facilitate cooperation among researchers, and to expedite progress in the field of systems biology.

## Background

Some of the primary goals of systems biology are to identify and quantify the individual components of cells, organs, and organisms; to understand the interactions between these components; and to use this information to create mathematical models that enable accurate predictions. Since organisms are composed of large numbers of unique elements (i.e. genes, proteins, metabolites, etc.), and since many interactions often exist between these elements, even the most basic forms of system-level data analysis or simulation cannot be done by hand. Instead, software must be used to store, retrieve, analyze, and sometimes even to collect the data obtained from system-level experiments. Software must also be used to implement mathematical models and algorithms required for simulation and theoretical predictions on the system-level.

We introduce an integrated software platform called the *Systems Biology Research Tool *(SBRT) to facilitate the computational aspects of systems biology. The SBRT is useful for both the management and analysis of data, and the simulation and prediction of cellular phenotypes. The SBRT can, for example, be used to translate data files into various machine- and human-readable formats; to simulate the activity of reconstructed signal transduction and genome-scale metabolic networks using *flux balance analysis *and related methods [1,2]; and to analyze the topology of experimentally determined biochemical reaction networks, such as transcriptional regulation and protein-protein interaction networks. Since new data formats, methods of data analysis, and simulation techniques arise frequently during systems biology research, the SBRT is also designed to allow independent software developers to add new functionality as it is needed.

## Implementation

The SBRT is both an application and an application programming interface (API). It is written in Java and has been tested in Windows XP, Mac OS X, and two distributions of Linux, requiring no modification of source code or recompilation. The SBRT is licensed under the GNU General Public License and is therefore open-source, modifiable, and freely distributable. The most recent versions of the SBRT can be downloaded from the SBRT's homepage [3], and an archive of the current version is provided as supplementary material [see Additional file 1].

The Systems Biology Research Tool's API contains over 300 well tested and fully documented classes and interfaces. The API is composed of two functionally distinct levels: the *kernel*, which is responsible for performing all significant computation, and the *shell*, which is responsible for relaying information between the user and the kernel. The kernel is completely independent of the shell, which results in a great degree of flexibility and robustness: new functionality can be added to the kernel without concern for user-level I/O details; new functionality can be added to the shell without modifying the kernel, thereby preventing the introduction of kernel-level errors. The kernel contains implementations of algorithms, methodological procedures, and fundamental *objects*, such as networks, chemical reactions, mathematical expressions, matrices, convex polytopes, hyperplanes, linear program solvers, etc. The shell is primarily composed of classes and interfaces for reading(writing) files from(to) the hard drive, for parsing and formatting various types of data, and for managing and monitoring kernel-level activities.

## Results and discussion

### Use as an application

The SBRT can be used as an application to execute *processes*. A process is a series of actions that takes user-supplied input and produces a result. The SBRT includes 35 processes for analyzing stoichiometric networks, such as optimizing objective functions, computing the variability of fluxes, identifying reaction pathways, generating uniformly distributed points within flux spaces, analyzing the properties of flux vectors and intervals, and more. The SBRT also includes 16 processes utilizing graph theory, geometry, algebra, statistics, and combinatorics. Descriptions of these 51 processes are provided as supplementary material [see Additional file 2].

Processes can be controlled with simple text-based input files (that can be created using common word processing or spreadsheet applications) or directly from the command line. When possible, files generated by one process can also be used as *input *files in other SBRT processes, allowing the user to design complex analyses by linking processes via their input and output files, without writing a single line of code. For example, the process *BiGG-SBML File Reader *can be used to translate a machine-readable file into a human-readable and -editable text file *R *that contains a list of chemical reactions. The file *R *can then be supplied to the *Network Information Gatherer *process to create a text file *N *that contains the names (or IDs) of all chemical reactions contained in *R*; and *R *can also be supplied to the *Random Constraint Generator *process to create a text file *C *of randomly generated flux constraints. The files *R*, *N*, and *C *can then be supplied to the *FBA Constraint Variation-Objective Function Analysis *process to determine the maximum fluxes of the reactions in *R *that are denoted in *N *for each set of flux constraints in *C*. Each of these files can be edited by the user at any step, and many other combinations of processes are possible.

**Figure 1:**
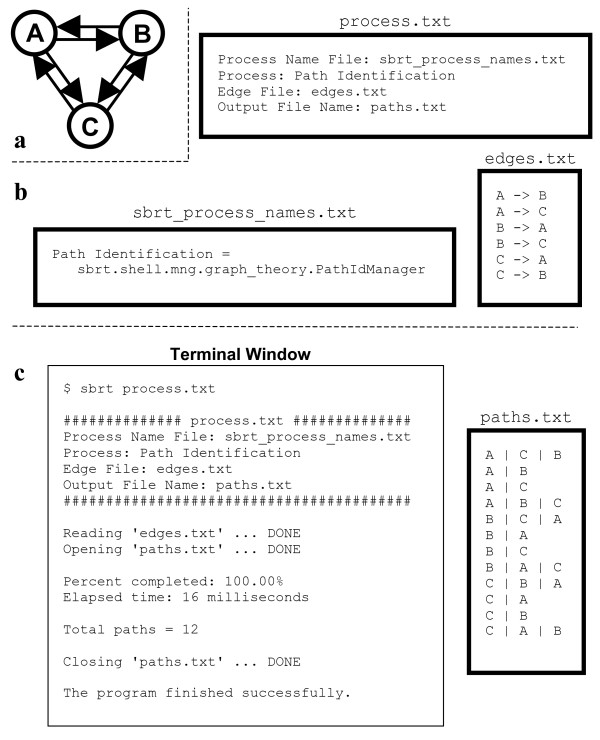
Identifying the simple paths in a directed graph. (**a**) The graph under consideration. (**b**) The input files to the SBRT. (**c**) The execution of the SBRT from the command line and its subsequent output. Rectangles with thick borders represent text files, with their name denoted directly above. The file edges.txt is created by the user to store the edges of the graph in **a**. The file sbrt_process_names.txt is used to define a name for the process and also provides part of the mechanism for incorporating process plug-ins. The file process.txt is used to organize the input, and all simple paths in the graph are identified with the command **sbrt process.txt**. The file paths.txt is created by the SBRT with a single path on each line, with nodes delimited by the pipe character.

The use of the SBRT as an application requires no programming ability, and is fully documented in a freely available HTML-based *User's Guide*, which provides a detailed description of each process and contains hyperlinks to at least one complete example. An example of the *Path Identification *process is illustrated in Figure 1.

### Support for external software

The Systems Biology Research Tool's API is designed to support multiple forms of *external *software (software not included in the SBRT's API), making the SBRT highly modular and thus evolvable. A *process plug-in *is an external software package that can be written by any skilled programmer, executed as a process by the SBRT application, and shared among other users. As a consequence of the existing capabilities of the SBRT, development of process plug-ins is considerably easier and faster than development of new stand-alone applications. Plug-ins can, for example, call high-level methods from the API that perform file parsing, process monitoring, algorithm execution, and error-detection. Plug-ins can also call low-level methods to facilitate the development of novel high-level methods. Instructions for writing process plug-ins are included in the *Developer's Guide*, and an example plug-in is also included with the package. Additionally, the SBRT's API supports communication with other forms of external software, such as applications and software libraries. The ability to interact with Mathematica, R, GLPK, CPLEX, Xerces, and Metatool [4,5] is already implemented.

### Similar software

Due to its ability to communicate with other software, the Systems Biology Research Tool provides some functionality similar to that of Cytoscape [6], CellDesigner [7], and the Systems Biology Workbench [8]. Both Cytoscape and CellDesigner can also be extended via plug-ins, but their current capabilities are substantially different from those of the SBRT. The Systems Biology Workbench is primarily intended to unify other applications by acting as a broker. The SBRT can be used in a similar way, but this is not its primary function. The SBRT can be used independently of other applications, and it also provides implementations of algorithms not currently available in any other software package [9].

Presently, the majority of processes offered by the Systems Biology Research Tool are for analyzing stoichiometric networks. Software already exists that is capable of particular types of such analysis, such as the COBRA Toolbox [10], CellNetAnalyzer [11], Metatool [4,5], FBA3, moma [12], PathwayAnalyser [13], expa [14], YANA [15], and SNA [16]. Some of these programs are stand-alone applications (Metatool 4.x, FBA3, moma, PathwayAnalyser, expa, YANA), and the remainder can only function within a specific programming environment, such as MATLAB or Mathematica (Metatool 5.0, COBRA Toolbox, CellNetAnalyzer, SNA). In Table 1 and the following section, we compare and contrast some of the features and designs of these programs with that of the Systems Biology Research Tool.

**Table 1:** Features of the Systems Biology Research Tool and similar software packages

Software Package	Systems Biology Research Tool	COBRA Toolbox 1.3.3	CellNetAnalyzer 9.0	Metatool 4.9.2	Metatool 5.0	PathwayAnalyser 1.0	expa	YANA 0.9.8	SNA
Provides a graphical installation procedure	✓								
Requires separate installation of other software packages for basic functionality		✓	✓		✓	✓		✓	✓
Requires commercial software for basic functionality		✓	✓						✓
Windows compatible	✓	✓	✓	✓	✓		✓	✓	
Mac compatible	✓	✓	✓		✓		✓	Unknown	
Linux compatible	✓	✓	✓	✓	✓	✓	✓	✓	✓
Requires programming ability to use		✓			✓				✓
Can be used via a command line interface	✓	✓	✓	✓	✓	✓	✓		✓
Provides a graphical user interface	✓		✓					✓	
Provides a documented API	✓	✓	✓		In Progress				

### Evolvability

Due to its API and support for external software, the SBRT has the ability to evolve in conjunction with the field of systems biology itself. In contrast, none of the stand-alone applications for stoichiometric network analysis listed above (Metatool 4.x, FBA3, moma, PathwayAnalyser, expa, YANA) provide both a documented API and a mechanism for the inclusion of additional software (other than by modifying existing source code). Therefore, the ability of independent software developers to expand upon these programs is greatly hindered. This is not the case, however, for software written for MATLAB or Mathematica. These mathematical programming environments both provide a large number of powerful functions, well documented API's, and mechanisms for the inclusion of external software, making the development of new software straightforward. MATLAB and Mathematica, however, are both closed-source. Consequently, certain aspects of their performance and functionality are impossible to alter, which results in additional constraints during software development and limitations during performance optimization.

### Cost

To our knowledge, all of the stoichiometric network analysis software listed above is free of charge, at least for academic purposes. MATLAB and Mathematica, however, are both commercial software packages. In contrast, the SBRT is completely free of charge for every user.

### Ease of use

One of the most important aspects of any software package is its ease of installation and use. The SBRT differs from the programs listed above in several ways. First, some of these programs require the installation of libraries or other programs before they can be used, while SBRT installation is self-contained and guided with a graphical user interface. Second, some of the existing programs must be used from a command line interface, which is cumbersome for the "typical" Windows user. The SBRT can be used from both the command line and from a simple graphical user interface. Third, while some existing programs require programming ability, the SBRT does not, when used as an application.

### Scope

The programs listed above are intended primarily for different types of stoichiometric network analyses, and they are sometimes quite limited in scope. The SBRT, however, has been explicitly designed to integrate techniques from all of systems biology.

### Performance

**Figure 2:**
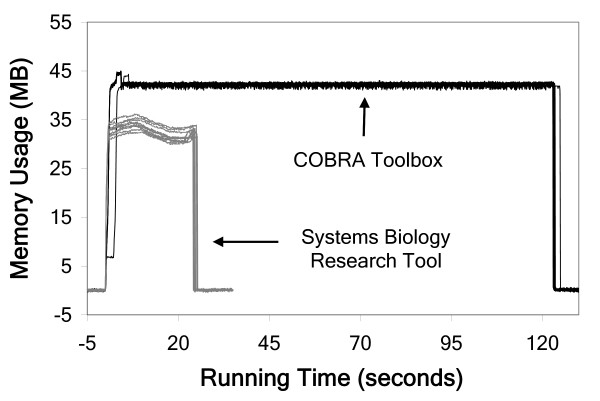
Memory usage vs. running time for the SBRT (grey) and COBRA Toolbox (black) for 10 executions each of analysis *A*.

Of all existing packages, the COBRA Toolbox is most similar to the SBRT in terms of the computational procedures offered by both. Because of these similarities, we performed a comparative performance analysis of some capabilities offered by both packages. Specifically, we carried out 5 analyses using an *in silico *model of *S. cerevisiae *metabolism [17]. For analyses *A *and *B*, the model was provided a minimal growth-supporting medium, where the variability of all reaction rates (*A*) and the effect of all single-gene deletions on the maximum growth rate (*B*) were computed. For analyses *C*, *D*, and *E*, the model was sequentially provided 100 randomly generated growth-supporting media, where the maximum growth rate (*C*), the variability of all reaction rates (*D*), and the effect of all single-gene deletions (*E*) were computed. The average maximum memory usage of the COBRA Toolbox was 1.30 (*A*), 1.00 (*B*), 1.01 (*C*), 0.96 (*D*), and 0.65 (*E*) times that of the SBRT; and the SBRT was 5.00 (*A*), 2.75 (*B*), 1.06 (*C*), 4.87 (*D*), and 3.73 (*E*) times faster than the COBRA Toolbox (Figure 2). A detailed description of these comparisons is provided as supplementary material [see Additional file 3].

## Conclusion

The Systems Biology Research Tool represents a technological advance for systems biology. This software can be used to make sophisticated computational techniques available to everyone, to facilitate cooperation among researchers, and to expedite progress in the field of systems biology.

## Availability and requirements

Project name: The Systems Biology Research Tool

Project home page: 

Operating system(s): Windows, Mac OS X, Linux, Platform independent

Programming Language: Java

Other requirements: None

License: GNU General Public License

Any restrictions to use by non-academics: None

## Authors' contributions

JW designed and implemented the Systems Biology Research Tool and carried out all performance comparisons. Both JW and AW contributed to the software's conception and participated in drafting the manuscript.

## Supplementary Material

Additional file 1 SBRT Archive. An archive of the current version of the Systems Biology Research Tool. Click here for file

Additional file 2 SBRT Processes. Descriptions of the 51 processes currently implemented in the Systems Biology Research Tool. Click here for file

Additional file 3 Performance Comparisons. A description of performance comparisons between the Systems Biology Research Tool and the COBRA Toolbox. Click here for file

## References

[B1] Price ND, Papin JA, Schilling CH, Palsson BO (2003). Genome-scale microbial in silico models: the constraints-based approach. Trends in Biotechnology.

[B2] Price ND, Reed JL, Palsson BO (2004). Genome-scale models of microbial cells: evaluating the consequences of constraints. Nature Reviews Microbiology.

[B3] The Systems Biology Research Tool's Homepage. http://www.bioc.uzh.ch/wagner/software/SBRT.

[B4] Kamp A, Schuster S (2006). Metatool 5.0: fast and flexible elementary modes analysis. Bioinformatics.

[B5] Pfeiffer T, Sanchez-Valdenebro I, Nuno JC, Montero F, Schuster S (1999). METATOOL: for studying metabolic networks. Bioinformatics.

[B6] Shannon P, Markiel A, Ozier O, Baliga NS, Wang JT, Ramage D, Amin N, Schwikowski B, Ideker T (2003). Cytoscape: A Software Environment for Integrated Models of Biomolecular Interaction Networks. Genome Research.

[B7] Funahashi A, Morohashi M, Kitano H, Tanimura N (2003). CellDesigner: a process diagram editor for gene-regulatory and biochemical networks. Biosilico.

[B8] Sauro HM, Hucka M, Finney A, Wellock C, Bolouri H, Doyle J, Kitano H (2003). Next Generation Simulation Tools: The Systems Biology Workbench and BioSPICE Integration. Omics A Journal of Integrative Biology.

[B9] Wright J, Wagner A (2008). Exhaustive identification of steady state cycles in large stoichiometric networks. (submitted).

[B10] Becker SA, Feist AM, Mo ML, Hannum G, Palsson BO, Herrgard MJ (2007). Quantitative prediction of cellular metabolism with constraint-based models: the COBRA Toolbox. Nat Protocols.

[B11] Klamt S, Saez-Rodriguez J, Gilles ED (2007). Structural and functional analysis of cellular networks with CellNetAnalyzer. BMC Systems Biology.

[B12] Segre D, Vitkup D, Church GM (2002). Analysis of optimality in natural and perturbed metabolic networks. PNAS.

[B13] Raman K, Chandra N (2008). PathwayAnalyser: A systems biology tool for flux analysis of metabolic pathways. Nature Precedings.

[B14] Bell SL, Palsson BO (2005). expa: a program for calculating extreme pathways in biochemical reaction networks. Bioinformatics.

[B15] Schwarz R, Musch P, von Kamp A, Engels B, Schirmer H, Schuster S, Dandekar T (2005). YANA-a software tool for analyzing flux modes, gene-expression and enzyme activities. BMC Bioinformatics.

[B16] Urbanczik R (2006). SNA–a toolbox for the stoichiometric analysis of metabolic networks. BMC Bioinformatics.

[B17] Duarte NC, Herrgard MJ, Palsson BO (2004). Reconstruction and Validation of Saccharomyces cerevisiae iND750, a Fully Compartmentalized Genome-Scale Metabolic Model. Genome Res.

