# Supplementary material for: The Systems Biology Research Tool: evolvable open-source software
Source: BMC Syst Biol. 2008 Jun 29;2:55. doi: 10.1186/1752-0509-2-55 (PMC2446383; doi:10.1186/1752-0509-2-55)
Supplement: Additional file 1 — SBRT Archive. An archive of the current version of the Systems Biology Research Tool. [file 1752-0509-2-55-S1.zip › sbrt-1.4.0/doc/users_guide/fba/definitions/flux_space.html]

Flux Space - Systems Biology Research Tool


|  |
| --- |
| > User's Guide > Flux Balance Analysis |
|  |
| Flux Space |
|  |
| Definition Let *S* denote an *m x n* stoichiometry matrix, and let *v* denote a vector of *n* fluxes. The term **flux space** refers to the set of flux vectors that satisfy all of the constraints defined for the system *Sv = 0*. The Null Space The set of solutions to the equation *Sv = 0* is referred to as the *null space*, or *kernel*, of *S*. The dimensions of the null space are *n - r*, where *r* is the rank of *S*. By specifying values for the *n - r* free variables, all *n* fluxes in *v* are uniquely determined. Flux Constraints Fluxes are always constained to lie on intervals. These intervals may be bounded in both directions, such as [0, 5], or they may be unbounded, such as [0, ∞), (-∞, 0], or (-∞, ∞). If each flux in *v* is constrained to lie on a bounded interval, an *n* dimensional hyperbox is formed. (A hyperbox is a particular type of convex polytope.) If these flux constraints in *n* dimensional space are mapped to the *n - r* dimensional null space, a convex polytope *P* is still formed, and this polytope is not necessarily a hyperbox. Intersections Let *F* denote the intersection of the null space of *S* and the convex polytope *P*. Flux space is obtained by mapping each point in *F* into a vector *v* of *n* fluxes. |

  
