# Supplementary material for: The Systems Biology Research Tool: evolvable open-source software
Source: BMC Syst Biol. 2008 Jun 29;2:55. doi: 10.1186/1752-0509-2-55 (PMC2446383; doi:10.1186/1752-0509-2-55)
Supplement: Additional file 1 — SBRT Archive. An archive of the current version of the Systems Biology Research Tool. [file 1752-0509-2-55-S1.zip › sbrt-1.4.0/doc/users_guide/fba/definitions/stoich_equiv.html]

Stoichiometric Equivalence - Systems Biology Research
Tool


|  |
| --- |
| > User's Guide > Flux Balance Analysis |
|  |
| Stoichiometric Equivalence |
|  |
| For two reactions, *A* and *B*, to be considered stoichiometrically equivalent, the following must be true:  - The set of reactants for A and B must be identical. - The set of products for A and B must be identical. - The stoichiometric coefficients of A must be proportional to   the corresponding coefficients in B.   For example, the reactions   |  |  |  |  |  | | --- | --- | --- | --- | --- | | 2 H2 + O2 → 2 H2O |  | and |  | H2 + ½ O2 → H2O |   are stoichiometrically equivalent. |

  
