# Supplementary material for: The Systems Biology Research Tool: evolvable open-source software
Source: BMC Syst Biol. 2008 Jun 29;2:55. doi: 10.1186/1752-0509-2-55 (PMC2446383; doi:10.1186/1752-0509-2-55)
Supplement: Additional file 1 — SBRT Archive. An archive of the current version of the Systems Biology Research Tool. [file 1752-0509-2-55-S1.zip › sbrt-1.4.0/doc/users_guide/fba/processes/flux_variability/Flux_Cap_Id.html]

Flux Cap Identification - Systems Biology Research Tool


|  |
| --- |
| > User's Guide > Flux Balance Analysis > Flux Variability |
|  |
| Flux Cap Identification  This process is used to identify all of the flux caps in a stoichiometric network. This process requires the set of all steady state cycles (type III extreme pathways) within the provided reaction network, which can be generated using one of the cycle identification processes. The output of a cycle identification process can be used directly as input to this process.  Here is the set of keywords this process understands, along with a description of their possible corresponding values. |

  


|  |  |
| --- | --- |
| Required Keywords | Possible Values |
| Process Name File | The name of the file where process names are defined. See  Process Name Files for further information. |
| Process | The name defined in the specified process name file.  FBA Flux Cap Identification is the default value. |
| Reaction File | The name of a text file containing the internal reactions of a stoichiometric network. See FBA Reaction Files for further information. |
| Cycle File | The name of a file containing all cycles in the specified stoichiometric network. See Multiple-Flux Vectors Files for further information. |
| Output File Name | The name of the file to which the flux caps will be written. See Flux Cap Files for further information. |

|  |
| --- |
|  |

|  |
| --- |
| Examples Click here for an example. |
