# Supplementary material for: The Systems Biology Research Tool: evolvable open-source software
Source: BMC Syst Biol. 2008 Jun 29;2:55. doi: 10.1186/1752-0509-2-55 (PMC2446383; doi:10.1186/1752-0509-2-55)
Supplement: Additional file 1 — SBRT Archive. An archive of the current version of the Systems Biology Research Tool. [file 1752-0509-2-55-S1.zip › sbrt-1.4.0/doc/users_guide/fba/processes/flux_variability/CV_Simple_Flux_Intervals.html]

FBA Constraint Variation-Simple Flux Intervals - Systems
Biology Research Tool


|  |
| --- |
| > User's Guide > Flux Balance Analysis > Flux Variability |
|  |
| FBA Constraint Variation-Simple Flux Intervals This process is used to compute the *simple* intervals of fluxes for multiple sets of flux constraints. These intervals are computed by minimizing and maximizing the flux of each reaction using a linear program solver. If the minimization or maximization problems prove to be unbounded, the flux bounds are denoted as -∞ or +∞, respectively. The following steps are executed during this process:   |  |  | | --- | --- | | 1. | If a *constraints file* is specified, the flux constraints it contains are applied. | | 2. | The next set of flux constraints contained in the *constraint variation file* are applied. These constraints will override any pre-existing constraints for the specified fluxes (or linear combinations of fluxes). | | 3. | The flux intervals are computed, and the results are written to the next line of the specified output file. | | 4. | If another set of flux constraints is present in the *constraint variation file*, Steps 2 and 3 are repeated. |   Here is the set of keywords this process understands, along with a description of their possible corresponding values. See the command line documentation for more information about keyword-value pairs. |

  


|  |  |
| --- | --- |
| Required Keywords | Possible Values |
| Process Name File | The name of the file where process names are defined. See  Process Name Files for further information. |
| Process | The name defined in the specified process name file.  FBA Constraint Variation-Simple Flux Intervals is the default value. |
| Reaction File | The name of a text file containing the internal reactions of a stoichiometric network. See FBA Reaction Files for further information. |
| Constraint Variation File | The name of a text file containing the user-defined flux constraint variations. See Constraint Variation Files for further information. |
| Program Solver | The name of the program solver to be used to compute the flux intervals. See Program Solvers for further information. |
| Output File Name | The name of the file to which the computed flux intervals will be written. See Single-Flux Interval Vector Files for further information. |
|  |
| Optional Keywords | Possible Values |
| Constraints File | The name of a text file containing an initial set of flux constraints. See Constraints Files for further information. |
| Data Headers | The data headers of the specified output file. See Reaction Name Data Headers for further information. |
| Zero Cutoff | The amount by which a computed lower bound can *exceed* a computed upper bound. See Zero Cutoffs for further information. |
| Constraint Tolerance | The amount by which the linear program solver is allowed to violate the defined flux constraints. See Constraint Tolerances for further information. |
| Safety Level | The saftey level at which the optimizations will be performed. See Safety Levels for further information. |
| Program Solver Parameter File | The name of the file containing parameters for the linear program solver. See Program Solver Parameter Files for further information. |

|  |
| --- |
|  |

|  |
| --- |
| Examples Click here for an example. |
