# Supplementary material for: The Systems Biology Research Tool: evolvable open-source software
Source: BMC Syst Biol. 2008 Jun 29;2:55. doi: 10.1186/1752-0509-2-55 (PMC2446383; doi:10.1186/1752-0509-2-55)
Supplement: Additional file 1 — SBRT Archive. An archive of the current version of the Systems Biology Research Tool. [file 1752-0509-2-55-S1.zip › sbrt-1.4.0/doc/users_guide/fba/processes/flux_variability/index.html]

FBA Flux Variability - Systems Biology Research Tool


|  |
| --- |
| > User's Guide > Flux Balance Analysis |
|  |
| Flux Variability |
|  |
| Fluxes are constrained to lie on a particular interval, such as [0, ∞). This does not mean, however, that a flux can necessarily assume all values along that interval. The processes in this section are used to determine the minimum and maximum values fluxes can actually achieve, that is, their true intervals. |

  
  


|  |  |
| --- | --- |
| Processes | Brief Descriptions |
| Simple Flux Intervals | Used to compute the intervals of fluxes in a stoichiometric network in the simplest possible way. |
| Constrained Reverse Reaction Flux Intervals | Used to compute the intervals of fluxes in a stoichiometric network after constraining the fluxes of reversible reactions. |
| Flux Cap Identification | Used to create *caps* for each unbounded flux in a stoichiometric network. |
| Mahadevan-Schilling Flux Intervals | Used to compute the Mahadevan-Schilling flux intervals in a stoichiometric network. |
| Constraint Variation-Simple Flux Intervals | Used to compute the *simple* flux intervals for multiple sets of flux constraints. |
| Constraint Variation-Constrained Reverse Reaction Flux Intervals | Used to compute *constrained reverse reaction* flux intervals for multiple sets of flux constraints. |
| Constraint Variation-Mahadevan-Schilling Flux Intervals | Used to compute Mahadevan-Schilling flux intervals for multiple sets of flux constraints. |
|  |
| Algorithms | Brief Descriptions |
| Flux Caps | A description of flux caps and their use in computing Mahadevan-Schilling flux intervals. |
