# Supplementary material for: The Systems Biology Research Tool: evolvable open-source software
Source: BMC Syst Biol. 2008 Jun 29;2:55. doi: 10.1186/1752-0509-2-55 (PMC2446383; doi:10.1186/1752-0509-2-55)
Supplement: Additional file 1 — SBRT Archive. An archive of the current version of the Systems Biology Research Tool. [file 1752-0509-2-55-S1.zip › sbrt-1.4.0/doc/users_guide/fba/processes/flux_variability/Constrained_Reverse_Rxn_Flux_Intervals.html]

Constrained Reverse Reaction Flux Intervals - Systems
Biology Research Tool


|  |
| --- |
| > User's Guide > Flux Balance Analysis > Flux Variability |
|  |
| Constrained Reverse Reaction Flux Intervals This process is used to compute the intervals of fluxes in a stoichiometric network after the fluxes of reversible reactions have been constrained, or *capped*. An upper bound is first determined for each flux in the stoichiometric network by computing the maximum flux of each reaction *r* in the following way:   |  |  | | --- | --- | | 1. | A set *R* is created that contains the reactions in the stoichiometric network that are the reverse of *r*. | | 2. | The minimum value of Σ *fi* is computed and denoted as α, where *fi* denotes the flux of reaction *ri* in the set *R*. | | 3. | Σ *fi* is contrained to the interval [α, α]. | | 4. | The maximum flux of *r* is computed and saved for later use. | | 5. | The constraint on Σ *fi* is restored to its original value. |   The values computed in Step 4 are then used to define the upper bound on their respective fluxes. The intervals of each flux are then computed by minimizing and maximizing the flux of each reaction. If the minimization or maximization problems prove to be unbounded, the flux bounds are denoted as -∞ or +∞, respectively.   Here is the set of keywords this process understands, along with a description of their possible corresponding values. See the command line documentation for more information about keyword-value pairs. |

  


|  |  |
| --- | --- |
| Required Keywords | Possible Values |
| Process Name File | The name of the file where process names are defined. See  Process Name Files for further information. |
| Process | The name defined in the specified process name file.  FBA Constrained Reverse Reaction Flux Intervals is the default value. |
| Reaction File | The name of a text file containing the internal reactions of a stoichiometric network. See FBA Reaction Files for further information. |
| Constraints File | The name of a text file containing the user-defined flux constraints. See Constraints Files for further information. |
| Program Solver | The name of the program solver to be used to compute the flux intervals. See Program Solvers for further information. |
| Output File Name | The name of the file to which the computed flux intervals will be written. See Single-Flux Interval Vector Files for further information. |
|  |
| Optional Keywords | Possible Values |
| Data Headers | The data headers of the specified output file. See Reaction Name Data Headers for further information. |
| Zero Cutoff | The amount by which a computed lower bound can *exceed* a computed upper bound. See Zero Cutoffs for further information. |
| Constraint Tolerance | The amount by which the linear program solver is allowed to violate the defined flux constraints. See Constraint Tolerances for further information. |
| Safety Level | The safety level at which the optimizations will be performed. See Safety Levels for further information. |
| Program Solver Parameter File | The name of the file containing parameters for the linear program solver. See Program Solver Parameter Files for further information. |

|  |
| --- |
|  |

|  |
| --- |
| Examples Click here for an example. |
