# Supplementary material for: The Systems Biology Research Tool: evolvable open-source software
Source: BMC Syst Biol. 2008 Jun 29;2:55. doi: 10.1186/1752-0509-2-55 (PMC2446383; doi:10.1186/1752-0509-2-55)
Supplement: Additional file 1 — SBRT Archive. An archive of the current version of the Systems Biology Research Tool. [file 1752-0509-2-55-S1.zip › sbrt-1.4.0/doc/users_guide/fba/processes/data_analysis/Flux_Activity_Analysis.html]

Flux Activity Analysis - Systems Biology Research Tool


|  |
| --- |
| > User's Guide > Flux Balance Analysis > Data Analysis |
|  |
| Flux Activity Analysis  This process is used to analyse the activities of fluxes in a collection of flux vectors. A flux is considered to be *inactive* if it equals zero and *active* otherwise. For each vector of fluxes supplied to this process, the number of active and inactive fluxes will be written to a specified output file.  Here is the set of keywords this process understands, along with a description of their possible corresponding values. See the command line documentation for more information about keyword-value pairs. |

  


|  |  |
| --- | --- |
| Required Keywords | Possible Values |
| Process Name File | The name of the file where process names are defined. See  Process Name Files for further information. |
| Process | The name defined in the specified process name file.  FBA Flux Activity Analysis is the default value. |
| Reaction File | The name of a text file containing the internal reactions of a stoichiometric network. See FBA Reaction Files for further information. |
| Flux Vector File | The name of a text file containing the flux vectors whose activities are to be analyzed. See Multiple-Flux Vectors Files for further information. |
| Flux Vector File Format | The format of the specified flux vector file. See File Formats for further information. |
| Zero Cutoff | The amount by which fluxes can differ from zero, but still be considered equal to zero. See Zero Cutoffs for further information. |
| Activity Count File Name | The desired name of the file to which the number of active and inactive fluxes in each vector will be written. The *i*-th line in this file corresponds to the *i*-th flux vector in the input file. |

|  |
| --- |
|  |

|  |
| --- |
| Examples Click here for an example. |
