# Supplementary material for: The Systems Biology Research Tool: evolvable open-source software
Source: BMC Syst Biol. 2008 Jun 29;2:55. doi: 10.1186/1752-0509-2-55 (PMC2446383; doi:10.1186/1752-0509-2-55)
Supplement: Additional file 1 — SBRT Archive. An archive of the current version of the Systems Biology Research Tool. [file 1752-0509-2-55-S1.zip › sbrt-1.4.0/doc/users_guide/fba/processes/data_analysis/index.html]

FBA Data Analysis - Systems Biology Research Tool


|  |
| --- |
| > User's Guide > Flux Balance Analysis |
|  |
| Data Analysis |

  


|  |  |
| --- | --- |
| Processes | Brief Descriptions |
| Flux Activity Analysis | Used to analyze the activity of fluxes in a collection of flux vectors. |
| Flux Plasticity Analysis | Used to analyse the plasticity of fluxes in a collection of flux interval vectors. |
