# Supplementary material for: The Systems Biology Research Tool: evolvable open-source software
Source: BMC Syst Biol. 2008 Jun 29;2:55. doi: 10.1186/1752-0509-2-55 (PMC2446383; doi:10.1186/1752-0509-2-55)
Supplement: Additional file 1 — SBRT Archive. An archive of the current version of the Systems Biology Research Tool. [file 1752-0509-2-55-S1.zip › sbrt-1.4.0/doc/users_guide/fba/processes/data_analysis/Flux_Plasticity_Analysis.html]

Flux Plasticity Analysis - Systems Biology Research Tool


|  |
| --- |
| > User's Guide > Flux Balance Analysis > Data Analysis |
|  |
| Flux Plasticity Analysis  This process is used to analyze the *plasticity* of fluxes in a collection of flux interval vectors. A flux is considered:   - *never-active* if its interval is [0, 0]. - *potentially-active* if its interval is [0, x], where   x > 0. - *always-active* if its interval is [x, y], where x   > 0 and y ≥ x. - *plastic* if its range is greater than zero. - *rigid* if its range equals zero.   For each vector of flux intervals supplied to this process, the number of fluxes in the above categories will be written to a specified output file. Additionally, the names of the reactions whose fluxes are never-active, potentially-active, and always-active can be recorded as well, for each flux interval vector.  Here is the set of keywords this process understands, along with a description of their possible corresponding values. See the command line documentation for more information about keyword-value pairs. |

  


|  |  |
| --- | --- |
| Required Keywords | Possible Values |
| Process Name File | The name of the file where process names are defined. See  Process Name Files for further information. |
| Process | The name defined in the specified process name file.  FBA Flux Plasticity Analysis is the default value. |
| Reaction File | The name of a text file containing the internal reactions of a stoichiometric network. See FBA Reaction Files for further information. |
| Flux Interval File | The name of the file containing the sets of flux intervals to be analyzed. See Multiple-Flux Interval Vectors Files for further information. |
| Zero Cutoff | The amount by which values can differ from zero, but still be considered equal to zero. See Zero Cutoffs for further information. |
| Output File Name | The name of the file to which the results of the analysis will be written. The *i*-th line in this file corresponds to the *i*-th vector of flux intervals in the input file. |
|  |
| Optional Keywords | Possible Values |
| Always-Active Reaction File Name | The desired name of the file to which the sets of always-active reactions will be written. The *i*-th line in this file corresponds to the *i*-th set of flux intervals in the input file. |
| Potentially-Active Reaction File Name | The desired name of the file to which the sets of potentially-active reactions will be written. The *i*-th line in this file corresponds to the *i*-th set of flux intervals in the input file. |
| Never-Active Reaction File Name | The desired name of the file to which the sets of never-active reactions will be written. The *i*-th line in this file corresponds to the *i*-th set of flux intervals in the input file. |

|  |
| --- |
|  |

|  |
| --- |
| Examples Click here for an example. |
