# Supplementary material for: The Systems Biology Research Tool: evolvable open-source software
Source: BMC Syst Biol. 2008 Jun 29;2:55. doi: 10.1186/1752-0509-2-55 (PMC2446383; doi:10.1186/1752-0509-2-55)
Supplement: Additional file 1 — SBRT Archive. An archive of the current version of the Systems Biology Research Tool. [file 1752-0509-2-55-S1.zip › sbrt-1.4.0/doc/users_guide/fba/processes/pathway_id/Extreme_Current_Id.html]

Extreme Current Identification - Systems Biology Research
Tool


|  |
| --- |
| > User's Guide > Flux Balance Analysis > Pathway Identification |
|  |
| Extreme Current Identification  This process is used to identify all extreme currents in a stoichiometric network. Extreme currents are essentially equivalent to extreme pathways [1]. The Systems Biology Research Tool uses stoichiometric networks where all fluxes are constrained to lie in the positive orthant of flux space, including exchange fluxes (see FBA Reaction Files for more information). This formulation was described by Clarke in the early 1980s [2]. The edges of this type of flux space have been termed *extreme currents* [1, 3], whereas those of the more commonly used formulation have been termed *extreme pathways* [1, 3].  The algorithm used to compute extreme currents is a variation of that described by Schilling et. al. [3]. It is susceptible to combinatorial explosion, so it may not work well for large stoichiometric networks. **References**  |  |  | | --- | --- | | 1. | Wagner, C. and Urbanczik, R. (2005). *The geometry of the flux cone of a metabolic network.* Biophys. J. 89, 3837-3845. | | 2. | Clarke, B. L. (1981). *Complete set of steady states for the general stoichiometric dynamical system.* J. Chem. Phys. 75, 4970-4979. | | 3. | Schilling, C. H., Letscher, D., and Palsson, B.Ø. (2000). *Theory for the systemic definition of metabolic pathways and their use in interpreting metabolic function from a pathway-oriented perspective.* J. Theor. Biol., 203, 229-248. |   Here is the set of keywords this process understands, along with a description of their possible corresponding values. |

  


|  |  |
| --- | --- |
| Required Keywords | Possible Values |
| Process Name File | The name of the file where process names are defined. See  Process Name Files for further information. |
| Process | The name defined in the specified process name file.  FBA Extreme Current Identification is the default value. |
| Reaction File | The name of a text file containing the internal reactions of a stoichiometric network. See FBA Reaction Files for further information. |
| Output File Name | The name of the file to which the extreme currents will be written. See  Multiple-Flux Vectors Files for further information. |

|  |
| --- |
|  |

|  |
| --- |
| Examples Click here for an example. |
