# Supplementary material for: The Systems Biology Research Tool: evolvable open-source software
Source: BMC Syst Biol. 2008 Jun 29;2:55. doi: 10.1186/1752-0509-2-55 (PMC2446383; doi:10.1186/1752-0509-2-55)
Supplement: Additional file 1 — SBRT Archive. An archive of the current version of the Systems Biology Research Tool. [file 1752-0509-2-55-S1.zip › sbrt-1.4.0/doc/users_guide/fba/processes/pathway_id/index.html]

FBA Pathway ID - Systems Biology Research Tool


|  |
| --- |
| > User's Guide > Flux Balance Analysis |
|  |
| Pathway Identification |

  


|  |  |
| --- | --- |
| Processes | Brief Descriptions |
| Extreme Current Identification | Used to identify the extreme currents in stoichiometric networks. |
| WW Network Reduction | Used to reduce the size of stoichiometric networks for the purpose of identifying the cycles they contain. |
| MS Network Reduction | Used to reduce the size of stoichiometric networks for the purpose of identifying the cycles they contain. |
| SLP Cycle Identification | Used to identify the cycles in stoichiometric networks. |
