# Supplementary material for: The Systems Biology Research Tool: evolvable open-source software
Source: BMC Syst Biol. 2008 Jun 29;2:55. doi: 10.1186/1752-0509-2-55 (PMC2446383; doi:10.1186/1752-0509-2-55)
Supplement: Additional file 1 — SBRT Archive. An archive of the current version of the Systems Biology Research Tool. [file 1752-0509-2-55-S1.zip › sbrt-1.4.0/doc/users_guide/fba/processes/pathway_id/WW_Network_Reduction.html]

WW Network Reduction - Systems Biology Research Tool


|  |
| --- |
| > User's Guide > Flux Balance Analysis > Pathway Identification |
|  |
| WW Network Reduction This process is used to reduce the size of stoichiometric networks for the purpose of identifying the cycles, type III extreme pathways [1], they contain. The algorithm used by this process was described by Wright and Wagner in 2008 (submitted). The output of this process can be used as input for Extreme Current Identification or as input for Metatool using the Metatool File Writer. **References**  |  |  | | --- | --- | | 1. | Schilling, C. H., Letscher, D., and Palsson, B.Ø. (2000). *Theory for the systemic definition of metabolic pathways and their use in interpreting metabolic function from a pathway-oriented perspective.* J. Theor. Biol., 203: 229-248. |   Here is the set of keywords this process understands, along with a description of their possible corresponding values. See the command line documentation for more information about keyword-value pairs. |

  


|  |  |
| --- | --- |
| Required Keywords | Possible Values |
| Process Name File | The name of the file where process names are defined. See  Process Name Files for further information. |
| Process | The name defined in the specified process name file.  FBA WW Network Reduction is the default value. |
| Reaction File | The name of a text file containing the internal reactions of a stoichiometric network. See FBA Reaction Files for further information. |
| Program Solver | The name of the program solver to be used during network reduction. See Program Solvers for further information. |
| Output File Name | The name of the file to which the reduced fluxome will be written. See  FBA Reaction Files for further information. |
|  |
| Optional Keywords | Possible Values |
| Program Solver Parameter File | The name of the file containing parameters for the linear program solver. See Program Solver Parameter Files for further information. |

|  |
| --- |
|  |

|  |
| --- |
| Examples Click here for an example. |
