# Supplementary material for: The Systems Biology Research Tool: evolvable open-source software
Source: BMC Syst Biol. 2008 Jun 29;2:55. doi: 10.1186/1752-0509-2-55 (PMC2446383; doi:10.1186/1752-0509-2-55)
Supplement: Additional file 1 — SBRT Archive. An archive of the current version of the Systems Biology Research Tool. [file 1752-0509-2-55-S1.zip › sbrt-1.4.0/doc/users_guide/fba/processes/pathway_id/MS_Network_Reduction.html]

MS Network Reduction - Systems Biology Research Tool


|  |
| --- |
| > User's Guide > Flux Balance Analysis > Pathway Identification |
|  |
| MS Network Reduction This process is used to reduce the size of stoichiometric networks for the purpose of identifying the cycles, type III extreme pathways [1], they contain. The algorithm used was described by Mahadevan and Schilling in 2003 [2]. The output of this process can be used as input for Extreme Current Identification or as input for Metatool using the Metatool File Writer. **References**  |  |  | | --- | --- | | 1. | Schilling, C. H., Letscher, D., and Palsson, B.Ø. (2000). *Theory for the systemic definition of metabolic pathways and their use in interpreting metabolic function from a pathway-oriented perspective.* J. Theor. Biol., 203: 229-248. | | 2. | Mahadevan R., Schilling C. H. (2003) *The effects of alternate optimal solutions in constraint-based genome-scale metabolic models.* Metab. Eng., 5(4): 264-76. |   Here is the set of keywords this process understands, along with a description of their possible corresponding values. See the command line documentation for more information about keyword-value pairs. |

  


|  |  |
| --- | --- |
| Required Keywords | Possible Values |
| Process Name File | The name of the file where process names are defined. See  Process Name Files for further information. |
| Process | The name defined in the specified process name file.  FBA MS Network Reduction is the default value. |
| Reaction File | The name of a text file containing the internal reactions of a stoichiometric network. See FBA Reaction Files for further information. |
| Program Solver | The name of the program solver to be used during network reduction. See Program Solvers for further information. |
| Output File Name | The name of the file to which the reduced fluxome will be written. See  FBA Reaction Files for further information. |
|  |
| Optional Keywords | Possible Values |
| Program Solver Parameter File | The name of the file containing parameters for the linear program solver. See Program Solver Parameter Files for further information. |

|  |
| --- |
|  |

|  |
| --- |
| Examples Click here for an example. |
