# Supplementary material for: The Systems Biology Research Tool: evolvable open-source software
Source: BMC Syst Biol. 2008 Jun 29;2:55. doi: 10.1186/1752-0509-2-55 (PMC2446383; doi:10.1186/1752-0509-2-55)
Supplement: Additional file 1 — SBRT Archive. An archive of the current version of the Systems Biology Research Tool. [file 1752-0509-2-55-S1.zip › sbrt-1.4.0/doc/users_guide/fba/processes/flux_space_sampling/Random_Constraint_Generator.html]

Random Constraint Generator - Systems Biology Research
Tool


|  |
| --- |
| > User's Guide > Flux Balance Analysis > Flux Space Sampling |
|  |
| Random Constraint Generator This process is used to generate sets of random flux constraints. There are many conceivable ways to create random flux constraints, but this process uses only one such technique.  Each set of randomly generated flux constraints will be of size *n*. Some number of fluxes, *k*, in each set will be "active", and some number of fluxes, *n-k*, will be "inactive". The assignment of "active" or "inactive" is randomly chosen for each flux in each generated set. The value of *n* is equal to the number of reactions specified as data headers, and the value of *k* must be provided. The intervals used to represent active and inactive fluxes must be specified as well.  Here is the set of keywords this process understands, along with a description of their possible corresponding values. See the command line documentation for more information about keyword-value pairs. |

  


|  |  |
| --- | --- |
| Required Keywords | Possible Values |
| Process Name File | The name of the file where process names are defined. See  Process Name Files for further information. |
| Process | The name defined in the specified process name file.  FBA Random Constraint Generator is the default value. |
| Reaction File | The name of a text file containing the internal reactions of a stoichiometric network. See FBA Reaction Files for further information. |
| Iterations | The number of sets of constraints to be generated. |
| Seed | The seed for the random number generator. |
| Number of Active Fluxes | The number of *active* fluxes per set. |
| Active Constraint | The interval used to represent active fluxes. |
| Inactive Constraint | The interval used to represent inactive fluxes. |
| Data Headers | The reaction name data headers of the output file. |
| Output File Name | The desired name of the constraint variation file to be created. |

|  |
| --- |
|  |

|  |
| --- |
| Examples Click here for an example. |
