# Supplementary material for: The Systems Biology Research Tool: evolvable open-source software
Source: BMC Syst Biol. 2008 Jun 29;2:55. doi: 10.1186/1752-0509-2-55 (PMC2446383; doi:10.1186/1752-0509-2-55)
Supplement: Additional file 1 — SBRT Archive. An archive of the current version of the Systems Biology Research Tool. [file 1752-0509-2-55-S1.zip › sbrt-1.4.0/doc/users_guide/fba/processes/flux_space_sampling/index.html]

FBA Flux Space Sampling - Systems Biology Research Tool


|  |
| --- |
| > User's Guide > Flux Balance Analysis |
|  |
| Flux Space Sampling |
|  |
| These processes are used to randomly create or sample flux spaces. |

  
  


|  |  |
| --- | --- |
| Processes | Brief Descriptions |
| Random Constraint Generator | Used to generate random flux constraints. |
| Random Objective Function Generator | Used to generate random objective functions. |
| Initial Point Generator | Used to compute an initial flux vector for use in CD Hit-and-Run Analysis. |
| Coordinate Direction Hit-and-Run Analysis | Used to compute random, uniformly-distributed flux vectors from the interior flux space. |
| Space Variation-Initial Point Generator | Used to compute initial flux vectors for use in Space Variation-CD Hit-and-Run Analysis. |
| Space Variation-Coordinate Direction Hit-and-Run Analysis | Used to compute random, uniformly-distributed flux vectors from the interiors of multiple flux spaces. |

  
  
