# Supplementary material for: The Systems Biology Research Tool: evolvable open-source software
Source: BMC Syst Biol. 2008 Jun 29;2:55. doi: 10.1186/1752-0509-2-55 (PMC2446383; doi:10.1186/1752-0509-2-55)
Supplement: Additional file 1 — SBRT Archive. An archive of the current version of the Systems Biology Research Tool. [file 1752-0509-2-55-S1.zip › sbrt-1.4.0/doc/users_guide/fba/processes/flux_space_sampling/SV_CD_Har_Analysis.html]

FBA Space Variation-Coordinate Direction Hit-and-Run
Analysis - Systems Biology Research Tool


|  |
| --- |
| > User's Guide > Flux Balance Analysis > Flux Space Sampling |
|  |
| FBA Space Variation-Coordinate Direction Hit-and-Run Analysis This process is used to generate random points in multiple flux spaces. See the coordinate directions hit-and-run algorithm for additional information.  This process must be supplied with the following:   1. Initial flux vectors that lie in the interior of the    provided flux spaces. These can be obtained with the Space Variation-Initial    Point Generator. 2. The flux intervals used to define each flux space. This    should be the same Flux Interval File that was supplied    to the Space Variation-Initial Point Generator. 3. The solution to *Sv = 0*. This can be    obtained with the FBA    System Solver if Mathematica is    available. If Mathematica is not available, the Network Information    Gatherer can be used to generate the system of equations    corresponding to *Sv = 0*, so that the solution can be    obtained with another program of your choice.   The random flux vectors generated by this process are the result of multiple "random walks" through flux space - one walk along the axis of each free variable. Stated differently, *n* walks are made through an *n* dimensional flux space (where *n* is the number of free variables) to create the next *random point*. Each *random point* can be written to a specified output file, or each *i*-th random point can be written using the optional *points-per-point* keyword.  Here is the set of keywords this process understands, along with a description of their possible corresponding values. See the command line documentation for more information about keyword-value pairs. |

  


|  |  |
| --- | --- |
| Required Keywords | Possible Values |
| Process Name File | The name of the file where process names are defined. See  Process Name Files for further information. |
| Process | The name defined in the specified process name file.  FBA Space Variation-CD Hit-and-Run is the default value. |
| Reaction File | The name of a text file containing the internal reactions of a stoichiometric network. See FBA Reaction Files for further information. |
| Flux Interval File | The name of a text file containing the intervals of fluxes defining multiple flux spaces. See Multiple-Flux Interval Vectors Files for further information. |
| Initial Point File | The name of the file containing the initial flux vectors in the interiors of the provided flux spaces. See Multiple-Flux Vectors Files for further information. |
| System Solution File | The solution to the system of linear equations formed by the specified stoichiometric network. See Linear System Solution Files for additional information. |
| Iterations | The number of random flux vectors to be written to each of the specified output files. |
| Output File Name File | The name of a file containing the desired names of the files to which the random flux vectors will be written. See File Name Files and Multiple-Flux Vectors Files for additional information. |
| Obstructed Moves File Name | The desired name of the file to which the number of obstructed moves per flux space will be written. |
|  |
| Optional Keywords | Possible Values |
| Data Headers | The data headers of the specified output file. The default value is all reaction names. See Reaction Name Data Headers for further information. |
| File Format | The desired format of the specified output file. The default value is Text. See File Formats for additional information. |
| Constraint Tolerance | The amount by which a generated point may lay outside of the defined constraints. The default value is 1E-9. See Constraint Tolerances for more information. |
| Points Per Point | The number of random points to generate for each point written to the specified output file. The default value is 1. |
| Minimum Chord Length | The minimum length of an unobstructed direction. The default value is 1E-6. |
| Seed | The seed for the pseudo-random number generator. The default value is the current time in milliseconds. |
| Maximum Obstructed Moves | The number of obstructed moves to allow before the process is terminated prematurely. The default value is 0. |

|  |
| --- |
|  |

|  |
| --- |
| Examples Click here for an example. |
