# Supplementary material for: The Systems Biology Research Tool: evolvable open-source software
Source: BMC Syst Biol. 2008 Jun 29;2:55. doi: 10.1186/1752-0509-2-55 (PMC2446383; doi:10.1186/1752-0509-2-55)
Supplement: Additional file 1 — SBRT Archive. An archive of the current version of the Systems Biology Research Tool. [file 1752-0509-2-55-S1.zip › sbrt-1.4.0/doc/users_guide/fba/processes/flux_space_sampling/Initial_Point_Generator.html]

Initial Point Generator - Systems Biology Research Tool


|  |
| --- |
| > User's Guide > Flux Balance Analysis > Flux Space Sampling |
|  |
| Initial Point Generator This process is used to generate initial points for algorithms that randomly sample flux vectors from the interior of flux space. This process uses the initial point generation technique described in the geometry section of the User's Guide.  The flux intervals supplied to this process must be the intervals on which each flux truly lies. If they are not, the results produced by this process will be incorrect. For all but the simplest systems, these intervals must be computed beforehand using one of the flux variability processes. The flux space must also be bounded, that is, infinite lower or upper bounds on fluxes are not allowed. These restrictions result from the way in which the positioning space is created for each flux. The extreme points used to define each positioning space are:   | (P, V) | | --- | | ( *Pmax* , *Vmin* ) | | ( 1.0 , *Vmin* + 0.001 *L* ) | | ( 0.5 , *Vmin* + 0.010 *L* ) | | ( 0.1 , *Vmin* + 0.050 *L* ) | | ( 0.0 , *Vmin* + 0.500 *L* ) | | ( 0.1 , *Vmin* + 0.950 *L* ) | | ( 0.5 , *Vmin* + 0.990 *L* ) | | ( 1.0 , *Vmin* + 0.999 *L* ) | | ( *Pmax* , *Vmax* ) |   where *Vmin* and *Vmax* are the lower and upper bounds on each flux, respectively, and *L = Vmax - Vmin*. If the range of a flux is zero, that is *L = 0*, a P-V space is not created for that flux. The value for *Pmax* can be specified by the user, since the default value is sometimes too high for some linear program solvers. The largest value that still produces a feasible solution should be chosen.  Here is the set of keywords this process understands, along with a description of their possible corresponding values. See the command line documentation for more information about keyword-value pairs. |

  


|  |  |
| --- | --- |
| Required Keywords | Possible Values |
| Process Name File | The name of the file where process names are defined. See  Process Name Files for further information. |
| Process | The name defined in the specified process name file.  FBA Initial Point Generator is the default value. |
| Reaction File | The name of a text file containing the internal reactions of a stoichiometric network. See FBA Reaction Files for further information. |
| Flux Interval File | The name of a text file containing the intervals of each flux in the specified stoichiometric network. See Single-Flux Interval Vector Files for further information. |
| Program Solver | The name of the program solver to be used to compute the minimum value of Σ Pi. See Program Solvers for further information. |
|  |
| Optional Keywords | Possible Values |
| Flux Vector File Name | The desired name of the file to which the computed flux vector will be written. See Single-Flux Vector Files for further information. |
| P Max | The numerical value of positioning variables at the edges of the convex polytope. The default is 1E15. |
| P Vector File Name | The desired name of the file to which the computed vector of positioning variable values will be written. |
| Constraint Tolerance | The amount by which the linear program solver is allowed to violate the defined flux intervals. See Constraint Tolerances for further information. |
| Safety Level | The safety level at which the optimizations will be performed. See Safety Levels for further information. |
| Program Solver Parameter File | The name of the file containing parameters for the linear program solver. See Program Solver Parameter Files for further information. |

|  |
| --- |
|  |

|  |
| --- |
| Examples Click here for an example. |
