# Supplementary material for: The Systems Biology Research Tool: evolvable open-source software
Source: BMC Syst Biol. 2008 Jun 29;2:55. doi: 10.1186/1752-0509-2-55 (PMC2446383; doi:10.1186/1752-0509-2-55)
Supplement: Additional file 1 — SBRT Archive. An archive of the current version of the Systems Biology Research Tool. [file 1752-0509-2-55-S1.zip › sbrt-1.4.0/doc/users_guide/fba/processes/flux_space_sampling/Random_Obj_Function_Generator.html]

Random Objective Function Generator - Systems Biology
Research Tool


|  |
| --- |
| > User's Guide > Flux Balance Analysis > Flux Space Sampling |
|  |
| Random Objective Function Generator This process is used to generate random objective functions for use in flux balance analysis. There are many conceivable ways to create random objective functions, but this process uses only one such technique.  The objective functions produced by this process are linear combinations of reaction names from the provided stoichiometric network. Every reaction name is present in each objective function, and the coefficient of each term is randomly chosen from an (approximately) uniform distribution along the interval [-1, +1). The constant of each objective function is 0. Stated differently, each objective function has the form Σ αi ri + 0, where *r* denotes a reaction name and α denotes the randomly chosen coefficient.  Here is the set of keywords this process understands, along with a description of their possible corresponding values. See the command line documentation for more information about keyword-value pairs. |

  


|  |  |
| --- | --- |
| Required Keywords | Possible Values |
| Process Name File | The name of the file where process names are defined. See  Process Name Files for further information. |
| Process | The name defined in the specified process name file.  FBA Random Objective Function Generator is the default value. |
| Reaction File | The name of a text file containing the internal reactions of a stoichiometric network. See FBA Reaction Files for further information. |
| Iterations | The number of random objective functions to generate. |
| Seed | The seed for the random number generator. |
| Output File Name | The desired name of the file to which the generated objective functions will be written. See Objective Function Files for more information. |
|  |
| Optional Keywords | Possible Values |
| File Format | The desired text-based format of the output file. |

|  |
| --- |
|  |

|  |
| --- |
| Examples Click here for an example. |
