# Supplementary material for: The Systems Biology Research Tool: evolvable open-source software
Source: BMC Syst Biol. 2008 Jun 29;2:55. doi: 10.1186/1752-0509-2-55 (PMC2446383; doi:10.1186/1752-0509-2-55)
Supplement: Additional file 1 — SBRT Archive. An archive of the current version of the Systems Biology Research Tool. [file 1752-0509-2-55-S1.zip › sbrt-1.4.0/doc/users_guide/fba/processes/optimization/Reaction_Deletion.html]

Reaction Deletion - Systems Biology Research Tool


|  |
| --- |
| > User's Guide > Flux Balance Analysis > Optimization |
|  |
| FBA Reaction Deletion This process is used to compute the effects of deleting sets of reactions in a stoichiometric network. This is not the same as deleting sets of catalysts, since a single catalyst can catalyze multiple reactions, and a single reaction can be catalyzed by multiple catalysts. The following steps are executed during this process:   |  |  | | --- | --- | | 1. | The next set of reaction names contained in the specified *reaction deletion file* are retrieved, and the fluxes of their corresponding reactions are constrained to the interval [0, 0]. | | 2. | The specified objective function is optimized in the specified sense, or direction, and the results are written to the next line of the specified output file. | | 3. | If another set of reaction names is present in the *reaction deletion file*, Steps 1 and 2 are repeated. |   Here is the set of keywords this process understands, along with a description of their possible corresponding values. |

  


|  |  |
| --- | --- |
| Required Keywords | Possible Values |
| Process Name File | The name of the file where process names are defined. See  Process Name Files for further information. |
| Process | The name defined in the specified process name file.  FBA Reaction Deletion is the default value. |
| Reaction File | The name of a text file containing the internal reactions of a stoichiometric network. See FBA Reaction Files for further information. |
| Constraints File | The name of a text file containing the user-defined flux constraints. See Constraints Files for further information. |
| Reaction Deletion File | The name of a text file containing the names of the reactions to be deleted. See Reaction Name Files for further information. |
| Objective Function | The objective function to be optimized for each set of constraints and deletions. See FBA Objective Functions for further information. |
| Optimization Sense | The sense, or direction, in which the optimal values will be computed. See Optimization Senses for further information. |
| Program Solver | The name of the program solver to be used to compute the optimal values. See Program Solvers for further information. |
| Output File Name | The name of the file to which the computed values will be written. See  FBA Multiple-Optimization Output Files for further information. |
| Data Headers | The data headers of the specified output file. See FBA Optimization Data Headers for further information. |
|  |
| Optional Keywords | Possible Values |
| Constraint Tolerance | The amount by which the linear program solver is allowed to violate the defined flux constraints. See Constraint Tolerances for further information. |
| Zero Cutoff | The amount by which fluxes can differ from zero, but still be considered equal to zero. See Zero Cutoffs for further information. |
| Safety Level | The safety level at which the optimizations will be performed. See Safety Levels for further information. |
| Program Solver Parameter File | The name of the file containing parameters for the linear program solver. See Program Solver Parameter Files for further information. |

|  |
| --- |
|  |

|  |
| --- |
| Examples Click here for an example. |
