# Supplementary material for: The Systems Biology Research Tool: evolvable open-source software
Source: BMC Syst Biol. 2008 Jun 29;2:55. doi: 10.1186/1752-0509-2-55 (PMC2446383; doi:10.1186/1752-0509-2-55)
Supplement: Additional file 1 — SBRT Archive. An archive of the current version of the Systems Biology Research Tool. [file 1752-0509-2-55-S1.zip › sbrt-1.4.0/doc/users_guide/fba/processes/optimization/FBA_Optimization.html]

FBA Optimization - Systems Biology Research Tool


|  |
| --- |
| > User's Guide > Flux Balance Analysis > Optimization |
|  |
| FBA Optimization  This process is used to perform a basic FBA optimization.  Here is the set of keywords this process understands, along with a description of their possible corresponding values. See the command line documentation for more information about keyword-value pairs. |

  


|  |  |
| --- | --- |
| Required Keywords | Possible Values |
| Process Name File | The name of the file where process names are defined. See  Process Name Files for further information. |
| Process | The name defined in the specified process name file.  FBA Optimization is the default value. |
| Reaction File | The name of a text file containing the internal reactions of a stoichiometric network. See FBA Reaction Files for further information. |
| Constraints File | The name of a text file containing the user-defined flux constraints. See Constraints Files for further information. |
| Objective Function | The objective function to be optimized. See FBA Objective Functions for further information. |
| Optimization Sense | The sense, or direction, in which the objective function will be optimized. See Optimization Senses for further information. |
| Program Solver | The name of the program solver to be used. See Program Solvers for further information. |
|  |
| Optional Keywords | Possible Values |
| Output File Name | The name of the file to which the computed values will be written. See FBA Single-Optimization Output Files for further information. |
| Data Headers | The data headers of the specified output file. See FBA Optimization Data Headers for further information. |
| Constraint Tolerance | The amount by which the linear program solver is allowed to violate the defined flux constraints. See Constraint Tolerances for further information. |
| Safety Level | The safety level at which the optimizations will be performed. See Safety Levels for further information. |
| Program Solver Parameter File | The name of the file containing parameters for the linear program solver. See Program Solver Parameter Files for further information. |

|  |
| --- |
|  |

|  |
| --- |
| Examples Click here for an example. |
