# Supplementary material for: The Systems Biology Research Tool: evolvable open-source software
Source: BMC Syst Biol. 2008 Jun 29;2:55. doi: 10.1186/1752-0509-2-55 (PMC2446383; doi:10.1186/1752-0509-2-55)
Supplement: Additional file 1 — SBRT Archive. An archive of the current version of the Systems Biology Research Tool. [file 1752-0509-2-55-S1.zip › sbrt-1.4.0/doc/users_guide/fba/processes/optimization/Objective_Function.html]

Objective Function Analysis - Systems Biology Research
Tool


|  |
| --- |
| > User's Guide > Flux Balance Analysis > Optimization |
|  |
| FBA Objective Function Analysis  This process is used to compute the optimal values of a list of objective functions. The following steps are executed during this process:   |  |  | | --- | --- | | 1. | The next objective function contained in the specified *objective function file* is retrieved, optimized in the specified direction, and the results are written to the next line of the specified output file. | | 2. | If another objective function is present in the *objective function file*, Step 1 is repeated. |   Here is the set of keywords this process understands, along with a description of their possible corresponding values. |

  


|  |  |
| --- | --- |
| Required Keywords | Possible Values |
| Process Name File | The name of the file where process names are defined. See  Process Name Files for further information. |
| Process | The name defined in the specified process name file.  FBA Objective Function Analysis is the default value. |
| Reaction File | The name of a text file containing the internal reactions of a stoichiometric network. See FBA Reaction Files for further information. |
| Constraints File | The name of a text file containing the user-defined flux constraints. See Constraints Files for further information. |
| Objective Function File | The name of a text file containing the objective functions to be optimized. See Objective Function Files for further information. |
| Optimization Sense | The sense, or direction, in which the optimal values will be computed. See Optimization Senses for further information. |
| Program Solver | The name of the program solver to be used to compute the optimal values. See Program Solvers for further information. |
| Output File Name | The name of the file to which the computed values will be written. See  FBA Multiple-Optimization Output Files for further information. |
| Data Headers | The data headers of the specified output file. See FBA Optimization Data Headers for further information. |
|  |
| Optional Keywords | Possible Values |
| Constraint Tolerance | The amount by which the linear program solver is allowed to violate the defined flux constraints. See Constraint Tolerances for further information. |
| Safety Level | The safety level at which the optimization will be performed. See Safety Levels for further information. |
| Program Solver Parameter File | The name of the file containing parameters for the linear program solver. See Program Solver Parameter Files for further information. |
| Objective Function File Format | The format of the objective function file. See Objective Function Files for further information. |

|  |
| --- |
|  |

|  |
| --- |
| Examples Click here for an example. |
