# Supplementary material for: The Systems Biology Research Tool: evolvable open-source software
Source: BMC Syst Biol. 2008 Jun 29;2:55. doi: 10.1186/1752-0509-2-55 (PMC2446383; doi:10.1186/1752-0509-2-55)
Supplement: Additional file 1 — SBRT Archive. An archive of the current version of the Systems Biology Research Tool. [file 1752-0509-2-55-S1.zip › sbrt-1.4.0/doc/users_guide/fba/processes/optimization/index.html]

FBA Optimization - Systems Biology Research Tool


|  |
| --- |
| > User's Guide > Flux Balance Analysis |
|  |
| Optimization |
| These processes are used to optimize objective functions under a variety of conditions. |

  


|  |  |
| --- | --- |
| Processes | Brief Descriptions |
| FBA Optimization | Used to compute the optimal value of a single objective function. |
| Reaction Deletion | Used to compute the effect of deleting reactions in a stoichiometric network. |
| Catalyst Deletion | Used to compute the effect of deleting catalyst in a stoichiometric network. |
| Objective Function Analysis | Used to compute the optimal values of multiple objective functions. |
| Constraint Variation | Used to compute the optimal values of a single objective function for multiple sets of flux constraints. |
| Constraint Variation-Reaction Deletion | Used to compute the combined effects of deleting reactions and varying the flux constraints in a stoichiometric network. |
| Constraint Variation-Catalyst Deletion | Used to compute the combined effects of deleting catalysts and varying the flux constraints in a stoichiometric network. |
| Constraint Variation-Objective Function Analysis | Used to compute the optimal values of multiple objective functions for multiple sets of flux constraints. |
