# Supplementary material for: The Systems Biology Research Tool: evolvable open-source software
Source: BMC Syst Biol. 2008 Jun 29;2:55. doi: 10.1186/1752-0509-2-55 (PMC2446383; doi:10.1186/1752-0509-2-55)
Supplement: Additional file 1 — SBRT Archive. An archive of the current version of the Systems Biology Research Tool. [file 1752-0509-2-55-S1.zip › sbrt-1.4.0/doc/users_guide/fba/processes/utilities/Network_Info_Gatherer.html]

Network Information Gatherer - Systems Biology Research
Tool


|  |
| --- |
| > User's Guide > Flux Balance Analysis > Utilities |
|  |
| Network Information Gatherer This process is used to gather basic information about a stoichiometric network.  Here is the set of keywords this process understands, along with a description of their possible corresponding values. See the command line documentation for more information about keyword-value pairs. |

  


|  |  |
| --- | --- |
| Required Keywords | Possible Values |
| Process Name File | The name of the file where process names are defined. See  Process Name Files for further information. |
| Process | The name defined in the specified process name file.  FBA Network Information Gatherer is the default value. |
| Reaction File | The name of a text file containing the internal reactions of a stoichiometric network. See FBA Reaction Files for further information. |
|  |
| Optional Keywords | Possible Values |
| Reaction-Catalyst File | The name of the file containing all reaction-catalyst associations. See Reaction-Catalyst Association Files for further information. |
| Catalyst File Name | The desired name of the file to which all catalysts will be written. |
| Equivalent Reaction File Name | The desired name of the file to which the names of all stoichiometrically equivalent reactions will be written. See Equivalent Reaction Files for further information. |
| Equation File Name | The desired name of the file to which the system of linear equations formed by the stoichiometric network will be written. See Linear Equation Files for addtional information. |
| Stoichiometry Matrix File Name | The desired name of the file to which the entire stoichiometry matrix will be written. |
| Chemical Species File Name | The desired name of the file to which all chemical species will be written. |
| Reaction Name File Name | The desired name of the file to which all reaction names will be written. |
| Reaction Output File Name | The desired name of the file to which the stoichiometric network will be written. See FBA Reaction Files for more information. |
| Remove Redundant Reactions | An indication that redundant reactions should be removed from the provided stoichiometric network before any other specified operations occur. Yes or No are acceptable values. |

|  |
| --- |
|  |

|  |
| --- |
| Examples Click here for an example. |
