# Supplementary material for: The Systems Biology Research Tool: evolvable open-source software
Source: BMC Syst Biol. 2008 Jun 29;2:55. doi: 10.1186/1752-0509-2-55 (PMC2446383; doi:10.1186/1752-0509-2-55)
Supplement: Additional file 1 — SBRT Archive. An archive of the current version of the Systems Biology Research Tool. [file 1752-0509-2-55-S1.zip › sbrt-1.4.0/doc/users_guide/fba/processes/utilities/Palsson_SBML_File_Translation.html]

Palsson-SBML File Translation - Systems Biology Research
Tool


|  |
| --- |
| > User's Guide > Flux Balance Analysis > Utilities |
|  |
| Palsson-SBML File Translation This process is used to translate files written in the Systems Biology Markup Language (SBML) into FBA Reaction Files and Reaction-Catalyst Association Files. This process is designed for SBML files obtained from Bernhard Palsson's website, but may work for other SBML files as well.  Unfortunately, not all information contained in these SBML files has been standardized, therefore, the files generated by this process may require subsequent modification before they can be used by the Systems Biology Research Tool.  Here is the set of keywords this process understands, along with a description of their possible corresponding values. See the command line documentation for more information about keyword-value pairs. |

  


|  |  |
| --- | --- |
| Required Keywords | Possible Values |
| Process Name File | The name of the file where process names are defined. See  Process Name Files for further information. |
| Process | The name defined in the specified process name file.  Palsson-SBML File Translation is the default value. |
| SBML Input File | The name of the SBML file containing the *in silico* organism. |
| Reaction File Name | The name of the file to which reactions will be written. |
| Gene File Name | The name of the file to which reaction-gene associations will be written. |
| Protein File Name | The name of the file to which reaction-protein associations will be written. |

|  |
| --- |
|  |

|  |
| --- |
| Examples Click here for an example. |
