# Supplementary material for: The Systems Biology Research Tool: evolvable open-source software
Source: BMC Syst Biol. 2008 Jun 29;2:55. doi: 10.1186/1752-0509-2-55 (PMC2446383; doi:10.1186/1752-0509-2-55)
Supplement: Additional file 1 — SBRT Archive. An archive of the current version of the Systems Biology Research Tool. [file 1752-0509-2-55-S1.zip › sbrt-1.4.0/doc/users_guide/fba/processes/utilities/BiGG_SBML_File_Reader.html]

BiGG-SBML File Reader - Systems Biology Research Tool


|  |
| --- |
| > User's Guide > Flux Balance Analysis > Utilities |
|  |
| BiGG-SBML File Reader This process is used to read files written in the Systems Biology Markup Language (SBML). This process is designed for SBML files obtained from the BiGG Database, but may work for other SBML files as well.  Here is the set of keywords this process understands, along with a description of their possible corresponding values. See the command line documentation for more information about keyword-value pairs. |

  


|  |  |
| --- | --- |
| Required Keywords | Possible Values |
| Process Name File | The name of the file where process names are defined. See  Process Name Files for further information. |
| Process | The name defined in the specified process name file.  BiGG-SBML File Reader is the default value. |
| SBML Input File | The name of the SBML file containing the *in silico* organism. |
|  |
| Optional Keywords | Possible Values |
| Reaction File Name | The name of the file to which reactions will be written. |
| Gene File Name | The name of the file to which reaction-gene associations will be written. |

|  |
| --- |
|  |

|  |
| --- |
| Examples Click here for an example. |
