# Supplementary material for: The Systems Biology Research Tool: evolvable open-source software
Source: BMC Syst Biol. 2008 Jun 29;2:55. doi: 10.1186/1752-0509-2-55 (PMC2446383; doi:10.1186/1752-0509-2-55)
Supplement: Additional file 1 — SBRT Archive. An archive of the current version of the Systems Biology Research Tool. [file 1752-0509-2-55-S1.zip › sbrt-1.4.0/doc/users_guide/fba/processes/utilities/index.html]

FBA Utilities - Systems Biology Research Tool


|  |
| --- |
| > User's Guide > Flux Balance Analysis |
|  |
| Utilities |

  


|  |  |
| --- | --- |
| Processes | Brief Descriptions |
| Simple Reaction File Reader | Used to translate files containing a list of chemical reactions into FBA Reaction Files. |
| Palsson-SBML File Reader | Used to read SBML files from Palsson's website. |
| BiGG-SBML File Reader | Used to read SBML files from the BiGG Database. |
| Palsson-SBML File Translation | Used to translate SBML files from Palsson's website into FBA Reaction Files and Reaction-Catalyst Association Files. |
| BiGG-SBML File Translation | Used to translate SBML files from the BiGG Database into FBA Reaction Files and Reaction-Catalyst Association Files. |
| Metatool File Writer | Used to convert FBA Reaction Files into input files for Metatool. |
| Network Information Gatherer | Used to gather basic information about a stoichiometric network. |
| FBA System Solver | Used to solve the equation *Sv = 0*. |

  
  
