# Supplementary material for: The Systems Biology Research Tool: evolvable open-source software
Source: BMC Syst Biol. 2008 Jun 29;2:55. doi: 10.1186/1752-0509-2-55 (PMC2446383; doi:10.1186/1752-0509-2-55)
Supplement: Additional file 1 — SBRT Archive. An archive of the current version of the Systems Biology Research Tool. [file 1752-0509-2-55-S1.zip › sbrt-1.4.0/doc/users_guide/fba/processes/utilities/FBA_System_Solver.html]

FBA System Solver - Systems Biology Research Tool


|  |
| --- |
| > User's Guide > Flux Balance Analysis > Utilities |
|  |
| FBA System Solver This process is used to solve the equation *Sv = 0*, where *S* denotes a stoichiometry matrix and *v* denotes a vector of all fluxes. The stoichiometry matrix is constructed from a provided stoichiometric network, and Mathematica is used to solve the corresponding system of linear equations. See the SBRT's Mathematica documentation for additional information.  Here is the set of keywords this process understands, along with a description of their possible corresponding values. See the command line documentation for more information about keyword-value pairs. |

  


|  |  |
| --- | --- |
| Required Keywords | Possible Values |
| Process Name File | The name of the file where process names are defined. See  Process Name Files for further information. |
| Process | The name defined in the specified process name file.  FBA System Solver is the default value. |
| Reaction File | The name of a text file containing the internal reactions of a stoichiometric network. See FBA Reaction Files for further information. |
| Kernel Link Command | The command used to link to the Mathematica kernel. See the Mathematica documentation for additional information. |
| Output File Name | The desired name of the output file. See Linear System Solution Files for further information. |

|  |
| --- |
|  |

|  |
| --- |
| Examples Click here for an example. |
