# Supplementary material for: The Systems Biology Research Tool: evolvable open-source software
Source: BMC Syst Biol. 2008 Jun 29;2:55. doi: 10.1186/1752-0509-2-55 (PMC2446383; doi:10.1186/1752-0509-2-55)
Supplement: Additional file 1 — SBRT Archive. An archive of the current version of the Systems Biology Research Tool. [file 1752-0509-2-55-S1.zip › sbrt-1.4.0/doc/users_guide/fba/processes/utilities/Simple_Rxn_File_Reader.html]

Simple Reaction File Translation - Systems Biology
Research Tool


|  |
| --- |
| > User's Guide > Flux Balance Analysis > Utilities |
|  |
| Simple Reaction File Translation This process is used to translate files containing a list of chemical reactions into FBA Reaction Files. This can be useful if a list of chemical reactions is stored in a spreadsheet, as was once common. Files written in the Systems Biology Markup Language, however, are becoming the preferred file format.  The format of input files supplied to this process must be the same as for FBA Reaction Files, but with two key differences:   |  |  | | --- | --- | | 1. | The chemical reactions in the input file can be both reversible and irreversible. The arrow <==> must be used to denote reversible reactions, and the arrow --> must be used to denote irreversible reactions. Reversible reactions are broken apart into a pair of forward-reverse irreversible reactions upon translation. | | 2. | Stoichiometric coefficients must *not* be enclosed by parentheses. |   See the file iLL672.txt contained in the example for a complete illustration.  Here is the set of keywords this process understands, along with a description of their possible corresponding values. See the command line documentation for more information about keyword-value pairs. |

  


|  |  |
| --- | --- |
| Required Keywords | Possible Values |
| Process Name File | The name of the file where process names are defined. See  Process Name Files for further information. |
| Process | The name defined in the specified process name file.  FBA Simple Reaction File Translation is the default value. |
| Input Reaction File | The name of the file containing the list of chemical reactions. |
| Output File Name | The desired name of the output file. See FBA Reaction Files for further information. |

|  |
| --- |
|  |

|  |
| --- |
| Examples Click here for an example. |
