# Supplementary material for: The Systems Biology Research Tool: evolvable open-source software
Source: BMC Syst Biol. 2008 Jun 29;2:55. doi: 10.1186/1752-0509-2-55 (PMC2446383; doi:10.1186/1752-0509-2-55)
Supplement: Additional file 1 — SBRT Archive. An archive of the current version of the Systems Biology Research Tool. [file 1752-0509-2-55-S1.zip › sbrt-1.4.0/doc/users_guide/fba/processes/utilities/Metatool_File_Writer.html]

Metatool File Writer - Systems Biology Research Tool


|  |
| --- |
| > User's Guide > Flux Balance Analysis > Utilities |
|  |
| Metatool File Writer This process is used to convert FBA Reaction Files into input files for Metatool. Note that the Systems Biology Research Tool considers all reactions to be irreversible, including exchange reactions. This differs from the convention used by Metatool, where exchange reactions are bidirectional.  Here is the set of keywords this process understands, along with a description of their possible corresponding values. See the command line documentation for more information about keyword-value pairs. |

  


|  |  |
| --- | --- |
| Required Keywords | Possible Values |
| Process Name File | The name of the file where process names are defined. See  Process Name Files for further information. |
| Process | The name defined in the specified process name file.  Metatool File Writer is the default value. |
| Reaction File | The name of a text file containing the internal reactions of a stoichiometric network. See FBA Reaction Files for further information. |
| Output File Name | The desired name of the generated Metatool file. |

|  |
| --- |
|  |

|  |
| --- |
| Examples Click here for an example. |
