# Supplementary material for: The Systems Biology Research Tool: evolvable open-source software
Source: BMC Syst Biol. 2008 Jun 29;2:55. doi: 10.1186/1752-0509-2-55 (PMC2446383; doi:10.1186/1752-0509-2-55)
Supplement: Additional file 1 — SBRT Archive. An archive of the current version of the Systems Biology Research Tool. [file 1752-0509-2-55-S1.zip › sbrt-1.4.0/doc/users_guide/fba/files/Flux_Cap_Files.html]

Flux Cap Files - Systems Biology Research Tool


|  |
| --- |
| > User's Guide > Flux Balance Analysis |
|  |
| Flux Cap Files Flux cap files are a type of single-vector file. The *variables* in these files are reaction names, and the *values* are sets of flux caps for the corresponding reaction. Each flux cap must be formatted as a linear combination of reactions names. Each set of flux caps must be formatted in the same way that sets are formatted in set files.  The Flux Cap Identification process can be used to generate flux cap files for a particular stoichiometric network.  See FBA Reaction Files for more information about reaction names.   See the Text Formatting Rules for additional information. |
