# Supplementary material for: The Systems Biology Research Tool: evolvable open-source software
Source: BMC Syst Biol. 2008 Jun 29;2:55. doi: 10.1186/1752-0509-2-55 (PMC2446383; doi:10.1186/1752-0509-2-55)
Supplement: Additional file 1 — SBRT Archive. An archive of the current version of the Systems Biology Research Tool. [file 1752-0509-2-55-S1.zip › sbrt-1.4.0/doc/users_guide/fba/files/Constraints_Files.html]

Constraints Files - Systems Biology Research Tool


|  |
| --- |
| > User's Guide > Flux Balance Analysis |
|  |
| Constraints Files A constraints file is a single-vector file containing user-defined flux constraints. The *variables* in these files can be either reaction names or  linear combinations of reaction names. The *values* in these files are  intervals that are used to define the lower and upper flux bounds of the specified variable.  See FBA Reaction Files for more information about reaction names and the default flux constraints.  See the Text Formatting Rules for additional information. |
