# Supplementary material for: The Systems Biology Research Tool: evolvable open-source software
Source: BMC Syst Biol. 2008 Jun 29;2:55. doi: 10.1186/1752-0509-2-55 (PMC2446383; doi:10.1186/1752-0509-2-55)
Supplement: Additional file 1 — SBRT Archive. An archive of the current version of the Systems Biology Research Tool. [file 1752-0509-2-55-S1.zip › sbrt-1.4.0/doc/users_guide/fba/files/Reaction-Catalyst_Association_Files.html]

Reaction-Catalyst Association Files - Systems Biology
Research Tool


|  |
| --- |
| > User's Guide > Flux Balance Analysis |
|  |
| Reaction-Catalyst Association Files A *reaction-catalyst association file* is a type of single-vector file used to store boolean logic relationships between reactions and catalysts. The *variables* in these files are reaction names, and the *values* are groups of catalysts that are co-required for the reaction to function. A group of co-required catalysts must be delimited by the ampersand character "&", and independent groups of catalysts must be delimited by the pipe character "|". Any whitespace characters around the ampersand and pipe characters are ignored. The names of catalysts cannot contain special characters.  See FBA Reaction Files for more information about reaction names.   See the Text Formatting Rules for additional information. Example 1 Let R denote a chemical reaction that can be catalyzed by three enzyme complexes. For these complexes to function as catalysts for R, all of their constituent enzymes must be present. Let complex C1 be composed of enzymes E1 and E2; let complex C2 be composed of enzymes E1 and E3; and let complex C3 be composed of enzymes E2 and E4. This relationship could be represented in a catalyst file as: R = E1 & E2 | E1 & E3 | E2 & E4 This relationship could be read as: R can function if E1 and E2 are present, or if E1 and E3 are present, or if E2 and E4 are present. Example 2 Let R denote a chemical reaction that can be catalyzed by three enzymes E1, E2, and E3 independently. That is, R can function if any one of the three enzymes is present. This relationship could be represented in a catalyst file as: R = E1 | E2 | E3 This relationship could be read as: R can function if E1 is present, or if E2 is present, or if E3 is present. Example 3 Let R denote a chemical reaction that can be catalyzed by a single enzyme complex containing three enzymes E1, E2, and E3. For this complex to function as a catalyst for R, all three enzymes must be present. This relationship could be represented in a catalyst file as: R = E1 & E2 & E3 This relationship could be read as: R can function if (and only if) E1, E2, and E3 are present. |
