# Supplementary material for: The Systems Biology Research Tool: evolvable open-source software
Source: BMC Syst Biol. 2008 Jun 29;2:55. doi: 10.1186/1752-0509-2-55 (PMC2446383; doi:10.1186/1752-0509-2-55)
Supplement: Additional file 1 — SBRT Archive. An archive of the current version of the Systems Biology Research Tool. [file 1752-0509-2-55-S1.zip › sbrt-1.4.0/doc/users_guide/fba/files/Cv_Files.html]

Constraint Variation Files - Systems Biology Research
Tool


|  |
| --- |
| > User's Guide > Flux Balance Analysis |
|  |
| Constraint Variation Files Constraint variation files are a type of multiple-vectors file. The *variables* in these files can be either reaction names or linear combinations of reaction names. The *values* in these files are  intervals that are used to define the lower and upper flux bounds of the specified variable. In other words, each line in this type of file contains a set of flux constraints.  See FBA Reaction Files  for more information about reaction names and the default flux constraints.   See the Text Formatting Rules for additional information. |
