# Supplementary material for: The Systems Biology Research Tool: evolvable open-source software
Source: BMC Syst Biol. 2008 Jun 29;2:55. doi: 10.1186/1752-0509-2-55 (PMC2446383; doi:10.1186/1752-0509-2-55)
Supplement: Additional file 1 — SBRT Archive. An archive of the current version of the Systems Biology Research Tool. [file 1752-0509-2-55-S1.zip › sbrt-1.4.0/doc/users_guide/fba/files/FBA_Single_Opt_Output_Files.html]

FBA Single-Optimization Output Files -
Systems Biology Research Tool


|  |
| --- |
| > User's Guide > Flux Balance Analysis |
|  |
| FBA Single-Optimization Output Files These files are a type of  single-vector file that are used to store the results of a single FBA optimization. The *variables* in these files can be reaction names or the string "Objective\_Value". The *values* in these files are the values computed by the linear program solver. The values corresponding to reaction names are fluxes, and the value corresponding to Objective\_Value is the optimal value of the specified objective function.  See FBA Reaction Files for more information about reaction names.  See the Text Formatting Rules for additional information. |
