# Supplementary material for: The Systems Biology Research Tool: evolvable open-source software
Source: BMC Syst Biol. 2008 Jun 29;2:55. doi: 10.1186/1752-0509-2-55 (PMC2446383; doi:10.1186/1752-0509-2-55)
Supplement: Additional file 1 — SBRT Archive. An archive of the current version of the Systems Biology Research Tool. [file 1752-0509-2-55-S1.zip › sbrt-1.4.0/doc/users_guide/fba/files/Obj_Function_Files.html]

Objective Function Files - Systems Biology
Research Tool


|  |
| --- |
| > User's Guide > Flux Balance Analysis |
|  |
| Objective Function Files An objective function file contains a list of FBA objective funtions, one per line. Objective function files can be either text files or gzipped text files. The default file format is Text. See File Formats  for more information.  See the Text Formatting Rules for additional information. |
