# Supplementary material for: The Systems Biology Research Tool: evolvable open-source software
Source: BMC Syst Biol. 2008 Jun 29;2:55. doi: 10.1186/1752-0509-2-55 (PMC2446383; doi:10.1186/1752-0509-2-55)
Supplement: Additional file 1 — SBRT Archive. An archive of the current version of the Systems Biology Research Tool. [file 1752-0509-2-55-S1.zip › sbrt-1.4.0/doc/users_guide/fba/files/Reaction_Files.html]

FBA Reaction Files - Systems Biology Research Tool


|  |
| --- |
| > User's Guide > Flux Balance Analysis |
|  |
| FBA Reaction Files An *FBA reaction file* is a type of single-vector file used to store all of the chemical reactions in a stoichiometric network. The *variables* in these files are the names (or ID's) of the reactions, and the *values* are the chemical reactions themselves. The syntax of a line is: *Name* = *Reactants* --> *Products*. The reactants and products are  linear combinations of chemical species; they cannot contain any chemical species in common; a chemical species cannot occur more than once on either side of the arrow; the stoichiometric coefficients must be parsable as positive, finite, double precision numbers; and a constant is not allowed. All reactions in the file are considered to be irreversible, and the arrow "-->" must be used to denote this. The names of reactions and chemical species cannot contain whitespace or special characters. Chemical species cannot be named "Surroundings" (see below).  Every reaction in these files is used to create a corresponding column in a stoichiometry matrix *S*. These files should contain all internal and exchange reactions intended to exist in *S*.  Since all reactions in these files are considered to be irreversible, exchange reactions are not bidirectional. Source (or input) reactions are used to transport chemical species from the surroundings, across the system boundary, into the system. Sink (or output) reactions are used to transport chemical species from the system, across the system boundary, into the surroundings.  Source reactions are specified using the following syntax: *Name* = Surroundings --> *Species*. Sink reactions are specified using the following syntax: *Name* = *Species* --> Surroundings. The string "Surroundings" is used literally, and it must appear by itself. Only a single chemical species can occur for each exchange reaction. By default, the fluxes of source reactions are constrained to the interval [0, 0], and the fluxes of internal and sink reactions are constrained to the interval [0, ∞).  See the FBA Utilities for information about creating and extracting information from these files. See the Text Formatting Rules as well. |

  
  
