# Supplementary material for: The Systems Biology Research Tool: evolvable open-source software
Source: BMC Syst Biol. 2008 Jun 29;2:55. doi: 10.1186/1752-0509-2-55 (PMC2446383; doi:10.1186/1752-0509-2-55)
Supplement: Additional file 1 — SBRT Archive. An archive of the current version of the Systems Biology Research Tool. [file 1752-0509-2-55-S1.zip › sbrt-1.4.0/doc/users_guide/fba/files/Equivalent_Reaction_Files.html]

Equivalent Reaction Files - Systems Biology Research Tool


|  |
| --- |
| > User's Guide > Flux Balance Analysis |
|  |
| Equivalent Reaction Files Equivalent reaction files are used to store the names of stoichiometrically equivalent reactions. An equivalent reaction file is a type of reaction name file, where each set of reaction names is composed of the names of stoichiometrically equivalent reactions.  See FBA Reaction Files for more information about reaction names.   See the Text Formatting Rules for additional information. |
