# Supplementary material for: The Systems Biology Research Tool: evolvable open-source software
Source: BMC Syst Biol. 2008 Jun 29;2:55. doi: 10.1186/1752-0509-2-55 (PMC2446383; doi:10.1186/1752-0509-2-55)
Supplement: Additional file 1 — SBRT Archive. An archive of the current version of the Systems Biology Research Tool. [file 1752-0509-2-55-S1.zip › sbrt-1.4.0/doc/users_guide/fba/files/Multiple_Flux_Interval_Vectors_Files.html]

Multiple-Flux Interval Vectors Files - Systems Biology
Research Tool


|  |
| --- |
| > User's Guide > Flux Balance Analysis |
|  |
| Multiple-Flux Interval Vectors Files Multiple-flux interval vectors files are a type of multiple-vectors file. The *variables* in these files are reaction names, and the *values* are intervals that represent the lower and upper flux bounds of the corresponding reaction. These intervals are not the intervals in which the fluxes are *constrained* to lie, but rather, they're the intervals in which the fluxes actually *do* lie.  See FBA Reaction Files for more information about reaction names.   See the Text Formatting Rules for additional information. |
