# Supplementary material for: The Systems Biology Research Tool: evolvable open-source software
Source: BMC Syst Biol. 2008 Jun 29;2:55. doi: 10.1186/1752-0509-2-55 (PMC2446383; doi:10.1186/1752-0509-2-55)
Supplement: Additional file 1 — SBRT Archive. An archive of the current version of the Systems Biology Research Tool. [file 1752-0509-2-55-S1.zip › sbrt-1.4.0/doc/users_guide/fba/index.html]

Flux Balance Analysis - Systems Biology Research Tool


|  |
| --- |
| > User's Guide |
|  |
| Flux Balance Analysis |
|  |
| The term *flux balance analysis* is used here to refer to the study of the equation *Sv = 0*, where *S* denotes a stoichiometry matrix and *v* denotes a vector of fluxes. |

  
  


|  |  |
| --- | --- |
| Process Categories | Brief Descriptions |
| Optimization | Used to compute the optimal values of objective functions in various ways. |
| Flux Variability | Used to compute the variability of fluxes in various ways. |
| Pathway Identification | Used to identify metabolic pathways. |
| Flux Space Sampling | Used to create and sample flux spaces in various ways. |
| Data Analysis | Used to analyze various FBA data sets. |
| Utilities | Used to perform useful tasks. |
|  |
| Definitions | Brief Descriptions |
| Flux Space | The set of flux vectors that satisfy all of the constraints defined for the system *Sv = 0*. |
| Stoichiometric Equivalence | Equivalent representations of chemical reactions. |
|  |
| Files | Brief Descriptions |
| FBA Reaction Files | Used to store all of the reactions of a stoichiometric network. |
| Reaction-Catalyst Association Files | Used to store reaction-catalyst associations for a stoichiometric network. |
| Constraints Files | Used to store a single set of flux constraints. |
| Constraint Variation Files | Used to store multiple sets of flux constraints. |
| FBA Single-Optimization Output Files | Used to store the results of a single optimization. |
| FBA Multiple-Optimizations Output Files | Used to store the results of multiple optimizations. |
| Objective Function Files | Used to store a list of objective functions. |
| Catalyst Name Files | Used to store sets of catalyst names. |
| Reaction Name Files | Used to store sets of reaction names. |
| Equivalent Reaction Files | Used to store the names of stoichiometrically equivalent reactions. |
| Single-Flux Vector Files | Used to store a single flux vector. |
| Multiple-Flux Vectors Files | Used to store multiple flux vectors. |
| Single-Flux Interval Vector Files | Used to store a single vector of flux intervals. |
| Multiple-Flux Interval Vectors Files | Used to store multiple vectors of flux intervals. |
|  |
| Miscellaneous | Brief Descriptions |
| FBA Objective Functions | The objective functions used in Flux Balance Analysis. |
| Reaction Name Data Headers | The data headers of output files used by many FBA processes. |
| FBA Optimization Data Headers | The data headers of FBA optimization output files. |
| Safety Levels | Used for error checking. |
| Constraint Tolerances | Used for error checking. |
| Zero Cutoffs | Used for error checking. |
| Pseudo-Random Number Generator Seeds | Used for generating pseudo-random numbers. |

  
  
