# Supplementary material for: The Systems Biology Research Tool: evolvable open-source software
Source: BMC Syst Biol. 2008 Jun 29;2:55. doi: 10.1186/1752-0509-2-55 (PMC2446383; doi:10.1186/1752-0509-2-55)
Supplement: Additional file 1 — SBRT Archive. An archive of the current version of the Systems Biology Research Tool. [file 1752-0509-2-55-S1.zip › sbrt-1.4.0/doc/users_guide/fba/algorithms/Flux_Caps.html]

Flux Caps - Systems Biology Research Tool


|  |
| --- |
| > User's Guide > Flux Balance Analysis > Flux Variability |
|  |
| Flux CapsIntroduction In the typical formulation of a flux balance analysis (FBA) problem, the fluxes of the internal chemical reactions are constrained to the interval [0, ∞). This does not mean that internal fluxes are unbounded, but rather, the true upper bound is not known *a priori*. Using the constraint [0, ∞) simply ensures that the flux space is not artificially restricted.  If the exchange reactions of a stoichiometric network are constrained to be finite, then for fundamental thermodynamic reasons, the flux of each internal reaction should be finite as well [1]. This is not always the case in the typical FBA formulation: the fluxes of reactions participating in steady state cycles (type III extreme pathways) are unbounded in the positive direction [2]. The application of *flux caps* is one way to prohibit these physically unrealistic unbounded fluxes. What are flux caps? Flux caps are linear combinations of fluxes used to bound other fluxes. They contain a single flux from each cycle in which an unbounded reaction participates. By constraining the value of a flux cap, an unbounded flux will become "capped". This will be illustrated for the stoichiometric network below.   |  |  |  |  |  |  |  |  |  |  |  |  |  |  |  |  | | --- | --- | --- | --- | --- | --- | --- | --- | --- | --- | --- | --- | --- | --- | --- | --- | |  | |  |  | | --- | --- | | **Flux** | **Constraint** | | V1 | [0, ∞) | | V2 | [0, ∞) | | V3 | [0, ∞) | | V4 | [0, ∞) | | V5 | [0, 10] | | V6 | [4, 6] | |   The dotted line indicates the system boundary, and the table on the right denotes the defined flux constraints. The reactions corresponding to fluxes V1-4 are internal, and V5 and V6 represent the fluxes of exchange reactions. This stoichiometric network contains two cycles, V1 + V2 + V4 and V1 + V3, causing each of the internal fluxes to be unbounded. Flux caps can be determined for each flux of this stoichiometric network by visual inspection.   |  |  | | --- | --- | | **Flux** | **Flux Cap** | | V1 | V2 + V3, V4 + V3 | | V2 | V1, V4 | | V3 | V1 | | V4 | V1, V2 |   Notice that flux caps are minimal in the sense that they only contain a single flux from each cycle in which their corresponding reaction participates. Also notice that multiple flux caps can exist for a single reaction. If this stoichiometric network had contained "redundant" reactions (i.e. multiple reactions that are stoichiometrically equivalent), they could appear together in the same flux caps as well. In other words, it is unnecessary to create separate flux caps for redundant reactions. An algorithm for determining all the possible flux caps in a stoichiometric network is described below. Identifying all possible flux caps Stoichiometric networks often contain stoichiometrically equivalent (i.e. "redundant") reactions. Let *ei* denote any particular set of stoichiometrically equivalent reactions in a network *S*. Let *E* denote the collection of all sets *ei* in *S*. Designate an arbitrary reaction from each set *ei* in *E* as "primary" and the other reactions as "secondary". The following steps are executed for each reaction *R* that participates in a cycle:   |  |  | | --- | --- | | 1. | Let *F* denote the set of all cycles in which *R* participates. | | 2. | Let *L* denote a list of sets, where each set contains the reactions of each cycle in *F*. | | 3. | For each set in *L*, remove *R* and any reactions that are stoichiometrically equivalent to *R*. | | 4. | Remove from *L* any sets that contain "secondary" reactions. | | 5. | Compute the single-element unions of sets in *L*, and let *U* denote this collection of single-element unions. | | 6. | To the sets in *U*, add all reactions from the stoichiometric network that are stoichiometrically equivalent to the reactions they contain. | | 7. | Remove from *U* any set that contains more than one reaction from any single cycle in *F*. | | 8. | Convert the sets of reactions in *U* to linear combinations of their corresponding fluxes. *U* now contains each flux cap for *R*. |  Using flux caps Flux caps are used to compute Mahadevan-Schilling flux intervals. This will be illustrated for the stoichiometric network and flux caps described above. The first step is to determine finite upper bounds for each of the reactions participating in cycles.  **For V1:**   1. Compute the minimum value of V2 + V3,    which is 4. 2. Apply the constraint V2 + V3 = 4. 3. Compute the maximum value of V1, which is 4. 4. Restore the original constraint on V2 + V3,    that is, -∞ < V2 + V3 < ∞. 5. Compute the minimum value of V4 + V3,    which is 0. 6. Apply the constraint V4 + V3 = 0. 7. Compute the maximum value of V1, which is 6. 8. Restore the original constraint on V4 + V3,    that is, -∞ < V4 + V3 < ∞. 9. Since the value computed in Step 7 is greater than the one    computed in Step 3, it will be chosen as the upper bound for V1.   **For V3:**   1. Compute the minimum value of V1, which is 4. 2. Apply the constraint V1 = 4. 3. Compute the maximum value of V3, which is 0. 4. Restore the original constraint on V1, that is, 0    ≤ V1 < ∞. 5. The value computed in Step 3 is chosen as the upper bound    for V3.   The upper bounds for V2 and V4 are computed in similar ways, resulting in the following values.   |  |  |  |  |  | | --- | --- | --- | --- | --- | | **Flux** | V1 | V2 | V3 | V4 | | **Upper Bound** | 6 | 6 | 0 | 0 |   These values are used to define a new set of flux constraints:   |  |  | | --- | --- | | **Flux** | **Constraint** | | V1 | [0, 6] | | V2 | [0, 6] | | V3 | [0, 0] | | V4 | [0, 0] | | V5 | [0, 10] | | V6 | [4, 6] |   Now that a finite upper bound is defined for each flux, the Mahadevan-Schilling flux intervals can be computed. Each flux is individually minimized and maximized, resulting in the following:   |  |  | | --- | --- | | **Flux** | **Interval** | | V1 | [4, 6] | | V2 | [4, 6] | | V3 | [0, 0] | | V4 | [0, 0] | | V5 | [4, 6] | | V6 | [4, 6] |  References  |  |  | | --- | --- | | 1. | Beard D. A., Liang S. D., Qian H. (2002). *Energy balance for analysis of complex metabolic networks.* Biophys. J., 83: 79-86. | | 2. | Mahadevan R., Schilling C. H. (2003) *The effects of alternate optimal solutions in constraint-based genome-scale metabolic models.* Metab. Eng., 5(4): 264-76. | |
