# Supplementary material for: The Systems Biology Research Tool: evolvable open-source software
Source: BMC Syst Biol. 2008 Jun 29;2:55. doi: 10.1186/1752-0509-2-55 (PMC2446383; doi:10.1186/1752-0509-2-55)
Supplement: Additional file 1 — SBRT Archive. An archive of the current version of the Systems Biology Research Tool. [file 1752-0509-2-55-S1.zip › sbrt-1.4.0/doc/users_guide/fba/misc/Constraint_Tolerances.html]

Constraint Tolerances - Systems Biology Research Tool


|  |
| --- |
| > User's Guide > Flux Balance Analysis |
|  |
| Constraint Tolerances A constraint tolerance is the amount by which a linear program solver is allowed to violate the defined flux constraints. Constraint tolerances must be positive, finite, double precision numbers. They should also be close to zero, like 1E-6 for example. Constraint tolerances work in conjunction with  Safety Levels.  The default constraint tolerance is 1E-9. |
