# Supplementary material for: The Systems Biology Research Tool: evolvable open-source software
Source: BMC Syst Biol. 2008 Jun 29;2:55. doi: 10.1186/1752-0509-2-55 (PMC2446383; doi:10.1186/1752-0509-2-55)
Supplement: Additional file 1 — SBRT Archive. An archive of the current version of the Systems Biology Research Tool. [file 1752-0509-2-55-S1.zip › sbrt-1.4.0/doc/users_guide/fba/misc/Safety_Levels.html]

Safety Levels - Systems Biology Research Tool


|  |
| --- |
| > User's Guide > Flux Balance Analysis |
|  |
| Safety Levels Saftey levels are used to detect mistakes made by linear program solvers.  A safety level of 0 indicates that no checking will be performed. In other words, the results computed by the linear program solver are assumed to be completely trustworthy.  A safety level of 1 indicates that the output from the linear program solver will be checked for flux constraint violations. If the linear program solver returns a flux value that lies outside of its defined constraint by more than the specified constraint tolerance, the program will terminate with an error message.  The default safety level is 1. |
