# Supplementary material for: The Systems Biology Research Tool: evolvable open-source software
Source: BMC Syst Biol. 2008 Jun 29;2:55. doi: 10.1186/1752-0509-2-55 (PMC2446383; doi:10.1186/1752-0509-2-55)
Supplement: Additional file 1 — SBRT Archive. An archive of the current version of the Systems Biology Research Tool. [file 1752-0509-2-55-S1.zip › sbrt-1.4.0/doc/users_guide/fba/misc/Zero_Cutoffs.html]

Zero Cutoffs - Systems Biology Research Tool


|  |
| --- |
| > User's Guide > Flux Balance Analysis |
|  |
| Zero Cutoffs A zero cutoff is the amount by which a computed value can differ from zero, but still be considered equal to zero. Zero cutoffs must be positive, finite, double precision numbers. They should also be close to zero, like 1E-6 for example. Choosing a suitable value should be based on the expected numerical error of the corresponding computation.  The default zero cutoff is 0.0d, that is, zero. |
