# Supplementary material for: The Systems Biology Research Tool: evolvable open-source software
Source: BMC Syst Biol. 2008 Jun 29;2:55. doi: 10.1186/1752-0509-2-55 (PMC2446383; doi:10.1186/1752-0509-2-55)
Supplement: Additional file 1 — SBRT Archive. An archive of the current version of the Systems Biology Research Tool. [file 1752-0509-2-55-S1.zip › sbrt-1.4.0/doc/users_guide/fba/misc/FBA_Objective_Functions.html]

Objective Functions - Systems Biology Research Tool


|  |
| --- |
| > User's Guide > Flux Balance Analysis |
|  |
| FBA Objective Functions An objective function is a mathematical expression whose optimal value is computed. The Systems Biology Research Tool currently supports the use of two types of objective functions in flux balance analysis. Objective functions can be single reaction names (indicating that the flux of the corresponding reaction should be optimized) or linear combinations of reaction names (indicating that the linear combination of fluxes of the corresponding reactions should be optimized).  See FBA Reaction Files for more information about reaction names. |
