# Supplementary material for: The Systems Biology Research Tool: evolvable open-source software
Source: BMC Syst Biol. 2008 Jun 29;2:55. doi: 10.1186/1752-0509-2-55 (PMC2446383; doi:10.1186/1752-0509-2-55)
Supplement: Additional file 1 — SBRT Archive. An archive of the current version of the Systems Biology Research Tool. [file 1752-0509-2-55-S1.zip › sbrt-1.4.0/doc/users_guide/fba/misc/FBA_Opt_Data_Headers.html]

FBA Optimization Data Headers - Systems
Biology Research Tool


|  |
| --- |
| > User's Guide > Flux Balance Analysis |
|  |
| FBA Optimization Data Headers FBA optimization data headers indicate which values will be written to FBA optimization output files. Data headers can be reaction names or the string "Objective\_Value". The string "All\_Reactions" can also be used as a short-hand notation to indicate that all reaction names should appear as data headers. Data headers are represented as a pipe-delimited set on a single line with the syntax: Header\_1 | Header\_2 | ... | Header\_N See FBA Reaction Files for more information about reaction names.  See FBA Single-Optimization Output Files and FBA Multiple-Optimization Output Files for more information about FBA optimization output files. |
