# Supplementary material for: The Systems Biology Research Tool: evolvable open-source software
Source: BMC Syst Biol. 2008 Jun 29;2:55. doi: 10.1186/1752-0509-2-55 (PMC2446383; doi:10.1186/1752-0509-2-55)
Supplement: Additional file 1 — SBRT Archive. An archive of the current version of the Systems Biology Research Tool. [file 1752-0509-2-55-S1.zip › sbrt-1.4.0/doc/users_guide/fba/misc/Seeds.html]

PRNG Seeds - Systems Biology Research Tool


|  |
| --- |
| > User's Guide > Flux Balance Analysis |
|  |
| Pseudo-Random Number Generator Seeds Psuedo-random number generators (PRNG) require a *seed*. Seeds must be whole numbers, specifically of type long.  See the Java Language Specification and java.lang.Long.parseLong(java.lang.String) for more information about long numbers and how they are parsed.  See java.util.Random for more information about the pseudo-random number generator used by the Systems Biology Research Tool. |
