# Supplementary material for: The Systems Biology Research Tool: evolvable open-source software
Source: BMC Syst Biol. 2008 Jun 29;2:55. doi: 10.1186/1752-0509-2-55 (PMC2446383; doi:10.1186/1752-0509-2-55)
Supplement: Additional file 1 — SBRT Archive. An archive of the current version of the Systems Biology Research Tool. [file 1752-0509-2-55-S1.zip › sbrt-1.4.0/doc/users_guide/fba/misc/Reaction_Name_Data_Headers.html]

Reaction Name Data Headers - Systems Biology Research
Tool


|  |
| --- |
| > User's Guide > Flux Balance Analysis |
|  |
| Reaction Name Data Headers Data headers indicate which values will be written to output files. These headers must be either reaction names from the provided stoichiometric network, or the string "All\_Reactions" can also be used as a short-hand notation to indicate that all reaction names should appear as data headers. Data headers are represented as a pipe-delimited set on a single line with the syntax: Header\_1 | Header\_2 | ... | Header\_N Whitespace characters around the pipes "|" are ignored.  See FBA Reaction Files for more information about reaction names. |
