# Supplementary material for: The Systems Biology Research Tool: evolvable open-source software
Source: BMC Syst Biol. 2008 Jun 29;2:55. doi: 10.1186/1752-0509-2-55 (PMC2446383; doi:10.1186/1752-0509-2-55)
Supplement: Additional file 1 — SBRT Archive. An archive of the current version of the Systems Biology Research Tool. [file 1752-0509-2-55-S1.zip › sbrt-1.4.0/doc/users_guide/getting_started/Processes.html]

Processes - Systems Biology Research Tool


|  |
| --- |
| > User's Guide |
|  |
| Processes  The Systems Biology Research Tool is designed to execute *processes*. A process is simply a systematic series of actions that produce a result. Algorithms, procedures, analyses, and computational experiments are all examples of *processes*. Each process executed by the Systems Biology Research Tool requires some form of user-supplied input and produces some form of output. Processes are referred to by their names, which are defined in Process Name Files. |
