# Supplementary material for: The Systems Biology Research Tool: evolvable open-source software
Source: BMC Syst Biol. 2008 Jun 29;2:55. doi: 10.1186/1752-0509-2-55 (PMC2446383; doi:10.1186/1752-0509-2-55)
Supplement: Additional file 1 — SBRT Archive. An archive of the current version of the Systems Biology Research Tool. [file 1752-0509-2-55-S1.zip › sbrt-1.4.0/doc/users_guide/getting_started/GUI.html]

The GUI - Systems Biology Research Tool


|  |
| --- |
| > User's Guide |
|  |
| The Graphical User Interface (GUI)   Launching the GUI The Systems Biology Research Tool's GUI can be invoked from a terminal by issuing the command: **sbrt** -g Windows users can also launch the GUI by clicking:  **Start → Programs → Systems Biology Research Tool → Systems Biology Research Tool.exe**.  A window similar to the following should appear.  The Java Virtual Machine takes some time to initialize, so a delay may be noticed when the GUI is first launched. This is common for applications written in Java. Using the GUI The "**Select a working directory**" button allows the user to specifiy the Working Directory.  The "**Execute an SBRT process**" button allows the user to specifiy a Process File. |
