# Supplementary material for: The Systems Biology Research Tool: evolvable open-source software
Source: BMC Syst Biol. 2008 Jun 29;2:55. doi: 10.1186/1752-0509-2-55 (PMC2446383; doi:10.1186/1752-0509-2-55)
Supplement: Additional file 1 — SBRT Archive. An archive of the current version of the Systems Biology Research Tool. [file 1752-0509-2-55-S1.zip › sbrt-1.4.0/doc/users_guide/getting_started/JVM.html]

JVM - Systems Biology Research Tool


|  |
| --- |
| > User's Guide |
|  |
| The Java Virtual Machine    The Systems Biology Research Tool is compatible with Java Virtual Machines (version 1.5 and later) from Sun Microsystems. JVMs are included in the SBRT installation packages, and separate installation is not necessary. The file sbrt.vmoptions in the SBRT's bin directory is used to supply options to the JVM. A single option must appear on each line, and the name of this file should not be changed. See the list of Java Hotspot VM Options from Sun for additional information. |
