# Supplementary material for: The Systems Biology Research Tool: evolvable open-source software
Source: BMC Syst Biol. 2008 Jun 29;2:55. doi: 10.1186/1752-0509-2-55 (PMC2446383; doi:10.1186/1752-0509-2-55)
Supplement: Additional file 1 — SBRT Archive. An archive of the current version of the Systems Biology Research Tool. [file 1752-0509-2-55-S1.zip › sbrt-1.4.0/doc/users_guide/getting_started/Working_Dir.html]

Working Directory - Systems Biology Research Tool


|  |
| --- |
| > User's Guide |
|  |
| The Working Directory    The Systems Biology Research Tool performs certain actions in a directory of the file system referred to as the *working directory*. If the SBRT produces an error log, it will be written to the working directory. When file names are supplied to the SBRT, they can be specified relative to the working directory.  If the program **sbrt** is issued from the command line, the working directory is the directory of the user when the command was executed. The working directory can be specified manually when using the GUI. |
