# Supplementary material for: The Systems Biology Research Tool: evolvable open-source software
Source: BMC Syst Biol. 2008 Jun 29;2:55. doi: 10.1186/1752-0509-2-55 (PMC2446383; doi:10.1186/1752-0509-2-55)
Supplement: Additional file 1 — SBRT Archive. An archive of the current version of the Systems Biology Research Tool. [file 1752-0509-2-55-S1.zip › sbrt-1.4.0/doc/users_guide/getting_started/Command_Line.html]

The Command Line - Systems Biology Research Tool


|  |
| --- |
| > User's Guide |
|  |
| The Command Line  The Systems Biology Research Tool can be executed from a terminal by issuing the following command: **sbrt** arguments The argument **-g** is used to launch the Systems Biology Research Tool's GUI. If this argument is supplied, no other arguments can be given.  The argument **-v** is used to display the version number of the Systems Biology Research Tool. If this argument is supplied, no other arguments can be given.  If a single argument is provided other than **-g** or **-v**, it must be the name of a Process File.  If multiple arguments are provided, they must constitute a set of keyword-value pairs. The syntax of a single pair is: --Keyword=Value. The allowable keyword-value pairs are specific to each process of the Systems Biology Research Tool. The pair must be supplied to the program as a single argument, and the way to achieve this depends on the operating system. Typically, whitespace characters are not allowed in single command line arguments. If a process defines a keyword that contains spaces, they can each be substituted with an underscore character "\_". If a value must contain spaces, surrounding it with double quotes (i.e. "Value\_Part\_1 Value\_Part\_2") is likely to succeed. |
