# Supplementary material for: The Systems Biology Research Tool: evolvable open-source software
Source: BMC Syst Biol. 2008 Jun 29;2:55. doi: 10.1186/1752-0509-2-55 (PMC2446383; doi:10.1186/1752-0509-2-55)
Supplement: Additional file 1 — SBRT Archive. An archive of the current version of the Systems Biology Research Tool. [file 1752-0509-2-55-S1.zip › sbrt-1.4.0/doc/users_guide/graph_theory/processes/Unique_Cycle_Id.html]

Unique Cycle Identification - Systems Biology Research
Tool


|  |
| --- |
| > User's Guide > Graph Theory |
|  |
| Unique Cycle Identification This process is used to identify, or enumerate, all of the *unique* cycles in a collection of simple cycles. A simple cycle is considered to be a sequence of vertices, connected by edges, in which no vertex is repeated except for the start and end points. Given this definition, a single cycle may be represented in various ways, depending on the choice of the starting node. This process interprets these potentially differing representations, and reports only the truly unique cycles.  The input to this process must be a text file containing a collection of cycles, such as that produced by the Cycle Identification process. The total number of unique cycles are written to stdout, and the cycles themselves are written to an output file.  Here is the set of keywords this process understands, along with a description of their possible corresponding values. |

  


|  |  |
| --- | --- |
| Required Keywords | Possible Values |
| Process Name File | The name of the file where process names are defined. See  Process Name Files for further information. |
| Process | The name defined in the specified process name file.  Unique Cycle Identification is the default value. |
| Input File | The name of a text file containing a collection of simple cycles. See Path Files for further information. |
|  |
| Optional Keywords | Possible Values |
| Input File Name Format | Either Text or Gzipped Text, depending on the format of the input file. See File Formats for additional information. |
| Output File Name | The name of the file to be created by this process. See Path Files for further information. |
| Output File Name Format | Either Text or Gzipped Text. See File Formats for additional information. |

|  |
| --- |
|  |

|  |
| --- |
| Examples Click here for an example. |
