# Supplementary material for: The Systems Biology Research Tool: evolvable open-source software
Source: BMC Syst Biol. 2008 Jun 29;2:55. doi: 10.1186/1752-0509-2-55 (PMC2446383; doi:10.1186/1752-0509-2-55)
Supplement: Additional file 1 — SBRT Archive. An archive of the current version of the Systems Biology Research Tool. [file 1752-0509-2-55-S1.zip › sbrt-1.4.0/doc/users_guide/graph_theory/processes/Path_Id_Dir_Graph.html]

Path Identification - Systems Biology Research Tool


|  |
| --- |
| > User's Guide > Graph Theory |
|  |
| Path Identification in a Directed Graph This process is used to identify, or enumerate, all simple paths in a directed graph. A directed graph is one in which the edges, or links, are directed from one vertex, or node, to another. A simple path is a sequence of vertices, connected by edges, in which no vertex is repeated. This process will write the total number of computed paths to stdout, and if an output file name is provided, the paths will be written to it.  See MathWorld's description of  directed graphs, and Wikipedia's description of  directed graphs and  simple paths for additional information.  The algorithm used by this process was described in: Carré, Bernard. Graphs and Networks. Clarendon Press, 1979. pg. 65-71.  Here is the set of keywords this process understands, along with a description of their possible corresponding values. See the command line documentation for more information about keyword-value pairs. |

  


|  |  |
| --- | --- |
| Required Keywords | Possible Values |
| Process Name File | The name of the file where process names are defined. See  Process Name Files for further information. |
| Process | The name defined in the specified process name file.  Path Identification is the default value. |
| Edge File | The name of a text file containing the edges of a directed graph. See Edge Files for further information. |
|  |
| Optional Keywords | Possible Values |
| Output File Name | The name of the file to be created by this process. See Path Files for further information. |
| Output File Name Format | Either Text or Gzipped Text. See File Formats for additional information. |

|  |
| --- |
|  |

|  |
| --- |
| Examples Click here for an example. |
