# Supplementary material for: The Systems Biology Research Tool: evolvable open-source software
Source: BMC Syst Biol. 2008 Jun 29;2:55. doi: 10.1186/1752-0509-2-55 (PMC2446383; doi:10.1186/1752-0509-2-55)
Supplement: Additional file 1 — SBRT Archive. An archive of the current version of the Systems Biology Research Tool. [file 1752-0509-2-55-S1.zip › sbrt-1.4.0/doc/users_guide/graph_theory/processes/Cycle_Id_Dir_Graph.html]

Cycle Identification - Systems Biology Research Tool


|  |
| --- |
| > User's Guide > Graph Theory |
|  |
| Cycle Identification in a Directed Graph This process is used to identify, or enumerate, all of the simple cycles in a directed graph. A directed graph is one in which the edges, or links, are directed from one vertex, or node, to another. A simple cycle is a sequence of vertices, connected by edges, in which no vertex is repeated except for the start and end points. See MathWorld's description of  directed graphs, and Wikipedia's description of  directed graphs and  simple cycles for additional information.  The algorithm used by this process was described in: Carré, Bernard. Graphs and Networks. Clarendon Press, 1979. pg. 65-71; and it identifies all of the cycles in a graph in which a particular vertex participates. To identify *all* of the cycles in a graph, this process applies the algorithm to all vertices in the graph *individually*. Consequently, cycles composed of *n* vertices will be identified and reported *n* times. For an illustration, consider the following graph:  This process will identify and report the following 3 cycles:   |  | | --- | | *A → A* | | *A → B → A* | | *B → A → B* |   Note that the last 2 reported cycles are structurally identical, with the only difference being their vertex of origin. This reporting style may be beneficial for some purposes, and not for others. The Unique Cycle Identification process can be used to eliminate these redundant cycles if necessary.  This process will write the total number of computed cycles to stdout, and if an output file name is provided, the cycles will be written to it.    Here is the set of keywords this process understands, along with a description of their possible corresponding values. |

  


|  |  |
| --- | --- |
| Required Keywords | Possible Values |
| Process Name File | The name of the file where process names are defined. See  Process Name Files for further information. |
| Process | The name defined in the specified process name file.  Cycle Identification is the default value. |
| Edge File | The name of a text file containing the edges of a directed graph. See Edge Files for further information. |
|  |
| Optional Keywords | Possible Values |
| Output File Name | The name of the file to be created by this process. See Path Files for further information. |
| Output File Name Format | Either Text or Gzipped Text. See File Formats for additional information. |

|  |
| --- |
|  |

|  |
| --- |
| Examples Click here for an example. |
