# Supplementary material for: The Systems Biology Research Tool: evolvable open-source software
Source: BMC Syst Biol. 2008 Jun 29;2:55. doi: 10.1186/1752-0509-2-55 (PMC2446383; doi:10.1186/1752-0509-2-55)
Supplement: Additional file 1 — SBRT Archive. An archive of the current version of the Systems Biology Research Tool. [file 1752-0509-2-55-S1.zip › sbrt-1.4.0/doc/users_guide/graph_theory/files/Path_Files.html]

Path Files - Systems Biology Research Tool


|  |
| --- |
| > User's Guide > Graph Theory |
|  |
| Path Files Path files are text files that contain paths from a particular graph. In the most general sense, a path is an ordered list of vertices (or nodes), where the vertex in position *i* is connected to the vertex in position *i+1* by an edge (or link). A single path appears on each line of these files, with the syntax: Vertex\_1 | Vertex\_2 | ... | Vertex\_N Any whitespace characters around the pipes "|" are ignored.  See the Text Formatting Rules for additional information. |
