# Supplementary material for: The Systems Biology Research Tool: evolvable open-source software
Source: BMC Syst Biol. 2008 Jun 29;2:55. doi: 10.1186/1752-0509-2-55 (PMC2446383; doi:10.1186/1752-0509-2-55)
Supplement: Additional file 1 — SBRT Archive. An archive of the current version of the Systems Biology Research Tool. [file 1752-0509-2-55-S1.zip › sbrt-1.4.0/doc/users_guide/graph_theory/files/Edge_Files.html]

Edge Files - Systems Biology Research Tool


|  |
| --- |
| > User's Guide > Graph Theory |
|  |
| Edge Files Edge files are text files that contain the edges of a directed graph, one per line. The syntax is: Source\_Node -> Sink\_Node. The source node is the point from which the edge emanates, and the sink node is the point at which the edge terminates. The strings denoting the nodes can contain any characters except ->. Any whitespace around the arrow is ignored. Self-loops are allowed, but duplicate edges, that is, multigraphs, are not.  See the Text Formatting Rules for additional information. |
