# Supplementary material for: The Systems Biology Research Tool: evolvable open-source software
Source: BMC Syst Biol. 2008 Jun 29;2:55. doi: 10.1186/1752-0509-2-55 (PMC2446383; doi:10.1186/1752-0509-2-55)
Supplement: Additional file 1 — SBRT Archive. An archive of the current version of the Systems Biology Research Tool. [file 1752-0509-2-55-S1.zip › sbrt-1.4.0/doc/users_guide/graph_theory/index.html]

Graph Theoretical Analysis - Systems Biology Research
Tool


|  |
| --- |
| > User's Guide |
|  |
| Graph Theory |

  

|  |  |
| --- | --- |
| Processes | Brief Descriptions |
| Path Identification in a Directed Graph | Used to identify the simple paths in a directed graph. |
| Cycle Identification in a Directed Graph | Used to identify the simple cycles in a directed graph. |
| Unique Cycle Identification | Used to identify the unique cycles in a collection of simple cycles. |
|  |
| Files | Brief Descriptions |
| Edge Files | Used to store the edges of a directed graph. |
| Path Files | Used to store paths of a graph. |
