# Supplementary material for: The Systems Biology Research Tool: evolvable open-source software
Source: BMC Syst Biol. 2008 Jun 29;2:55. doi: 10.1186/1752-0509-2-55 (PMC2446383; doi:10.1186/1752-0509-2-55)
Supplement: Additional file 1 — SBRT Archive. An archive of the current version of the Systems Biology Research Tool. [file 1752-0509-2-55-S1.zip › sbrt-1.4.0/doc/users_guide/files/File_Formats.html]

File Formats - Systems Biology Research Tool


|  |
| --- |
| > User's Guide |
|  |
| File Formats  Text files are the standard file format used by the Systems Biology Research Tool, and they are denoted as Text. Some processes, however, understand other formats, including:   - text files that have been compressed using gzip,   denoted as Gzipped Text; - files containing serialized objects, denoted as Serialized;   and - files containing serialized objects that have been   compressed using gzip, denoted as Gzipped   Serialized.   Files containing serialized objects are not considered to be *text* files.  See http://www.gnu.org/software/gzip/ for more information about gzip.  See http://wikipedia.org/wiki/Serialization ,  http://java.sun.com/j2se/1.4.2/docs/guide/serialization/, and  java.io.Serializable for more information about serialized objects. |
