# Supplementary material for: The Systems Biology Research Tool: evolvable open-source software
Source: BMC Syst Biol. 2008 Jun 29;2:55. doi: 10.1186/1752-0509-2-55 (PMC2446383; doi:10.1186/1752-0509-2-55)
Supplement: Additional file 1 — SBRT Archive. An archive of the current version of the Systems Biology Research Tool. [file 1752-0509-2-55-S1.zip › sbrt-1.4.0/doc/users_guide/files/Process_Files.html]

Process Files - Systems Biology Research Tool


|  |
| --- |
| > User's Guide |
|  |
| Process Files Process files contain the information necessary to execute a process of the Systems Biology Research Tool. Each line contains a single keyword-value pair. The allowable keyword-value pairs are specific to each process. All *required* keywords must be present, and no keyword can appear more than once. The line syntax is: Keyword: Value. Any whitespace characters around the colon are ignored.  See the command line documentation for more information about process files.  See the Text Formatting Rules as well. |
