# Supplementary material for: The Systems Biology Research Tool: evolvable open-source software
Source: BMC Syst Biol. 2008 Jun 29;2:55. doi: 10.1186/1752-0509-2-55 (PMC2446383; doi:10.1186/1752-0509-2-55)
Supplement: Additional file 1 — SBRT Archive. An archive of the current version of the Systems Biology Research Tool. [file 1752-0509-2-55-S1.zip › sbrt-1.4.0/doc/users_guide/files/File_Names.html]

File Names - Systems Biology Research Tool


|  |
| --- |
| > User's Guide |
|  |
| File Names  Files names supplied to the Systems Biology Research Tool can be either absolute or relative to the working directory. See Wikipedia for more information about absolute and relative paths. |
