# Supplementary material for: The Systems Biology Research Tool: evolvable open-source software
Source: BMC Syst Biol. 2008 Jun 29;2:55. doi: 10.1186/1752-0509-2-55 (PMC2446383; doi:10.1186/1752-0509-2-55)
Supplement: Additional file 1 — SBRT Archive. An archive of the current version of the Systems Biology Research Tool. [file 1752-0509-2-55-S1.zip › sbrt-1.4.0/doc/users_guide/files/Special_Characters.html]

Special Characters - Systems Biology Research Tool


|  |
| --- |
| > User's Guide |
|  |
| Special Characters  The following characters are considered to have special meaning to the Systems Biology Research Tool.      |  |  | | --- | --- | | Character | Name(s) | | : | colon | | ; | semicolon | | , | comma | | | | pipe | | # | hash, pound sign | | ~ | tilde | | \* | asterick | | + | plus | | = | equals | | > | greater than | | < | less than | | ( | left parenthesis | | ) | right parenthesis | | [ | left square bracket | | ] | right square bracket | | { | left curly brace | | } | right curly brace | | ^ | caret | | & | ampersand | | ! | exclamation mark | | @ | at sign | | $ | dollar sign | | % | percent | | - | dash, minus | | / | forward slash | | \ | back slash | | ? | question mark | | . | dot, period | |

  
  
