# Supplementary material for: The Systems Biology Research Tool: evolvable open-source software
Source: BMC Syst Biol. 2008 Jun 29;2:55. doi: 10.1186/1752-0509-2-55 (PMC2446383; doi:10.1186/1752-0509-2-55)
Supplement: Additional file 1 — SBRT Archive. An archive of the current version of the Systems Biology Research Tool. [file 1752-0509-2-55-S1.zip › sbrt-1.4.0/doc/users_guide/files/Text_Formatting_Rules.html]

Text Formatting Rules - Systems Biology Research Tool


|  |
| --- |
| > User's Guide |
|  |
| Text Formatting Rules All text files used as input to the Systems Biology Research Tool must conform to certain basic standards:   1. Lines must be terminated with a system-dependent new line    character. 2. Any leading or trailing whitespace characters (i.e. spaces,    tabs, etc.) in a line are ignored. 3. Blank or empty lines are ignored. 4. Any lines whose first non-whitespace character is the    pound/number sign "#" are ignored.   See Wikipedia for more information about new line characters. |
