# Supplementary material for: The Systems Biology Research Tool: evolvable open-source software
Source: BMC Syst Biol. 2008 Jun 29;2:55. doi: 10.1186/1752-0509-2-55 (PMC2446383; doi:10.1186/1752-0509-2-55)
Supplement: Additional file 1 — SBRT Archive. An archive of the current version of the Systems Biology Research Tool. [file 1752-0509-2-55-S1.zip › sbrt-1.4.0/doc/users_guide/files/Process_Name_Files.html]

Process Name Files - Systems Biology Research Tool


|  |
| --- |
| > User's Guide |
|  |
| Process Name Files *Process name files* are used to define the names of processes. These names are actually just nicknames for Java classes of a particular type. These classes are used by the Systems Biology Research Tool to perform a specific process. Process name files can be edited if a new process name is desired, or when a new process is being incorporated into the Systems Biology Research Tool. These files are a type of single-vector file whose variables are process names and whose values are Java class names.  A default process name file is included with each distribution of the Systems Biology Research Tool.  See the Text Formatting Rules for additional information. |
