# Supplementary material for: The Systems Biology Research Tool: evolvable open-source software
Source: BMC Syst Biol. 2008 Jun 29;2:55. doi: 10.1186/1752-0509-2-55 (PMC2446383; doi:10.1186/1752-0509-2-55)
Supplement: Additional file 1 — SBRT Archive. An archive of the current version of the Systems Biology Research Tool. [file 1752-0509-2-55-S1.zip › sbrt-1.4.0/doc/users_guide/files/File_Name_Files.html]

File Name Files - Systems Biology Research Tool


|  |
| --- |
| > User's Guide |
|  |
| File Name Files *File name files* are text files containing the names of input files or the desired names of output files. They are used when a process reads or writes a series of files. A single file name occurs on each line.  See the Text Formatting Rules for additional information. |
