# Supplementary material for: The Systems Biology Research Tool: evolvable open-source software
Source: BMC Syst Biol. 2008 Jun 29;2:55. doi: 10.1186/1752-0509-2-55 (PMC2446383; doi:10.1186/1752-0509-2-55)
Supplement: Additional file 1 — SBRT Archive. An archive of the current version of the Systems Biology Research Tool. [file 1752-0509-2-55-S1.zip › sbrt-1.4.0/doc/users_guide/index.html]

User's Guide - Systems Biology Research Tool


|  |
| --- |
| The Systems Biology Research Tool  User's Guide |
| This document describes the way in which the Systems Biology Research Tool can be used as an application. Comments, suggestions, or questions can be emailed to Jeremiah Wright at the address: . |

  
  

|  |  |
| --- | --- |
| Getting Started | Brief Descriptions |
| Processes | The functional unit of the Systems Biology Research Tool. |
| The Command Line | Instructions for using the Systems Biology Research Tool from the command line. |
| The Graphical User Interface | Instructions for using the Systems Biology Research Tool's GUI. |
| The Working Directory | A description of the Systems Biology Research Tool's working directory. |
| The Java Virtual Machine | A description of the JVM used by the Systems Biology Research Tool. |
|  |
| Process Categories | Brief Descriptions |
| Flux Balance Analysis | Used to study the flow of mass in biochemical reaction networks. |
| Graph Theory | Used to study the topology of biological interaction networks. |
| Geometry | Used to study the high-dimensional, geometrical objects formed by some models of biological systems. |
| Algebra | Used to perform algebraic analyses and to aid other analyses. |
| Combinatorics | Used to perform the combinatorial computations required by some algorithms used in systems biology. |
| Statistics | Used to compute statistics commonly used in systems biology. |
| Utilities | Used to perform helpful tasks. |
|  |
| Files | Brief Descriptions |
| Text Formatting Rules | The formatting rules for text-based input files. |
| Special Characters | The set of characters with special meaning to the SBRT. |
| File Names | The usage of file names. |
| File Formats | The currently supported file formats. |
| Process Files | Used to store the information required to execute a process. |
| Process Name Files | Used to store the names of each process. |
| File Name Files | Used to store the names of input files or the desired names of output files. |
|  |
| External Software | Brief Descriptions |
| Mathematica | Using Mathematica from the Systems Biology Research Tool. |
| Metatool | Using Metatool from the Systems Biology Research Tool. |
| Program Solvers | The currently supported linear program solvers. |
| R | Using R from the Systems Biology Research Tool. |
| XML Parsers | The currently supported XML parsers. |

  
  
  
