# Supplementary material for: The Systems Biology Research Tool: evolvable open-source software
Source: BMC Syst Biol. 2008 Jun 29;2:55. doi: 10.1186/1752-0509-2-55 (PMC2446383; doi:10.1186/1752-0509-2-55)
Supplement: Additional file 1 — SBRT Archive. An archive of the current version of the Systems Biology Research Tool. [file 1752-0509-2-55-S1.zip › sbrt-1.4.0/doc/users_guide/geometry/processes/Cd_Har.html]

Coordinate Directions Hit-and-Run - Systems Biology
Research Tool


|  |
| --- |
| > User's Guide > Geometry |
|  |
| Coordinate Directions Hit-and-Run This process is used to generate random points from within the interior of a convex polytope. The polytopes supplied to this process must be defined by pairs of parallel, bounding hyperplanes.  The random points generated by this process are the result of multiple "random walks" through the polytope - one walk along each coordinate axis. Stated differently, *n* walks are made through an *n* dimensional polytope to create the next *random point*. Each *random point* can be written to a specified output file, or each *i*-th random point can be written using the optional *points-per-point* keyword.  See the description of the algorithm used for additional information.  Here is the set of keywords this process understands, along with a description of their possible corresponding values. |

  


|  |  |
| --- | --- |
| Required Keywords | Possible Values |
| Process Name File | The name of the file where process names are defined. See  Process Name Files for further information. |
| Process | The name defined in the specified process name file.  Coordinate Directions Hit-and-Run is the default value. |
| Parallel Hyperplanes File | The name of the file containing the pairs of parallel hyperplanes used to define the convex polytope. See Parallel Hyperplanes Files for further information. |
| Initial Point File | The name of the file containing the initial point in the interior of the provided polytope. See Single-Vector Files for further information. |
| Output File Name | The desired name of the file to which random points will be written. See Multiple-Vectors Files for further information. |
| Iterations | The number of random points to be written to the specified output file. |
|  |
| Optional Keywords | Possible Values |
| Constraint Tolerance | The amount by which a generated point may lay outside of the defined constraints. The default value is 1E-9. See Constraint Tolerances for more information. |
| Points Per Point | The number of random points to generate for each point written to the specified output file. The default value is 1. |
| Minimum Chord Length | The minimum length of an unobstructed direction. The default value is 1E-6. |
| Seed | The seed for the pseudo-random number generator. The default value is the current time in milliseconds. |
| Maximum Obstructed Moves | The number of obstructed moves to allow before the process is terminated prematurely. The default value is 0. |

|  |
| --- |
|  |

|  |
| --- |
| Examples Click here for an example. |
