# Supplementary material for: The Systems Biology Research Tool: evolvable open-source software
Source: BMC Syst Biol. 2008 Jun 29;2:55. doi: 10.1186/1752-0509-2-55 (PMC2446383; doi:10.1186/1752-0509-2-55)
Supplement: Additional file 1 — SBRT Archive. An archive of the current version of the Systems Biology Research Tool. [file 1752-0509-2-55-S1.zip › sbrt-1.4.0/doc/users_guide/geometry/files/Parallel_Hyperplanes_Files.html]

Parallel Hyperplanes Files - Systems Biology Research
Tool


|  |
| --- |
| > User's Guide > Geometry |
|  |
| Parallel Hyperplanes Files |
| A hyperplane can be represented mathematically as: |
| **α**1 x1 + ... + **α**n xn = **β**. |
| A pair of parallel hyperplanes can, therefore, be represented as: |
| **α**1 x1 + ... + **α**n xn = **β**1 **α**1 x1 + ... + **α**n xn = **β**2. |
| A pair of parallel hyperplanes can be used to define a space |
| **β**lower ≤ **α**1 x1 + ... + **α**n xn ≤ **β**upper, |
| where the hyperplanes |
| **α**1 x1 + ... + **α**n xn = **β**lower **α**1 x1 + ... + **α**n xn = **β**upper. |
| define its boundaries. |
| A *parallel hyperplanes file* is a type of single-vector file used to store pairs of bounding parallel hyperplanes. The *variables* in these files are linear combinations, and the *values* are intervals. The linear combinations are used to represent the hyperplanes, and the intervals are used to represent the lower and upper values the hyperplanes can assume (**β**lower and **β**upper). Taken together, these elements form pairs of parallel bounding hyperplanes.  For example, the space formed by the inequality 0 ≤ x + y + z ≤ 1 is bounded by the pair of parallel hyperplanes x + y + z = 0 and x + y + z = 1. This could be represented in a parallel hyperplanes file as: x + y + z = [0; 1] See the Text Formatting Rules for additional information. |
