# Supplementary material for: The Systems Biology Research Tool: evolvable open-source software
Source: BMC Syst Biol. 2008 Jun 29;2:55. doi: 10.1186/1752-0509-2-55 (PMC2446383; doi:10.1186/1752-0509-2-55)
Supplement: Additional file 1 — SBRT Archive. An archive of the current version of the Systems Biology Research Tool. [file 1752-0509-2-55-S1.zip › sbrt-1.4.0/doc/users_guide/geometry/index.html]

Geometry - Systems Biology Research Tool


|  |
| --- |
| > User's Guide |
|  |
| Geometry |

  

|  |  |
| --- | --- |
| Processes | Brief Descriptions |
| Coordinate Directions Hit-and-Run | Used to generate random interior points within convex polytopes. |
|  |
| Files | Brief Descriptions |
| Parallel Hyperplanes Files | Used to store pairs of parallel hyperplanes. |
|  |
| Algorithms for Convex Polytopes | Brief Descriptions |
| Initial Point Generation | Used to generate interior points of convex polytopes. |
| Coordinate-Direction Hit-and-Run Algorithm | Used to find random, uniformly distributed points within convex polytopes. |
|  |
| Miscellaneous | Brief Descriptions |
| Constraint Tolerances | Used for numerical error checking. |
