# Supplementary material for: The Systems Biology Research Tool: evolvable open-source software
Source: BMC Syst Biol. 2008 Jun 29;2:55. doi: 10.1186/1752-0509-2-55 (PMC2446383; doi:10.1186/1752-0509-2-55)
Supplement: Additional file 1 — SBRT Archive. An archive of the current version of the Systems Biology Research Tool. [file 1752-0509-2-55-S1.zip › sbrt-1.4.0/doc/users_guide/geometry/algorithms/initial_points/index.html]

Initial Point Generation - Systems Biology Research Tool


|  |
| --- |
| > User's Guide > Geometry |
|  |
| Initial Point GenerationIntroduction The solution space of a bounded linear program can be represented as a convex polytope. Algorithms exist to sample points at random from within the interior of convex polytopes, and some of these algorithms require a starting point that lies in the interior of the polytope, rather than on an edge. The Systems Biology Research Tool currently uses a crude approximation of a barrier function to generate these initial interior points. This technique is described below. The Single-Variable System Let *V* denote a variable whose value is constrained to the interval [**α**, **β**], where **α** and **β** are finite real numbers, and **α** < **β**. This can be represented geometrically as a 1-dimensional convex polytope (a line segment), denoted as *X*. The values **α** and **β** are the edges of *X*.  Let *P* denote a new variable, referred to here as the *positioning* variable.  A functional relationship is defined for *P* and *V*, where *P = f(V)*. This function is used to create a solution space, referred to here as the *positioning space*, for a linear program. The requirement of the positioning space is: when the value of *P* is at its minimum, the value of *V* cannot lie on the edge of *X*. By projecting the 2-dimensional vector (Pmin, VPmin) onto the 1-dimensional polytope *X*, an interior point (VPmin) is generated.  In principle, many types of functions *f(V)* can be used to define the positioning space. With traditional barrier functions, the value of the positioning variable increases to infinity as the value of *V* approaches the boundary of the positioning space. In the technique currently used by the Systems Biology Research Tool, however, we restrict our possible positioning spaces to those that are convex and to those that can be created from hyperplanes. We do this so that a linear program solver can be used to compute the minimum value of the positioning variable. Example An example of a positioning space is depicted below.  The interval [**α**, **β**] on the V axis represents the original 1-dimensional convex polytope *X* (a line segment). The shaded region represents the positioning space, whose edges are defined by hyperplanes (straight lines). In this example, V = **α** + ½ (**β** - **α**) when the value of *P* is minimal.  This positioning space has been designed to cause the value of *V* to be equidistant from its endpoints when *P* assumes its minimal value. Note that the value of P decreases sharply when V is near the edges of the positioning space, and more gradually when V approaches the midpoint of the interval. This causes the value of V to be "strongly pushed away" from its end points when it is near them, and less so when it is further away. Multivariable Systems The method described above can be extrapolated to multivariable systems, as well. For each variable Vi in a system, a new positioning variable Pi is created, and a positioning space is created for each Vi-Pi pair. The sum of all Pi is minimized to generate a point in *X*. Caveats The extrapolation from the single variable technique to the multivariable technique described above may not be entirely satisfactory. It is conceivable that the values of some variables in a multivariable system may still lie on the edge of *X* after the sum of all positioning variables is minimized. This may or may not pose a problem for random walker algorithms (see the coordinate directions hit-and-run algorithm for additional details). Future versions of the Systems Biology Research Tool may implement a more robust technique for generating interior points in convex polytopes, especially if the current technique proves to be inadequate for some users. |
