# Supplementary material for: The Systems Biology Research Tool: evolvable open-source software
Source: BMC Syst Biol. 2008 Jun 29;2:55. doi: 10.1186/1752-0509-2-55 (PMC2446383; doi:10.1186/1752-0509-2-55)
Supplement: Additional file 1 — SBRT Archive. An archive of the current version of the Systems Biology Research Tool. [file 1752-0509-2-55-S1.zip › sbrt-1.4.0/doc/users_guide/geometry/algorithms/cd_har/index.html]

Coordinate Directions Hit-and-Run Algorithm - Systems
Biology Research Tool


|  |
| --- |
| > User's Guide > Geometry |
|  |
| Coordinate Directions Hit-and-Run Algorithm This algorithm was described in the paper:  Berbee, H.C.P.; Boender, C.G.G.; Rinnooy Kan, A.H.G.; Smith, R.L.; Telgen, J. *Hit-and-run algorithms for the identifcation of nonredundant linear inequalities.* Mathematical Programming 37 (1987) 184-207.  A graphical illustration is given below. Illustration First we start with a convex polytope that lies in 2 dimensional space with coordinates x and y.  Then an arbitrary point is chosen that lies within the interior of the polytope. See the initial points algorithm for one way in which this point can be chosen.  From this point, vectors can be created in the direction of each coordinate.  One of these directions is chosen at random to create a hyperplane (which in this case is just a line) through the polytope.  The intersection of this line with the polytope creates a chord, or line segment.  A point on this chord is then chosen randomly.  This process can be repeated by starting from the new point, resulting in a random walk through the interior of the polytope.  The limiting distribution of these points within the polytope is uniform. Also, notice what can happen if a starting point lies on a vertex, or corner, of the polytope.  Each direction along the coordinate axes is obstructed, and movement through the interior of the polytope becomes impossible. Fortunately, if the starting point lies in the interior of the polytope, the probability of reaching a corner during a random walk is virtually zero. (Mathematically, the probability equals zero, but with floating point arithmetic, the probability is slightly greater than zero.) |
