# Supplementary material for: The Systems Biology Research Tool: evolvable open-source software
Source: BMC Syst Biol. 2008 Jun 29;2:55. doi: 10.1186/1752-0509-2-55 (PMC2446383; doi:10.1186/1752-0509-2-55)
Supplement: Additional file 1 — SBRT Archive. An archive of the current version of the Systems Biology Research Tool. [file 1752-0509-2-55-S1.zip › sbrt-1.4.0/doc/users_guide/algebra/processes/Matrix_File_Conversion.html]

Matrix File Conversion - Systems Biology Research Tool


|  |
| --- |
| > User's Guide > Algebra |
|  |
| Matrix File Conversion This process is used to convert a matrix into a list of linear combinations. The file containing the matrix must be formatted as a multiple-vectors file, where the *values* are double precision numbers. The values of each vector in this input file are used to form a row of the matrix *A*. The *variables* in this input file are used to create a vector *x*. Linear combinations are created by computing the product *A x*. The *i*-th linear combination in the output file will correspond to the *i*-th row of the provided matrix. See the example below for further clarification.  Note that this process is the inverse of the Linear Combination File Conversion.  Here is the set of keywords this process understands, along with a description of their possible corresponding values. See the command line documentation for more information about keyword-value pairs. |

  


|  |  |
| --- | --- |
| Required Keywords | Possible Values |
| Process Name File | The name of the file where process names are defined. See  Process Name Files for further information. |
| Process | The name defined in the specified process name file.  Matrix File Conversion is the default value. |
| Input File | The name of the file containing the matrix. |
| Output File Name | The desired name of the file to which the system of linear expressions will be written. |

|  |
| --- |
|  |

|  |
| --- |
| Examples Click here for an example. |
