# Supplementary material for: The Systems Biology Research Tool: evolvable open-source software
Source: BMC Syst Biol. 2008 Jun 29;2:55. doi: 10.1186/1752-0509-2-55 (PMC2446383; doi:10.1186/1752-0509-2-55)
Supplement: Additional file 1 — SBRT Archive. An archive of the current version of the Systems Biology Research Tool. [file 1752-0509-2-55-S1.zip › sbrt-1.4.0/doc/users_guide/algebra/processes/Linear_System_Solver.html]

Linear System Solver - Systems Biology Research Tool


|  |
| --- |
| > User's Guide > Algebra |
|  |
| Linear System Solver This process is used to solve systems of linear equations using Mathematica.  Here is the set of keywords this process understands, along with a description of their possible corresponding values. See the command line documentation for more information about keyword-value pairs. |

  


|  |  |
| --- | --- |
| Required Keywords | Possible Values |
| Process Name File | The name of the file where process names are defined. See  Process Name Files for further information. |
| Process | The name defined in the specified process name file.  Linear System Solver is the default value. |
| Equations File | The name of the file containing the system of linear equations. See Linear Equation Files for further information. |
| Kernel Link Command | The command used to link to the Mathematica kernel. See the Mathematica documentation for additional information. |
| Output File Name | The desired name of the file to which the solution will be written. See Linear System Solution Files for further information. |

|  |
| --- |
|  |

|  |
| --- |
| Examples Click here for an example. |
