# Supplementary material for: The Systems Biology Research Tool: evolvable open-source software
Source: BMC Syst Biol. 2008 Jun 29;2:55. doi: 10.1186/1752-0509-2-55 (PMC2446383; doi:10.1186/1752-0509-2-55)
Supplement: Additional file 1 — SBRT Archive. An archive of the current version of the Systems Biology Research Tool. [file 1752-0509-2-55-S1.zip › sbrt-1.4.0/doc/users_guide/algebra/processes/Single_Vector_File_Conversion.html]

Single-Vector File Conversion - Systems Biology Research
Tool


|  |
| --- |
| > User's Guide > Algebra |
|  |
| Single-Vector File Conversion This process is used to convert multiple single-vector files into a single multiple-vectors file. Neither the *variables* nor *values* contained in the input files are parsed during this process; therefore, they can be of nearly any type (double precision numbers, intervals, etc.). The vector contained in the *i*-th specified input file is written to the *i*-th line of the specified output file. See the example below for further clarification.  Note that this process is the inverse of the Multiple-Vectors File Conversion.  Here is the set of keywords this process understands, along with a description of their possible corresponding values. See the command line documentation for more information about keyword-value pairs. |

  


|  |  |
| --- | --- |
| Required Keywords | Possible Values |
| Process Name File | The name of the file where process names are defined. See  Process Name Files for further information. |
| Process | The name defined in the specified process name file.  Single-Vector File Conversion is the default value. |
| Input File Name File | The name of the file containing the names of the single-vector files. See File Name Files for additional information.. |
| Data Headers | A pipe-delimited set of variables that will appear in the generated output file. |
| Output File Name | The desired name of the multiple-vectors output file |

|  |
| --- |
|  |

|  |
| --- |
| Examples Click here for an example. |
