# Supplementary material for: The Systems Biology Research Tool: evolvable open-source software
Source: BMC Syst Biol. 2008 Jun 29;2:55. doi: 10.1186/1752-0509-2-55 (PMC2446383; doi:10.1186/1752-0509-2-55)
Supplement: Additional file 1 — SBRT Archive. An archive of the current version of the Systems Biology Research Tool. [file 1752-0509-2-55-S1.zip › sbrt-1.4.0/doc/users_guide/algebra/processes/Linear_Combination_File_Conversion.html]

Linear Combination File Conversion - Systems Biology
Research Tool


|  |
| --- |
| > User's Guide > Algebra |
|  |
| Linear Combination File Conversion This process is used to convert a list of linear combinations into a matrix. The input file must contain a list of linear combinations, with one expression per line. The output file will be formatted as a multiple-vectors file, where the *variables* are obtained from the provided linear combinations and the *values* are the double precision numbers representing the coefficients of the provided linear combinations. If a provided linear combination contains a non-zero constant, this constant will *not* appear in the generated matrix file. The *i*-th row of the matrix in the generated output file will correspond to the *i*-th linear combination in the input file. See the example below for further clarification.  Note that this process is the inverse of the Matrix File Conversion.  Here is the set of keywords this process understands, along with a description of their possible corresponding values. See the command line documentation for more information about keyword-value pairs. |

  


|  |  |
| --- | --- |
| Required Keywords | Possible Values |
| Process Name File | The name of the file where process names are defined. See  Process Name Files for further information. |
| Process | The name defined in the specified process name file.  Linear Combination File Conversion is the default value. |
| Input File | The name of the file containing the list of linear combinations. |
| Output File Name | The desired name of the file to which the matrix will be written. |

|  |
| --- |
|  |

|  |
| --- |
| Examples Click here for an example. |
