# Supplementary material for: The Systems Biology Research Tool: evolvable open-source software
Source: BMC Syst Biol. 2008 Jun 29;2:55. doi: 10.1186/1752-0509-2-55 (PMC2446383; doi:10.1186/1752-0509-2-55)
Supplement: Additional file 1 — SBRT Archive. An archive of the current version of the Systems Biology Research Tool. [file 1752-0509-2-55-S1.zip › sbrt-1.4.0/doc/users_guide/algebra/files/Linear_Equation_Files.html]

Linear Equation Files - Systems Biology Research Tool


|  |
| --- |
| > User's Guide > Algebra |
|  |
| Linear Equation Files A *linear equation file* is a type of single-vector file containing a system of linear equations. The *variables* in these files are linear combinations representing the left-hand sides of the equations, and the *values* are double precision numbers representing the right-hand sides of the equations.  See the Text Formatting Rules for additional information. |
