# Supplementary material for: The Systems Biology Research Tool: evolvable open-source software
Source: BMC Syst Biol. 2008 Jun 29;2:55. doi: 10.1186/1752-0509-2-55 (PMC2446383; doi:10.1186/1752-0509-2-55)
Supplement: Additional file 1 — SBRT Archive. An archive of the current version of the Systems Biology Research Tool. [file 1752-0509-2-55-S1.zip › sbrt-1.4.0/doc/users_guide/algebra/files/Single_Interval_Vector_Files.html]

Single-Interval-Vector Files - Systems Biology Research
Tool


|  |
| --- |
| > User's Guide > Algebra |
|  |
| Single-Interval-Vector Files A *single-interval-vector file* is a type of single-vector file. The values of the vectors contained in this type of file are intervals. |
