# Supplementary material for: The Systems Biology Research Tool: evolvable open-source software
Source: BMC Syst Biol. 2008 Jun 29;2:55. doi: 10.1186/1752-0509-2-55 (PMC2446383; doi:10.1186/1752-0509-2-55)
Supplement: Additional file 1 — SBRT Archive. An archive of the current version of the Systems Biology Research Tool. [file 1752-0509-2-55-S1.zip › sbrt-1.4.0/doc/users_guide/algebra/files/Single_Vector_Files.html]

Single-Vector Files - Systems Biology Research Tool


|  |
| --- |
| > User's Guide > Algebra |
|  |
| Single-Vector Files A *single-vector file* is a text file used to store a single vector. Here, a vector is considered to be a set of *variables* with a corresponding *value* for each. Each line contains a single variable-value pair. The syntax is: Variable = Value. Neither variables nor values can contain the equals character "=", and each variable can appear only once in the file. Any whitespace characters around the equals sign are ignored.  See the Text Formatting Rules for additional information. |
