# Supplementary material for: The Systems Biology Research Tool: evolvable open-source software
Source: BMC Syst Biol. 2008 Jun 29;2:55. doi: 10.1186/1752-0509-2-55 (PMC2446383; doi:10.1186/1752-0509-2-55)
Supplement: Additional file 1 — SBRT Archive. An archive of the current version of the Systems Biology Research Tool. [file 1752-0509-2-55-S1.zip › sbrt-1.4.0/doc/users_guide/algebra/files/Multiple_Vectors_Files.html]

Multiple-Vectors Files - Systems Biology Research Tool


|  |
| --- |
| > User's Guide > Algebra |
|  |
| Multiple-Vectors Files A *multiple-vectors file* is a text file used to store multiple vectors. Here, a vector is considered to be a set of *variables* with a corresponding value for each. The first line in a file is a pipe-delimited set of variables, with the syntax: Variable\_1 | Variable\_2 | ... | Variable\_N Each variable can appear only once in this set. The subsequent lines of the file are pipe-delimited lists of values, with the syntax: Value\_1 | Value\_2 | ... | Value\_N Neither variables nor values can contain the pipe character "|". Any whitespace characters around pipes are ignored. If *n* variables occur in the variable set, *n* values must also occur in each subsequent value list. The correspondence between variables and their values is based on order: the *i*-th value corresponds to the *i*-th variable.  See the Text Formatting Rules for additional information. |
