# Supplementary material for: The Systems Biology Research Tool: evolvable open-source software
Source: BMC Syst Biol. 2008 Jun 29;2:55. doi: 10.1186/1752-0509-2-55 (PMC2446383; doi:10.1186/1752-0509-2-55)
Supplement: Additional file 1 — SBRT Archive. An archive of the current version of the Systems Biology Research Tool. [file 1752-0509-2-55-S1.zip › sbrt-1.4.0/doc/users_guide/algebra/formats/Intervals.html]

Intervals - Systems Biology Research Tool


|  |
| --- |
| > User's Guide > Algebra |
|  |
| Intervals Intervals are composed of a lower and upper bound, and they have the following syntax:  [Lower\_Bound; Upper\_Bound].   The lower and upper bounds must be parsable as double precision numbers. Note that positive and negative infinity can be denoted respectively as Infinity and -Infinity. The upper bound must be greater than or equal to the lower bound. To indicate an interval with a range of zero, both bounds should be identical. Any whitespace around the semicolon and brackets is ignored. |
