# Supplementary material for: The Systems Biology Research Tool: evolvable open-source software
Source: BMC Syst Biol. 2008 Jun 29;2:55. doi: 10.1186/1752-0509-2-55 (PMC2446383; doi:10.1186/1752-0509-2-55)
Supplement: Additional file 1 — SBRT Archive. An archive of the current version of the Systems Biology Research Tool. [file 1752-0509-2-55-S1.zip › sbrt-1.4.0/doc/users_guide/algebra/formats/Linear_Combinations.html]

Linear Combinations - Systems Biology Research Tool


|  |
| --- |
| > User's Guide > Algebra |
|  |
| Linear Combinations A linear combination is composed of a set of *terms* and a *constant* separated by plus signs "+". Any whitespace characters around plus signs are ignored. Terms are composed of a coefficient and a variable, and they have the syntax: (Coefficient) Variable. At least one whitespace character must exist between the variable and the closing parenthesis of the coefficient. Constants have the syntax: (Constant). Each term of a linear combination must contain a unique variable. The constant can appear anywhere within the linear combination, but only one constant is allowed. Coefficients and constants must be parsable as finite double precision numbers. If a coefficient equals unity, it can be omitted entirely. If a constant equals zero, it can be omitted entirely.  For example, the linear combination *x + y* could be represented as any of the following:   |  | | --- | | x + y | | (1) x + y | | x + (1) y | | x + y + (0) | | (1) x + y + (0) | | x + (1) y + (0) | | (1) x + (1) y + (0) | |
