# Supplementary material for: The Systems Biology Research Tool: evolvable open-source software
Source: BMC Syst Biol. 2008 Jun 29;2:55. doi: 10.1186/1752-0509-2-55 (PMC2446383; doi:10.1186/1752-0509-2-55)
Supplement: Additional file 1 — SBRT Archive. An archive of the current version of the Systems Biology Research Tool. [file 1752-0509-2-55-S1.zip › sbrt-1.4.0/doc/users_guide/algebra/formats/Double_Precision_Numbers.html]

Double Precision Numbers - Systems Biology Research Tool


|  |
| --- |
| > User's Guide > Algebra |
|  |
| Double Precision Numbers Double precision numbers in the Systems Biology Research Tool conform to the  Java Language Specification.  See  java.lang.Double.valueOf(String s) and  java.lang.Double.toString(double d) for detailed descriptions of the way in which the Systems Biology Research Tool parses and formats double precision numbers. |
