# Supplementary material for: The Systems Biology Research Tool: evolvable open-source software
Source: BMC Syst Biol. 2008 Jun 29;2:55. doi: 10.1186/1752-0509-2-55 (PMC2446383; doi:10.1186/1752-0509-2-55)
Supplement: Additional file 1 — SBRT Archive. An archive of the current version of the Systems Biology Research Tool. [file 1752-0509-2-55-S1.zip › sbrt-1.4.0/doc/users_guide/algebra/index.html]

Algebra - Systems Biology Research Tool


|  |
| --- |
| > User's Guide |
|  |
| Algebra |

  

|  |  |
| --- | --- |
| Processes | Brief Descriptions |
| Linear System Solver | Used to solve systems of linear equations using Mathematica. |
| Multiple-Vectors File Conversion | Used to convert a single multiple-vectors file into multiple single-vector files. |
| Single-Vector File Conversion | Used to convert multiple single-vector files into a single multiple-vectors file. |
| Matrix File Conversion | Used to convert a matrix into a list of linear combinations. |
| Linear Combination File Conversion | Used to convert a list of linear combinations into a matrix. |
|  |
| Text Formats | Brief Descriptions |
| Double Precision Numbers | The format of real numbers. |
| Intervals | The format of intervals. |
| Linear Combinations | The format of linear combinations. |
|  |
| Files | Brief Descriptions |
| Single-Vector Files | Used to store a single vector. |
| Multiple-Vectors Files | Used to store multiple vectors. |
| Single-Interval-Vector Files | Used to store a single vector whose values are intervals. |
| Multiple-Interval-Vectors Files | Used to store multiple vectors whose values are intervals. |
| Linear Equation Files | Used to store a system of linear equations. |
| Linear System Solution Files | Used to store the solution to a system of linear equations. |

  
  
