# Supplementary material for: The Systems Biology Research Tool: evolvable open-source software
Source: BMC Syst Biol. 2008 Jun 29;2:55. doi: 10.1186/1752-0509-2-55 (PMC2446383; doi:10.1186/1752-0509-2-55)
Supplement: Additional file 1 — SBRT Archive. An archive of the current version of the Systems Biology Research Tool. [file 1752-0509-2-55-S1.zip › sbrt-1.4.0/doc/users_guide/combinatorics/processes/Single_Element_Unions.html]

Single-Element Unions - Systems Biology
Research Tool


|  |
| --- |
| > User's Guide > Combinatorics |
|  |
| Single-Element Unions This process is used to compute all possible *single-element unions* of a collection of sets.  Let *C* denote a collection of sets, and let *Ci* denote the *i*-th set in *C*. Single-element unions are created by sampling a single element from each *Ci*. Single-element unions are themselves sets, and they contain (at least) one element from each *Ci*. If all *Ci* are disjoint, then each single-element union created from *C* will contain only a single element from each *Ci*. If some *Ci* contain elements in common, however, a single-element union may contain multiple elements from a single *Ci*. Note the difference between single-element unions and strict single-element unions.  For example, the single-element unions of the collection of sets:   |  |  | | --- | --- | |  | {A, B, C}, | |  | {C, D}, | |  | {E}, |   are:   |  |  | | --- | --- | |  | {A, C, E}, | |  | {A, D, E}, | |  | {B, C, E}, | |  | {B, D, E}, | |  | {C, E}, | |  | {C, D, E}. |   Here is the set of keywords this process understands, along with a description of their possible corresponding values. See the command line documentation for more information about keyword-value pairs. |

  


|  |  |
| --- | --- |
| Required Keywords | Possible Values |
| Process Name File | The name of the file where process names are defined. See  Process Name Files for further information. |
| Process | The name defined in the specified process name file.  Single-Element Unions is the default value. |
| Set File | The name of the file containing a collection of sets. See Set Files for further information. |
| Output File Name | The desired name of the file where the single-element unions will be stored. See Set Files for further information. |

|  |
| --- |
|  |

|  |
| --- |
| Examples Click here for an example. |
