# Supplementary material for: The Systems Biology Research Tool: evolvable open-source software
Source: BMC Syst Biol. 2008 Jun 29;2:55. doi: 10.1186/1752-0509-2-55 (PMC2446383; doi:10.1186/1752-0509-2-55)
Supplement: Additional file 1 — SBRT Archive. An archive of the current version of the Systems Biology Research Tool. [file 1752-0509-2-55-S1.zip › sbrt-1.4.0/doc/users_guide/combinatorics/files/Set_Files.html]

Set Files - Systems Biology Research Tool


|  |
| --- |
| > User's Guide > Combinatorics |
|  |
| Set Files A *set file* is a text file used to store pipe-delimited sets, with a single set on each line. The line syntax is: Element\_1 | Element\_2 | ... | Element\_N Each element can appear only once in each set. Any whitespace characters around the pipes "|" are ignored. Elements cannot contain any pipes themselves.  See the Text Formatting Rules for additional information. |
