# Supplementary material for: The Systems Biology Research Tool: evolvable open-source software
Source: BMC Syst Biol. 2008 Jun 29;2:55. doi: 10.1186/1752-0509-2-55 (PMC2446383; doi:10.1186/1752-0509-2-55)
Supplement: Additional file 1 — SBRT Archive. An archive of the current version of the Systems Biology Research Tool. [file 1752-0509-2-55-S1.zip › sbrt-1.4.0/doc/users_guide/combinatorics/index.html]

Combinatorics - Systems Biology Research Tool


|  |
| --- |
| > User's Guide |
|  |
| Combinatorics |

  

|  |  |
| --- | --- |
| Processes | Brief Descriptions |
| Single-Element Unions | Used to compute single-element unions of collections of sets. |
| Strict Single-Element Unions | Used to compute strict single-element unions of collections of sets. |
|  |
| Files | Brief Descriptions |
| Set Files | Used to store a collection of sets. |
