# Supplementary material for: The Systems Biology Research Tool: evolvable open-source software
Source: BMC Syst Biol. 2008 Jun 29;2:55. doi: 10.1186/1752-0509-2-55 (PMC2446383; doi:10.1186/1752-0509-2-55)
Supplement: Additional file 1 — SBRT Archive. An archive of the current version of the Systems Biology Research Tool. [file 1752-0509-2-55-S1.zip › sbrt-1.4.0/doc/users_guide/statistics/processes/Kendalls_Tau.html]

Kendall's Tau Correlation - Systems Biology
Research Tool


|  |
| --- |
| > User's Guide > Statistics |
|  |
| Kendall's Tau Correlation This process is used to compute Kendall's rank correlation statistics τa and τb between two vectors *x* and *y*. The sets of variables in *x* and *y* do not have to be identical, but their intersection should not be empty. The computed statistic is written to stdout. If an output file name is provided, the intersection of *x* and *y* values will be written to it.  For additional information, see: Kendall, Maurice and Jean Dickinson Gibbons (1990). Rank Correlation Methods, Fifth Edition. NY: Oxford Univ Press; ISBN: 0195208374.  Here is the set of keywords this process understands, along with a description of their possible corresponding values. See the command line documentation for more information about keyword-value pairs. |

  


|  |  |
| --- | --- |
| Required Keywords | Possible Values |
| Process Name File | The name of the file where process names are defined. See  Process Name Files for further information. |
| Process | The name defined in the specified process name file.  Kendall's Tau Correlation is the default value. |
| Correlation Statistic | Kendall's Tau A or Kendall's Tau B |
| X Value File Name | The name of a single-vector file containing the *x* values. |
| Y Value File Name | The name of a single-vector file containing the *y* values. |
|  |
| Optional Keywords | Possible Values |
| Output File Name | The name of the file to be created by this process. |

|  |
| --- |
|  |

|  |
| --- |
| Examples Click here for an example. |
