# Supplementary material for: The Systems Biology Research Tool: evolvable open-source software
Source: BMC Syst Biol. 2008 Jun 29;2:55. doi: 10.1186/1752-0509-2-55 (PMC2446383; doi:10.1186/1752-0509-2-55)
Supplement: Additional file 1 — SBRT Archive. An archive of the current version of the Systems Biology Research Tool. [file 1752-0509-2-55-S1.zip › sbrt-1.4.0/doc/users_guide/statistics/files/Numerical_Values_Files.html]

Numerical Values Files - Systems Biology
Research Tool


|  |
| --- |
| > User's Guide > Statistics |
|  |
| Numerical Values Files A *numerical values file* is a text file used to store numerical values. Each line contains a single double precision number. See the Text Formatting Rules for additional information. |
