# Supplementary material for: The Systems Biology Research Tool: evolvable open-source software
Source: BMC Syst Biol. 2008 Jun 29;2:55. doi: 10.1186/1752-0509-2-55 (PMC2446383; doi:10.1186/1752-0509-2-55)
Supplement: Additional file 1 — SBRT Archive. An archive of the current version of the Systems Biology Research Tool. [file 1752-0509-2-55-S1.zip › sbrt-1.4.0/doc/users_guide/statistics/index.html]

Statistical Analysis - Systems Biology Research Tool


|  |
| --- |
| > User's Guide |
|  |
| Statistics |

  

|  |  |
| --- | --- |
| Processes | Brief Descriptions |
| Correlation Estimation | Used to compute a variety of correlation coefficients using R. |
| Kendall's Tau Correlation | Used to compute Kendall's tau correlation statistics. |
| Mann-Whitney U Test | Used to compute Mann-Whitney U statistics. |
|  |
| Files | Brief Descriptions |
| Numerical Values Files | Used to store a list of numerical values. |
