# Supplementary material for: The Systems Biology Research Tool: evolvable open-source software
Source: BMC Syst Biol. 2008 Jun 29;2:55. doi: 10.1186/1752-0509-2-55 (PMC2446383; doi:10.1186/1752-0509-2-55)
Supplement: Additional file 1 — SBRT Archive. An archive of the current version of the Systems Biology Research Tool. [file 1752-0509-2-55-S1.zip › sbrt-1.4.0/doc/users_guide/utilities/processes/Variable_Participation.html]

Variable Participation - Systems Biology Research Tool


|  |
| --- |
| > User's Guide > Utilities |
|  |
| Variable Participation This process is used to group mathematical expressions based on the variables they contain. Let *E* denote a set of mathematical expressions, and let *V* denote the set of all variables contained in the expressions in *E*. For each element *v* in *V*, a corresponding subset *S* of *E* exists that contains the expressions containing the variable *v*. This process writes the mapping of each variable *v* to its set of expressions *S* to an output file.  Here is the set of keywords this process understands, along with a description of their possible corresponding values. See the command line documentation for more information about keyword-value pairs. |

  


|  |  |
| --- | --- |
| Required Keywords | Possible Values |
| Process Name File | The name of the file where process names are defined. See  Process Name Files for further information. |
| Process | The name defined in the specified process name file.  Variable Participation is the default value. |
| Expression File | The name of the file containing the mathematical expressions, one per line. Currently, these expressions must be parsable as linear combinations. |
| Output File Name | The name of the file to be created by this process. |

|  |
| --- |
|  |

|  |
| --- |
| Examples Click here for an example. |
