# Supplementary material for: The Systems Biology Research Tool: evolvable open-source software
Source: BMC Syst Biol. 2008 Jun 29;2:55. doi: 10.1186/1752-0509-2-55 (PMC2446383; doi:10.1186/1752-0509-2-55)
Supplement: Additional file 1 — SBRT Archive. An archive of the current version of the Systems Biology Research Tool. [file 1752-0509-2-55-S1.zip › sbrt-1.4.0/doc/users_guide/utilities/processes/Interval_Comparison.html]

Interval Comparison - Systems Biology Research Tool


|  |
| --- |
| > User's Guide > Utilities |
|  |
| Interval Comparison This process is used to compare two sets of vectors whose values are intervals. The *i*-th vector in the first set is compared to the *i*-th vector in the second set. If these vectors are not equivalent, i.e. their intervals differ, their differences are written to an output file.  Here is the set of keywords this process understands, along with a description of their possible corresponding values. See the command line documentation for more information about keyword-value pairs. |

  


|  |  |
| --- | --- |
| Required Keywords | Possible Values |
| Process Name File | The name of the file where process names are defined. See  Process Name Files for further information. |
| Process | The name defined in the specified process name file.  Interval Comparison is the default value. |
| File 1 | The name of the multiple-interval-vectors file containing the first set of vectors. |
| File 2 | The name of the multiple-interval-vectors file containing the second set of vectors. |
| Zero Cutoff | The value by which two numerical values can differ but still be considered equivalent, such as 1E-6. |
| Output File Name | The name of the file to which the differences between the two sets of vectors will be written. |

|  |
| --- |
|  |

|  |
| --- |
| Examples Click here for an example. |
