# Supplementary material for: The Systems Biology Research Tool: evolvable open-source software
Source: BMC Syst Biol. 2008 Jun 29;2:55. doi: 10.1186/1752-0509-2-55 (PMC2446383; doi:10.1186/1752-0509-2-55)
Supplement: Additional file 1 — SBRT Archive. An archive of the current version of the Systems Biology Research Tool. [file 1752-0509-2-55-S1.zip › sbrt-1.4.0/doc/users_guide/utilities/processes/Numerical_Vector_Comparison.html]

Numerical Vector Comparison - Systems Biology Research
Tool


|  |
| --- |
| > User's Guide > Utilities |
|  |
| Numerical Vector Comparison This process is used to perform a pair-wise comparison of all vectors in a given set whose values are strictly numerical. If a pair of vectors is found to be equivalent, i.e. their numerical values differ by less than a defined tolerance, that pair is written to an output file, and the process halts.  Here is the set of keywords this process understands, along with a description of their possible corresponding values. See the command line documentation for more information about keyword-value pairs. |

  


|  |  |
| --- | --- |
| Required Keywords | Possible Values |
| Process Name File | The name of the file where process names are defined. See  Process Name Files for further information. |
| Process | The name defined in the specified process name file.  Numerical Vector Comparison is the default value. |
| File | The name of a multiple-vectors file containing the set of vectors. |
| Zero Cutoff | The value by which two numerical values can differ but still be considered equivalent, such as 1E-6. |
| Output File Name | The name of the file to which the first pair of equivalent vectors will be written. |
|  |
| Optional Keywords | Possible Values |
| Output File Name | The name of the file to be created by this process. |

|  |
| --- |
|  |

|  |
| --- |
| Examples Click here for an example. |
