# Supplementary material for: The Systems Biology Research Tool: evolvable open-source software
Source: BMC Syst Biol. 2008 Jun 29;2:55. doi: 10.1186/1752-0509-2-55 (PMC2446383; doi:10.1186/1752-0509-2-55)
Supplement: Additional file 1 — SBRT Archive. An archive of the current version of the Systems Biology Research Tool. [file 1752-0509-2-55-S1.zip › sbrt-1.4.0/doc/users_guide/external_software/Mathematica/index.html]

Mathematica - Systems Biology Research Tool


|  |
| --- |
| > User's Guide |
|  |
| Mathematica Mathematica is currently used by two processes of the Systems Biology Research Tool. Mathematica must be installed on your system to use these particular processes, and the following must be done to make Mathematica accessible to the SBRT:  (1) The file JLink.jar must be copied into the SBRT's lib directory.  In Windows, this file may be located in: C:\Program Files\Wolfram Research\Mathematica\5.2\AddOns\JLink In Linux: /usr/local/Wolfram/Mathematica/5.2/AddOns/JLink In Mac OS X: /Applications/Mathematica 5.2.app/AddOns/JLink (2) A command must be provided to SBRT processes to allow linking with the Mathematica kernel.  In Windows, this command should be similar to: -linkmode launch -linkname 'c:\\program files\\wolfram research\\mathematica\\5.2\\mathkernel.exe' In Linux: -linkmode launch -linkname 'math -mathlink' In Mac OS X: -linkmode launch -linkname '\"/Applications/Mathematica 5.2.app/Contents/MacOS/MathKernel\" -mathlink' These commands should be used as values whenever the keyword Kernel Link Command is used.  See the Mathematica J/Link method MathLinkFactory.createKernelLink(java.lang.String cmdLine) for additional information. |

  
  
