# Supplementary material for: The Systems Biology Research Tool: evolvable open-source software
Source: BMC Syst Biol. 2008 Jun 29;2:55. doi: 10.1186/1752-0509-2-55 (PMC2446383; doi:10.1186/1752-0509-2-55)
Supplement: Additional file 1 — SBRT Archive. An archive of the current version of the Systems Biology Research Tool. [file 1752-0509-2-55-S1.zip › sbrt-1.4.0/doc/users_guide/external_software/R/index.html]

R - Systems Biology Research Tool


|  |
| --- |
| > User's Guide |
|  |
| R R is currently used by the Systems Biology Research Tool's Correlation Estimation process. This is accomplished by using a Java/R interface (JRI). To use this process, both R and JRI must be installed on your system. Installing R Instructions for downloading and installing R in Windows, Macintosh, and Linux can be found on R's main page and in the R FAQ. Installing JRI Instructions for downloading and compiling JRI can be found on its main page. The java archive (JRI.jar) and the shared object file (libjri.so), or the dynamic-link library (jri.dll), must be placed in the System Biology Research Tool's lib directory. In Linux (at least), the environment variable R\_HOME must be set to its appropriate value as well. This could be done in bash, for example, by executing the following command: R\_HOME=/usr/lib/R ; export R\_HOME |

  
  
