# Supplementary material for: The Systems Biology Research Tool: evolvable open-source software
Source: BMC Syst Biol. 2008 Jun 29;2:55. doi: 10.1186/1752-0509-2-55 (PMC2446383; doi:10.1186/1752-0509-2-55)
Supplement: Additional file 1 — SBRT Archive. An archive of the current version of the Systems Biology Research Tool. [file 1752-0509-2-55-S1.zip › sbrt-1.4.0/doc/users_guide/external_software/xml_parsers/index.html]

XML Parsers - Systems Biology Research Tool


|  |
| --- |
| > User's Guide |
|  |
| XML Parsers The XML parser Xerces from the Apache Software Foundation is currently supported by the Systems Biology Research Tool. The files xercesImpl.jar and xercesImpl.LICENSE.txt are included in the SBRT's lib directory, and therefore:  **This product includes software developed by the Apache Software Foundation (http://www.apache.org/).** |

  
  
