# Supplementary material for: The Systems Biology Research Tool: evolvable open-source software
Source: BMC Syst Biol. 2008 Jun 29;2:55. doi: 10.1186/1752-0509-2-55 (PMC2446383; doi:10.1186/1752-0509-2-55)
Supplement: Additional file 1 — SBRT Archive. An archive of the current version of the Systems Biology Research Tool. [file 1752-0509-2-55-S1.zip › sbrt-1.4.0/doc/users_guide/external_software/program_solvers/index.html]

Program Solvers - Systems Biology Research Tool


|  |
| --- |
| > User's Guide |
|  |
| Program Solvers Two program solvers are currently supported by the Systems Biology Research Tool: the GNU Linear Programming Kit (GLPK) and CPLEX. The strings GLPK and CPLEX  must be used to denote the name of the program solver when one is required.  GLPK is packaged with the Systems Biology Research Tool, so separate installation should not be required. If a problem is encountered with GLPK, please consult the external software downloads page for more information.  To use CPLEX, place cplex.jar and the appropriate shared object file in the SBRT's lib directory. |

  
  


|  |  |
| --- | --- |
| Text Formats | Brief Descriptions |
| Optimization Senses | The format of optimization senses. |
|  |
| Files | Brief Descriptions |
| Program Solver Parameter Files | Used to store parameters for linear program solvers. |
