# Supplementary material for: The Systems Biology Research Tool: evolvable open-source software
Source: BMC Syst Biol. 2008 Jun 29;2:55. doi: 10.1186/1752-0509-2-55 (PMC2446383; doi:10.1186/1752-0509-2-55)
Supplement: Additional file 1 — SBRT Archive. An archive of the current version of the Systems Biology Research Tool. [file 1752-0509-2-55-S1.zip › sbrt-1.4.0/doc/users_guide/external_software/program_solvers/Ps_Param_Files.html]

Program Solver Parameter Files - Systems Biology Research
Tool


|  |
| --- |
| > User's Guide > Program Solvers |
|  |
| Program Solver Parameter Files Files of this type allow the user to set the parameters of the program solver. Currently, GLPK does not support such files. CPLEX understands PRM files, which are described in the documentation that comes with CPLEX. |
