# Supplementary material for: The Systems Biology Research Tool: evolvable open-source software
Source: BMC Syst Biol. 2008 Jun 29;2:55. doi: 10.1186/1752-0509-2-55 (PMC2446383; doi:10.1186/1752-0509-2-55)
Supplement: Additional file 1 — SBRT Archive. An archive of the current version of the Systems Biology Research Tool. [file 1752-0509-2-55-S1.zip › sbrt-1.4.0/doc/users_guide/external_software/program_solvers/Optimization_Senses.html]

Optimization Senses - Systems Biology Research Tool


|  |
| --- |
| > User's Guide > Program Solvers |
|  |
| Optimization Senses An optimization sense is used to specify the direction in which an optimization problem should be solved. The strings Maximize and Minimize are used to denote these directions. |
