# Supplementary material for: The Systems Biology Research Tool: evolvable open-source software
Source: BMC Syst Biol. 2008 Jun 29;2:55. doi: 10.1186/1752-0509-2-55 (PMC2446383; doi:10.1186/1752-0509-2-55)
Supplement: Additional file 1 — SBRT Archive. An archive of the current version of the Systems Biology Research Tool. [file 1752-0509-2-55-S1.zip › sbrt-1.4.0/doc/users_guide/external_software/Metatool/index.html]

Metatool - Systems Biology Research Tool


|  |
| --- |
| > User's Guide |
|  |
| Metatool The Systems Biology Research Tool provides indirect support for Metatool 4.9.2 and 5.0. The process Metatool File Writer can be used to convert FBA Reaction Files into text-based input files for Metatool.  See the main Metatool page for additional information. |

  
  
