# Supplementary material for: The Systems Biology Research Tool: evolvable open-source software
Source: BMC Syst Biol. 2008 Jun 29;2:55. doi: 10.1186/1752-0509-2-55 (PMC2446383; doi:10.1186/1752-0509-2-55)
Supplement: Additional file 1 — SBRT Archive. An archive of the current version of the Systems Biology Research Tool. [file 1752-0509-2-55-S1.zip › sbrt-1.4.0/doc/developers_guide/notes/Kernel-Shell_Interactions.html]

Kernel-Shell Interactions - Systems Biology Research Tool


|  |
| --- |
| > Developer's Guide |
|  |
| Kernel-Shell Interactions When developing process plug-ins, the kernel of your plug-in should be completely unaware of its shell. In other words, kernel-level classes should never call shell-level classes.  The shell should catch all exceptions thrown by the kernel and intrpret them in an appropriate way. If the error is unrecoverable, the ProcessManager should throw an ApplicationException, or one of its subclasses. The kernel should *never* throw an ApplicationException.  More to come (especially upon request) ... |
