# Supplementary material for: The Systems Biology Research Tool: evolvable open-source software
Source: BMC Syst Biol. 2008 Jun 29;2:55. doi: 10.1186/1752-0509-2-55 (PMC2446383; doi:10.1186/1752-0509-2-55)
Supplement: Additional file 1 — SBRT Archive. An archive of the current version of the Systems Biology Research Tool. [file 1752-0509-2-55-S1.zip › sbrt-1.4.0/doc/developers_guide/notes/External_Software_Guidelines.html]

External Software Guidelines - Systems Biology Research
Tool


|  |
| --- |
| > Developer's Guide |
|  |
| External Software Guidelines Support for external software is one of the most powerful features of the Systems Biology Research Tool. It allows skilled software developers to:   |  |  | | --- | --- | | 1. | Create their own SBRT processes (by implementing *process plug-ins*) that can be freely distributed among other SBRT users. | | 2. | Avoid "reinventing the wheel" by utilizing pre-existing software. | | 3. | Encapsulate complicated software packages or libraries as user-friendly SBRT processes, making sophisticated computational techniques accessible to researchers with no programming ability, and for free. |   Nevertheless, developers should proceed with prudence when creating and calling external software. The following guidelines are worth considering before calling external software from your process plug-in.   |  |  | | --- | --- | | 1. | If reasonable, avoid calling external software altogether. This reduces the number of dependencies for your plug-in, which makes installation easier for the end-user. | | 2. | If reasonable, call external software that is already supported by the Systems Biology Research Tool. This also makes installation of your process plug-in easier for the end-user. | | 3. | If reasonable, avoid calling external software written in languages other than Java. This makes your life easier, because you won't have to supply compiled libraries for all the different operating systems and architectures, which is beneficial for the end-user, as well. | | 4. | If reasonable, avoid calling commerical software, because it is usually closed-source and it isn't free. Consequently, it may be impossible to improve its performance if that becomes necessary, and some fraction of the user and developer community will be unable to use your plug-in. | |

  
  
