# Supplementary material for: The Systems Biology Research Tool: evolvable open-source software
Source: BMC Syst Biol. 2008 Jun 29;2:55. doi: 10.1186/1752-0509-2-55 (PMC2446383; doi:10.1186/1752-0509-2-55)
Supplement: Additional file 1 — SBRT Archive. An archive of the current version of the Systems Biology Research Tool. [file 1752-0509-2-55-S1.zip › sbrt-1.4.0/doc/developers_guide/index.html]

Developer's Guide - Systems Biology Research Tool


|  |
| --- |
| The Systems Biology Research Tool  Developer's Guide |
| This document describes the way in which the Systems Biology Research Tool can be used to develop new processes, plugins, and applications. It is intended primarily for experienced software developers.  Here is the API documentation. I recommend reviewing the source code in an integrated development environment.  An example process plug-in (plug\_in\_example.jar) is located in the SBRT's lib directory, and its process name is already present in the provided Process Name File.  Comments, suggestions, or questions can be emailed to Jeremiah Wright at the address: . |

  
  

|  |  |
| --- | --- |
| Notes | Brief Descriptions |
| External Software Guidelines | Guidelines concerning the use of external software by SBRT processes. |
| Kernel-Shell Interactions | Conventions regarding interactions between the kernel and shell. |
