# Supplementary material for: The Systems Biology Research Tool: evolvable open-source software
Source: BMC Syst Biol. 2008 Jun 29;2:55. doi: 10.1186/1752-0509-2-55 (PMC2446383; doi:10.1186/1752-0509-2-55)
Supplement: Additional file 1 — SBRT Archive. An archive of the current version of the Systems Biology Research Tool. [file 1752-0509-2-55-S1.zip › sbrt-1.4.0/doc/developers_guide/api/sbrt/ProcessRunner.html]

ProcessRunner


|  |  |  |  |  |  |  |  |  |  |  |
| --- | --- | --- | --- | --- | --- | --- | --- | --- | --- | --- |
| |  |  |  |  |  |  |  |  | | --- | --- | --- | --- | --- | --- | --- | --- | | **Overview** | **Package** | **Class** | **Use** | **Tree** | **Deprecated** | **Index** | **Help** | | |  |
| **PREV CLASS**   **NEXT CLASS** | **FRAMES**    **NO FRAMES**     **All Classes** |
| SUMMARY: NESTED | FIELD | CONSTR | METHOD | DETAIL: FIELD | CONSTR | METHOD |


---


## sbrt Class ProcessRunner

```
java.lang.Object
  sbrt.ProcessRunner
```

---

``` public class ProcessRunner extends java.lang.Object ```

A class to encapsulate the execution of processes.

**Author:**
:   This class was written and documented by Jeremiah Wright while in the
    Wagner lab.

---

| **Field Summary** | |
| --- | --- |
| `static java.lang.String` | `ERROR_LOG`             The name of the file in which errors will be logged. |


| **Constructor Summary** | |
| --- | --- |
| `ProcessRunner()` |


| **Method Summary** | |
| --- | --- |
| `FatalException` | `interpret(java.lang.Throwable t)`             Interprets the provided throwable. |
| `void` | `run(ManagerInput<java.lang.String,java.lang.String> processInput, java.io.OutputStream out)`             Executes the process specified in the provided process manager input. |
| `void` | `run(ManagerInput<java.lang.String,java.lang.String> processInput, java.io.PrintStream out)`             Executes the process specified in the provided process manager input. |
| `void` | `run(ManagerInput<java.lang.String,java.lang.String> processInput, java.io.PrintWriter out)`             Executes the process specified in the provided process manager input. |
| `void` | `run(java.lang.String processFileName, java.io.OutputStream out)`             Executes the process specified in the indicated process file. |
| `void` | `run(java.lang.String processFileName, java.io.PrintStream out)`             Executes the process specified in the indicated process file. |
| `void` | `run(java.lang.String processFileName, java.io.PrintWriter out)`             Executes the process specified in the indicated process file. |

| **Methods inherited from class java.lang.Object** |
| --- |
| `clone, equals, finalize, getClass, hashCode, notify, notifyAll, toString, wait, wait, wait` |

| **Field Detail** |
| --- |

### ERROR\_LOG

```
public static final java.lang.String ERROR_LOG
```

:   The name of the file in which errors will be logged.

    **See Also:**: Constant Field Values


| **Constructor Detail** |
| --- |

### ProcessRunner

```
public ProcessRunner()
```


| **Method Detail** |
| --- |

### run

```
public void run(java.lang.String processFileName,
                java.io.PrintWriter out)
```

:   Executes the process specified in the indicated process file.

    :   **Parameters:**: `processFileName` - the name of the process file.: `out` - the object to which informative messages will be written.

---


### run

```
public void run(java.lang.String processFileName,
                java.io.OutputStream out)
```

:   Executes the process specified in the indicated process file.

    :   **Parameters:**: `processFileName` - the name of the process file.: `out` - the object to which informative messages will be written.

---


### run

```
public void run(java.lang.String processFileName,
                java.io.PrintStream out)
```

:   Executes the process specified in the indicated process file.

    :   **Parameters:**: `processFileName` - the name of the process file.: `out` - the object to which informative messages will be written.

---


### run

```
public void run(ManagerInput<java.lang.String,java.lang.String> processInput,
                java.io.OutputStream out)
```

:   Executes the process specified in the provided process manager input.

    :   **Parameters:**: `processInput` - the input for the process to be executed.: `out` - the object to which informative messages will be written.

---


### run

```
public void run(ManagerInput<java.lang.String,java.lang.String> processInput,
                java.io.PrintStream out)
```

:   Executes the process specified in the provided process manager input.

    :   **Parameters:**: `processInput` - the input for the process to be executed.: `out` - the object to which informative messages will be written.

---


### run

```
public void run(ManagerInput<java.lang.String,java.lang.String> processInput,
                java.io.PrintWriter out)
```

:   Executes the process specified in the provided process manager input.

    :   **Parameters:**: `processInput` - the input for the process to be executed.: `out` - the object to which informative messages will be written. **Throws:**: `FatalException` - if a fatal error is detected during process execution.

---


### interpret

```
public FatalException interpret(java.lang.Throwable t)
```

:   Interprets the provided throwable.

    :   **Parameters:**: `t` - an object thrown by the SBRT. **Returns:**: an exception whose message describes the error that occurred.


---


|  |  |  |  |  |  |  |  |  |  |  |
| --- | --- | --- | --- | --- | --- | --- | --- | --- | --- | --- |
| |  |  |  |  |  |  |  |  | | --- | --- | --- | --- | --- | --- | --- | --- | | **Overview** | **Package** | **Class** | **Use** | **Tree** | **Deprecated** | **Index** | **Help** | | |  |
| **PREV CLASS**   **NEXT CLASS** | **FRAMES**    **NO FRAMES**     **All Classes** |
| SUMMARY: NESTED | FIELD | CONSTR | METHOD | DETAIL: FIELD | CONSTR | METHOD |


---
