# Supplementary material for: The Systems Biology Research Tool: evolvable open-source software
Source: BMC Syst Biol. 2008 Jun 29;2:55. doi: 10.1186/1752-0509-2-55 (PMC2446383; doi:10.1186/1752-0509-2-55)
Supplement: Additional file 1 — SBRT Archive. An archive of the current version of the Systems Biology Research Tool. [file 1752-0509-2-55-S1.zip › sbrt-1.4.0/doc/developers_guide/api/sbrt/shell/text/SpecialChars.html]

SpecialChars


|  |  |  |  |  |  |  |  |  |  |  |
| --- | --- | --- | --- | --- | --- | --- | --- | --- | --- | --- |
| |  |  |  |  |  |  |  |  | | --- | --- | --- | --- | --- | --- | --- | --- | | **Overview** | **Package** | **Class** | **Use** | **Tree** | **Deprecated** | **Index** | **Help** | | |  |
| **PREV CLASS**   **NEXT CLASS** | **FRAMES**    **NO FRAMES**     **All Classes** |
| SUMMARY: NESTED | FIELD | CONSTR | METHOD | DETAIL: FIELD | CONSTR | METHOD |


---


## sbrt.shell.text Class SpecialChars

```
java.lang.Object
  sbrt.shell.text.SpecialChars
```

---

``` public final class SpecialChars extends java.lang.Object ```

This class contains a set of characters that could be considered as *special*
in some situations.

**Author:**
:   This class was written and documented by
    Jeremiah Wright while in the Wagner lab.

---

| **Field Summary** | |
| --- | --- |
| `static char` | `AMPERSAND`             & |
| `static char` | `ASTERICK`             \* |
| `static char` | `AT`             `@` |
| `static char` | `BACK_SLASH`             \ |
| `static char` | `CARET`             ^ |
| `static char` | `COLON`             : |
| `static char` | `COMMA`             , |
| `static char` | `DASH`             - |
| `static char` | `DOLLAR`             $ |
| `static char` | `DOT`             . |
| `static char` | `EQUALS`             = |
| `static char` | `EXCLAMATION`             ! |
| `static char` | `FORWARD_SLASH`             `/` |
| `static char` | `GREATER_THAN`             > |
| `static char` | `HASH`             # |
| `static char` | `LEFT_BRACE`             { |
| `static char` | `LEFT_BRACKET`             [ |
| `static char` | `LEFT_PAREN`             ( |
| `static char` | `LESS_THAN`             < |
| `static char` | `PERCENT`             % |
| `static char` | `PIPE`             | |
| `static char` | `PLUS`             + |
| `static char` | `QUESTION`             ? |
| `static char` | `RIGHT_BRACE`             { |
| `static char` | `RIGHT_BRACKET`             ] |
| `static char` | `RIGHT_PAREN`             ) |
| `static char` | `SEMICOLON`             ; |
| `static char` | `TILDE`             ~ |


| **Method Summary** | |
| --- | --- |
| `static char[]` | `getArray()`             Returns an array of the characters contained in this class. |
| `static java.util.List<java.lang.Character>` | `getList()`             Returns a list of the characters contained in this class. |
| `static java.util.Set<java.lang.Character>` | `getSet()`             Returns a set of the characters contained in this class. |

| **Methods inherited from class java.lang.Object** |
| --- |
| `clone, equals, finalize, getClass, hashCode, notify, notifyAll, toString, wait, wait, wait` |

| **Field Detail** |
| --- |

### COLON

```
public static final char COLON
```

:   :

    **See Also:**: Constant Field Values

---


### SEMICOLON

```
public static final char SEMICOLON
```

:   ;

    **See Also:**: Constant Field Values

---


### COMMA

```
public static final char COMMA
```

:   ,

    **See Also:**: Constant Field Values

---


### PIPE

```
public static final char PIPE
```

:   |

    **See Also:**: Constant Field Values

---


### HASH

```
public static final char HASH
```

:   #

    **See Also:**: Constant Field Values

---


### TILDE

```
public static final char TILDE
```

:   ~

    **See Also:**: Constant Field Values

---


### ASTERICK

```
public static final char ASTERICK
```

:   \*

    **See Also:**: Constant Field Values

---


### PLUS

```
public static final char PLUS
```

:   +

    **See Also:**: Constant Field Values

---


### EQUALS

```
public static final char EQUALS
```

:   =

    **See Also:**: Constant Field Values

---


### GREATER\_THAN

```
public static final char GREATER_THAN
```

:   >

    **See Also:**: Constant Field Values

---


### LESS\_THAN

```
public static final char LESS_THAN
```

:   <

    **See Also:**: Constant Field Values

---


### LEFT\_PAREN

```
public static final char LEFT_PAREN
```

:   (

    **See Also:**: Constant Field Values

---


### RIGHT\_PAREN

```
public static final char RIGHT_PAREN
```

:   )

    **See Also:**: Constant Field Values

---


### LEFT\_BRACKET

```
public static final char LEFT_BRACKET
```

:   [

    **See Also:**: Constant Field Values

---


### RIGHT\_BRACKET

```
public static final char RIGHT_BRACKET
```

:   ]

    **See Also:**: Constant Field Values

---


### LEFT\_BRACE

```
public static final char LEFT_BRACE
```

:   {

    **See Also:**: Constant Field Values

---


### RIGHT\_BRACE

```
public static final char RIGHT_BRACE
```

:   {

    **See Also:**: Constant Field Values

---


### CARET

```
public static final char CARET
```

:   ^

    **See Also:**: Constant Field Values

---


### AMPERSAND

```
public static final char AMPERSAND
```

:   &

    **See Also:**: Constant Field Values

---


### EXCLAMATION

```
public static final char EXCLAMATION
```

:   !

    **See Also:**: Constant Field Values

---


### AT

```
public static final char AT
```

:   `@`

    **See Also:**: Constant Field Values

---


### DOLLAR

```
public static final char DOLLAR
```

:   $

    **See Also:**: Constant Field Values

---


### PERCENT

```
public static final char PERCENT
```

:   %

    **See Also:**: Constant Field Values

---


### DASH

```
public static final char DASH
```

:   -

    **See Also:**: Constant Field Values

---


### FORWARD\_SLASH

```
public static final char FORWARD_SLASH
```

:   `/`

    **See Also:**: Constant Field Values

---


### BACK\_SLASH

```
public static final char BACK_SLASH
```

:   \

    **See Also:**: Constant Field Values

---


### QUESTION

```
public static final char QUESTION
```

:   ?

    **See Also:**: Constant Field Values

---


### DOT

```
public static final char DOT
```

:   .

    **See Also:**: Constant Field Values


| **Method Detail** |
| --- |

### getArray

```
public static char[] getArray()
```

:   Returns an array of the characters contained in this class.

    :   **Returns:**: an array of the characters contained in this class.

---


### getList

```
public static java.util.List<java.lang.Character> getList()
```

:   Returns a list of the characters contained in this class.

    :   **Returns:**: a list of the characters contained in this class.

---


### getSet

```
public static java.util.Set<java.lang.Character> getSet()
```

:   Returns a set of the characters contained in this class.

    :   **Returns:**: a set of the characters contained in this class.


---


|  |  |  |  |  |  |  |  |  |  |  |
| --- | --- | --- | --- | --- | --- | --- | --- | --- | --- | --- |
| |  |  |  |  |  |  |  |  | | --- | --- | --- | --- | --- | --- | --- | --- | | **Overview** | **Package** | **Class** | **Use** | **Tree** | **Deprecated** | **Index** | **Help** | | |  |
| **PREV CLASS**   **NEXT CLASS** | **FRAMES**    **NO FRAMES**     **All Classes** |
| SUMMARY: NESTED | FIELD | CONSTR | METHOD | DETAIL: FIELD | CONSTR | METHOD |


---
