# Supplementary material for: The Systems Biology Research Tool: evolvable open-source software
Source: BMC Syst Biol. 2008 Jun 29;2:55. doi: 10.1186/1752-0509-2-55 (PMC2446383; doi:10.1186/1752-0509-2-55)
Supplement: Additional file 1 — SBRT Archive. An archive of the current version of the Systems Biology Research Tool. [file 1752-0509-2-55-S1.zip › sbrt-1.4.0/doc/developers_guide/api/sbrt/shell/text/CollectionFormat.html]

CollectionFormat


|  |  |  |  |  |  |  |  |  |  |  |
| --- | --- | --- | --- | --- | --- | --- | --- | --- | --- | --- |
| |  |  |  |  |  |  |  |  | | --- | --- | --- | --- | --- | --- | --- | --- | | **Overview** | **Package** | **Class** | **Use** | **Tree** | **Deprecated** | **Index** | **Help** | | |  |
| **PREV CLASS**   **NEXT CLASS** | **FRAMES**    **NO FRAMES**     **All Classes** |
| SUMMARY: NESTED | FIELD | CONSTR | METHOD | DETAIL: FIELD | CONSTR | METHOD |


---


## sbrt.shell.text Interface CollectionFormat<E>

**Type Parameters:**: `E` - the type of element.

**All Superinterfaces:**: Format, Formatter<java.util.Collection<? extends E>>, Parser<java.util.Collection<? extends E>>, SimpleFormat<java.util.Collection<? extends E>>

---

``` public interface CollectionFormat<E> extends SimpleFormat<java.util.Collection<? extends E>> ```

This interface is used to represent collection formats.

**Author:**
:   This interface was written and documented by
    Jeremiah Wright while in the Wagner lab.

---

| **Method Summary** | |
| --- | --- |
| `java.lang.String` | `format(java.util.Collection<? extends E> c)`             Returns a formatted string representation of the provided collection. |
| `SimpleFormat<E>` | `getElementFormat()`             Returns the format object used for the elements of this collection format. |
| `java.util.Collection<E>` | `parse(java.lang.String source)`             Parses the provided string and returns its corresponding collection of elements. |

| **Methods inherited from interface sbrt.shell.text.Formatter** |
| --- |
| `format` |

| **Method Detail** |
| --- |

### format

```
java.lang.String format(java.util.Collection<? extends E> c)
```

:   Returns a formatted string representation of the
    provided collection.

    :   **Parameters:**: `c` - the collection to be formatted. **Returns:**: a formatted string representation of the provided collection.

---


### parse

```
java.util.Collection<E> parse(java.lang.String source)
```

:   Parses the provided string and returns its
    corresponding collection of elements.

    :   **Specified by:**: `parse` in interface `Parser<java.util.Collection<? extends E>>`
    :   **Parameters:**: `source` - the string to be parsed. **Returns:**: the collection of elements corresponding to the provided string.

---


### getElementFormat

```
SimpleFormat<E> getElementFormat()
```

:   Returns the format object used for
    the elements of this collection format.

    :   **Returns:**: the format object used for the elements of this collection format.


---


|  |  |  |  |  |  |  |  |  |  |  |
| --- | --- | --- | --- | --- | --- | --- | --- | --- | --- | --- |
| |  |  |  |  |  |  |  |  | | --- | --- | --- | --- | --- | --- | --- | --- | | **Overview** | **Package** | **Class** | **Use** | **Tree** | **Deprecated** | **Index** | **Help** | | |  |
| **PREV CLASS**   **NEXT CLASS** | **FRAMES**    **NO FRAMES**     **All Classes** |
| SUMMARY: NESTED | FIELD | CONSTR | METHOD | DETAIL: FIELD | CONSTR | METHOD |


---
