# Supplementary material for: The Systems Biology Research Tool: evolvable open-source software
Source: BMC Syst Biol. 2008 Jun 29;2:55. doi: 10.1186/1752-0509-2-55 (PMC2446383; doi:10.1186/1752-0509-2-55)
Supplement: Additional file 1 — SBRT Archive. An archive of the current version of the Systems Biology Research Tool. [file 1752-0509-2-55-S1.zip › sbrt-1.4.0/doc/developers_guide/api/sbrt/shell/text/LinkedHashSetFormat.html]

LinkedHashSetFormat


|  |  |  |  |  |  |  |  |  |  |  |
| --- | --- | --- | --- | --- | --- | --- | --- | --- | --- | --- |
| |  |  |  |  |  |  |  |  | | --- | --- | --- | --- | --- | --- | --- | --- | | **Overview** | **Package** | **Class** | **Use** | **Tree** | **Deprecated** | **Index** | **Help** | | |  |
| **PREV CLASS**   **NEXT CLASS** | **FRAMES**    **NO FRAMES**     **All Classes** |
| SUMMARY: NESTED | FIELD | CONSTR | METHOD | DETAIL: FIELD | CONSTR | METHOD |


---


## sbrt.shell.text Interface LinkedHashSetFormat<E>

**Type Parameters:**: `E` - the type of element.

**All Superinterfaces:**: Formatter<java.util.Set<? extends E>>, Parser<java.util.LinkedHashSet<E>>

**All Known Implementing Classes:**: AbstractLinkedHashSetFormat, AmpersandSetFormat, PipeSetFormat, TabSetFormat, WhitespaceSetFormat

---

``` public interface LinkedHashSetFormat<E> extends Formatter<java.util.Set<? extends E>>, Parser<java.util.LinkedHashSet<E>> ```

This interface is used to represent `LinkedHashSet`
formats. Ideally, this interface would represent the format
of ordered sets, without reference to their implementation
details; but unfortunately, the only type of ordered set
in Java is the `LinkedHashSet`.

**Author:**
:   This interface was written and documented by
    Jeremiah Wright while in the Wagner lab.

---

| **Method Summary** | |
| --- | --- |
| `java.lang.String` | `format(java.util.Set<? extends E> set)`             Returns a formatted string representation of the provided set. |
| `Formatter<E>` | `getElementFormatter()`             Returns the formatter for the elements of this set format. |
| `Parser<E>` | `getElementParser()`             Returns the parser for the elements of this set format. |
| `java.util.LinkedHashSet<E>` | `parse(java.lang.String set)`             Parses the provided string and returns its corresponding collection of elements as a `LinkedHashSet`. |

| **Methods inherited from interface sbrt.shell.text.Formatter** |
| --- |
| `format` |

| **Method Detail** |
| --- |

### format

```
java.lang.String format(java.util.Set<? extends E> set)
```

:   Returns a formatted string representation of the
    provided set.

    :   **Parameters:**: `set` - the set to be formatted. **Returns:**: a formatted string representation of the provided set.

---


### parse

```
java.util.LinkedHashSet<E> parse(java.lang.String set)
```

:   Parses the provided string and returns its
    corresponding collection of elements as a
    `LinkedHashSet`.

    :   **Specified by:**: `parse` in interface `Parser<java.util.LinkedHashSet<E>>`
    :   **Parameters:**: `set` - the string to be parsed. **Returns:**: the set of elements corresponding to the provided string.

---


### getElementFormatter

```
Formatter<E> getElementFormatter()
```

:   Returns the formatter for
    the elements of this set format.

    :   **Returns:**: the formatter for the elements of this set format.

---


### getElementParser

```
Parser<E> getElementParser()
```

:   Returns the parser for
    the elements of this set format.

    :   **Returns:**: the parser for the elements of this set format.


---


|  |  |  |  |  |  |  |  |  |  |  |
| --- | --- | --- | --- | --- | --- | --- | --- | --- | --- | --- |
| |  |  |  |  |  |  |  |  | | --- | --- | --- | --- | --- | --- | --- | --- | | **Overview** | **Package** | **Class** | **Use** | **Tree** | **Deprecated** | **Index** | **Help** | | |  |
| **PREV CLASS**   **NEXT CLASS** | **FRAMES**    **NO FRAMES**     **All Classes** |
| SUMMARY: NESTED | FIELD | CONSTR | METHOD | DETAIL: FIELD | CONSTR | METHOD |


---
