# Supplementary material for: The Systems Biology Research Tool: evolvable open-source software
Source: BMC Syst Biol. 2008 Jun 29;2:55. doi: 10.1186/1752-0509-2-55 (PMC2446383; doi:10.1186/1752-0509-2-55)
Supplement: Additional file 1 — SBRT Archive. An archive of the current version of the Systems Biology Research Tool. [file 1752-0509-2-55-S1.zip › sbrt-1.4.0/doc/developers_guide/api/sbrt/shell/text/AbstractMapFormat.html]

AbstractMapFormat


|  |  |  |  |  |  |  |  |  |  |  |
| --- | --- | --- | --- | --- | --- | --- | --- | --- | --- | --- |
| |  |  |  |  |  |  |  |  | | --- | --- | --- | --- | --- | --- | --- | --- | | **Overview** | **Package** | **Class** | **Use** | **Tree** | **Deprecated** | **Index** | **Help** | | |  |
| **PREV CLASS**   **NEXT CLASS** | **FRAMES**    **NO FRAMES**     **All Classes** |
| SUMMARY: NESTED | FIELD | CONSTR | METHOD | DETAIL: FIELD | CONSTR | METHOD |


---


## sbrt.shell.text Class AbstractMapFormat<K,V>

```
java.lang.Object
  sbrt.shell.text.AbstractMapFormat<K,V>
```

**Type Parameters:**: `K` - the key type.: `V` - the value type.

**All Implemented Interfaces:**: Format, MapFormat<K,V>

**Direct Known Subclasses:**: AppInputFileLineFormatVersion1, ColonMapFormatV1, ConstraintsFileLineFormatV1, ConstraintsFileLineFormatV2, EqualsMapFormatV1, FluxCapFormatV1, FluxomeSolFileLineFormatV1, FluxVectorFormatV1, IrfFormatV1

---

``` public abstract class AbstractMapFormat<K,V> extends java.lang.Object implements MapFormat<K,V> ```

This abstract class is a skeleton implementation of
`MapFormat`.

**Author:**
:   This class was written and documented by
    Jeremiah Wright while in the Wagner lab.

---

| **Constructor Summary** | |
| --- | --- |
| `AbstractMapFormat()` |


| **Method Summary** | |
| --- | --- |
| `|  | | --- | | <S extends K>   java.lang.String |` | `formatKey(S key)`             Returns a formatted string representation of the provided key. |
| `java.lang.String` | `formatKeys(java.util.Set<? extends K> keys)`             Returns a formatted string representation of the provided set of keys. |
| `java.lang.String` | `formatPair(java.util.Map.Entry<? extends K,? extends V> entry)`             Returns a formatted string representation of the provided map entry. |
| `|  | | --- | | <S extends K,T extends V>   java.lang.String |` | `formatPair(S key, T value)`             Returns a string representation of the provided key-value pair. |
| `|  | | --- | | <T extends V>   java.lang.String |` | `formatValue(T value)`             Returns a formatted string representation of the provided value. |
| `java.lang.String` | `formatValues(java.util.List<? extends V> values)`             Returns a string representation of the provided list of values. |
| `java.lang.String` | `formatValues(java.util.Map<? extends K,? extends V> map)`             Returns a string representation of the values in the provided map formatted as a list. |
| `Formatter<K>` | `getKeyFormatter()`             Returns the formatter used for keys. |
| `Parser<K>` | `getKeyParser()`             Returns the parser used for keys. |
| `abstract  ListFormat<V>` | `getListFormat()`             Returns the format used for lists of values. |
| `abstract  MapEntryFormat<java.lang.String,java.lang.String>` | `getMapEntryFormat()`             Returns the format used for variable-value pairs. |
| `abstract  LinkedHashSetFormat<K>` | `getSetFormat()`             Returns the format used for sets of keys. |
| `Formatter<V>` | `getValueFormatter()`             Returns the formatter used for values. |
| `Parser<V>` | `getValueParser()`             Returns the parser used for values. |
| `java.util.LinkedHashSet<K>` | `parseAllKeys(java.lang.String allKeys)`             Parses the provided string and returns its corresponding set of keys. |
| `java.util.Map<K,V>` | `parseAllPairs(java.lang.String allkeys, java.lang.String allValues)`             Parses the provided set of keys and list of values and returns a corresponding map of variable-value pairs. |
| `java.util.Map<K,V>` | `parseAllValues(java.util.LinkedHashSet<? extends K> allkeys, java.lang.String allValues)`             Parses the provided string of values and returns a corresponding map of variable-value pairs using the provided set of keys. |
| `java.util.List<V>` | `parseAllValues(java.lang.String allValues)`             Parses the provided string and returns its corresponding list of values. |
| `K` | `parseKey(java.lang.String key)`             Parses the provided string and returns its corresponding key. |
| `java.util.Map.Entry<K,V>` | `parsePair(java.lang.String mapEntry)`             Parses the provided map entry string, and returns its corresponding map entry. |
| `V` | `parseValue(java.lang.String value)`             Parses the provided string and returns its corresponding value. |

| **Methods inherited from class java.lang.Object** |
| --- |
| `clone, equals, finalize, getClass, hashCode, notify, notifyAll, toString, wait, wait, wait` |

| **Constructor Detail** |
| --- |

### AbstractMapFormat

```
public AbstractMapFormat()
```


| **Method Detail** |
| --- |

### getKeyFormatter

```
public Formatter<K> getKeyFormatter()
```

:   Returns the formatter used for keys.

    :   **Returns:**: the formatter used for keys.

---


### getKeyParser

```
public Parser<K> getKeyParser()
```

:   Returns the parser used for keys.

    :   **Returns:**: the parser used for keys.

---


### getValueFormatter

```
public Formatter<V> getValueFormatter()
```

:   Returns the formatter used for values.

    :   **Returns:**: the formatter used for values.

---


### getValueParser

```
public Parser<V> getValueParser()
```

:   Returns the parser used for values.

    :   **Returns:**: the parser used for values.

---


### getMapEntryFormat

```
public abstract MapEntryFormat<java.lang.String,java.lang.String> getMapEntryFormat()
```

:   Returns the format used for variable-value pairs. This
    format must be a basic format, this is, one that does not
    format or parse the keys or values supplied to it.

    :   **Returns:**: the format used for variable-value pairs.

---


### getSetFormat

```
public abstract LinkedHashSetFormat<K> getSetFormat()
```

:   Returns the format used for sets of keys.

    :   **Returns:**: the format used for sets of keys.

---


### getListFormat

```
public abstract ListFormat<V> getListFormat()
```

:   Returns the format used for lists of values.

    :   **Returns:**: the format used for lists of values.

---


### formatKey

```
public <S extends K> java.lang.String formatKey(S key)
```

:   Returns a formatted string representation of the
    provided key.

    :   **Specified by:**: `formatKey` in interface `MapFormat<K,V>`
    :   **Type Parameters:**: `S` - a type that extends the primary key type of this format. **Parameters:**: `key` - the key to be formatted. **Returns:**: a formatted string representation of the provided key.

---


### formatValue

```
public <T extends V> java.lang.String formatValue(T value)
```

:   Returns a formatted string representation of the
    provided value.

    :   **Specified by:**: `formatValue` in interface `MapFormat<K,V>`
    :   **Type Parameters:**: `T` - a type that extends the primary value type of this format. **Parameters:**: `value` - the value to be formatted. **Returns:**: a formatted string representation of the provided value.

---


### formatKeys

```
public java.lang.String formatKeys(java.util.Set<? extends K> keys)
```

:   Returns a formatted string representation of the
    provided set of keys.

    :   **Specified by:**: `formatKeys` in interface `MapFormat<K,V>`
    :   **Parameters:**: `keys` - the set of keys to be formatted. **Returns:**: a formatted string representation of the provided set of keys.

---


### formatValues

```
public java.lang.String formatValues(java.util.List<? extends V> values)
```

:   Returns a string representation of the
    provided list of values.

    :   **Specified by:**: `formatValues` in interface `MapFormat<K,V>`
    :   **Parameters:**: `values` - the list of values to be formatted. **Returns:**: a formatted string representation of the provided list of values.

---


### formatValues

```
public java.lang.String formatValues(java.util.Map<? extends K,? extends V> map)
```

:   Returns a string representation of the
    values in the provided map formatted as a list. The order of
    occurrence of values in the returned string depends
    on the *order* of the map. See
    `java.util.Map` for further details.

    :   **Specified by:**: `formatValues` in interface `MapFormat<K,V>`
    :   **Parameters:**: `map` - keys mapped to their respective values. **Returns:**: a string representation of the values in the provided map formatted as a list.

---


### formatPair

```
public <S extends K,T extends V> java.lang.String formatPair(S key,
                                                             T value)
```

:   Returns a string representation of the provided
    key-value pair.

    :   **Specified by:**: `formatPair` in interface `MapFormat<K,V>`
    :   **Type Parameters:**: `S` - a type extending the primary key type.: `T` - a type extending the primary value type. **Parameters:**: `key` - the key.: `value` - the value. **Returns:**: a string representation of the provided key-value pair.

---


### formatPair

```
public java.lang.String formatPair(java.util.Map.Entry<? extends K,? extends V> entry)
```

:   Returns a formatted string representation of the
    provided map entry.

    :   **Specified by:**: `formatPair` in interface `MapFormat<K,V>`
    :   **Parameters:**: `entry` - the map entry. **Returns:**: a formatted string representation of the provided map entry.

---


### parseKey

```
public K parseKey(java.lang.String key)
```

:   Parses the provided string and returns its
    corresponding key.

    :   **Specified by:**: `parseKey` in interface `MapFormat<K,V>`
    :   **Parameters:**: `key` - a string representation of a key. **Returns:**: the key corresponding to the provided string.

---


### parseValue

```
public V parseValue(java.lang.String value)
```

:   Parses the provided string and returns its
    corresponding value.

    :   **Specified by:**: `parseValue` in interface `MapFormat<K,V>`
    :   **Parameters:**: `value` - a string representation of a value. **Returns:**: the value corresponding to the provided string.

---


### parsePair

```
public java.util.Map.Entry<K,V> parsePair(java.lang.String mapEntry)
```

:   Parses the provided map entry string, and
    returns its corresponding map entry.

    :   **Specified by:**: `parsePair` in interface `MapFormat<K,V>`
    :   **Parameters:**: `mapEntry` - the string representation of a single map entry. **Returns:**: a singleton map corresponding to the provided string.

---


### parseAllKeys

```
public java.util.LinkedHashSet<K> parseAllKeys(java.lang.String allKeys)
```

:   Parses the provided string and returns its
    corresponding set of keys.

    :   **Specified by:**: `parseAllKeys` in interface `MapFormat<K,V>`
    :   **Parameters:**: `allKeys` - a string representation of a set of keys. **Returns:**: a set of keys corresponding to the provided string.

---


### parseAllValues

```
public java.util.List<V> parseAllValues(java.lang.String allValues)
```

:   Parses the provided string and returns its
    corresponding list of values.

    :   **Specified by:**: `parseAllValues` in interface `MapFormat<K,V>`
    :   **Parameters:**: `allValues` - a string representation of a list of values. **Returns:**: a list of values corresponding to the provided string.

---


### parseAllValues

```
public java.util.Map<K,V> parseAllValues(java.util.LinkedHashSet<? extends K> allkeys,
                                         java.lang.String allValues)
```

:   Parses the provided string of values and returns a
    corresponding map of variable-value pairs using the
    provided set of keys. The association between
    keys and values is determined from their order of
    occurrence in their respective collection. In other
    words, the first variable returned by the set's iterator
    is associated with the first parsed value, and so on.

    :   **Specified by:**: `parseAllValues` in interface `MapFormat<K,V>`
    :   **Parameters:**: `allkeys` - the set of keys with which to associate the parsed values.: `allValues` - a string representation of a list of values. **Returns:**: keys mapped to their respective values.

---


### parseAllPairs

```
public java.util.Map<K,V> parseAllPairs(java.lang.String allkeys,
                                        java.lang.String allValues)
```

:   Parses the provided set of keys and list of values
    and returns a corresponding map of variable-value pairs.
    The association between keys and values is determined
    from their order of occurrence in their respective collection.
    In other words, the first parsed variable is
    associated with the first parsed value, and so on.

    :   **Specified by:**: `parseAllPairs` in interface `MapFormat<K,V>`
    :   **Parameters:**: `allkeys` - a string representation of a set of keys.: `allValues` - a string representation of a list of values. **Returns:**: keys mapped to their respective values.


---


|  |  |  |  |  |  |  |  |  |  |  |
| --- | --- | --- | --- | --- | --- | --- | --- | --- | --- | --- |
| |  |  |  |  |  |  |  |  | | --- | --- | --- | --- | --- | --- | --- | --- | | **Overview** | **Package** | **Class** | **Use** | **Tree** | **Deprecated** | **Index** | **Help** | | |  |
| **PREV CLASS**   **NEXT CLASS** | **FRAMES**    **NO FRAMES**     **All Classes** |
| SUMMARY: NESTED | FIELD | CONSTR | METHOD | DETAIL: FIELD | CONSTR | METHOD |


---
