# Supplementary material for: The Systems Biology Research Tool: evolvable open-source software
Source: BMC Syst Biol. 2008 Jun 29;2:55. doi: 10.1186/1752-0509-2-55 (PMC2446383; doi:10.1186/1752-0509-2-55)
Supplement: Additional file 1 — SBRT Archive. An archive of the current version of the Systems Biology Research Tool. [file 1752-0509-2-55-S1.zip › sbrt-1.4.0/doc/developers_guide/api/sbrt/shell/text/WhitespaceSetFormat.html]

WhitespaceSetFormat


|  |  |  |  |  |  |  |  |  |  |  |
| --- | --- | --- | --- | --- | --- | --- | --- | --- | --- | --- |
| |  |  |  |  |  |  |  |  | | --- | --- | --- | --- | --- | --- | --- | --- | | **Overview** | **Package** | **Class** | **Use** | **Tree** | **Deprecated** | **Index** | **Help** | | |  |
| **PREV CLASS**   **NEXT CLASS** | **FRAMES**    **NO FRAMES**     **All Classes** |
| SUMMARY: NESTED | FIELD | CONSTR | METHOD | DETAIL: FIELD | CONSTR | METHOD |


---


## sbrt.shell.text Class WhitespaceSetFormat<E>

```
java.lang.Object
  sbrt.shell.text.AbstractLinkedHashSetFormat<E>
      sbrt.shell.text.WhitespaceSetFormat<E>
```

**Type Parameters:**: `E` - the element type.

**All Implemented Interfaces:**: Formatter<java.util.Set<? extends E>>, LinkedHashSetFormat<E>, Parser<java.util.LinkedHashSet<E>>

---

``` public final class WhitespaceSetFormat<E> extends AbstractLinkedHashSetFormat<E> ```

This class is used to define a format for whitespace-delimited
sets. Whitespace characters include: spaces, tabs, and new line
characters. If a set is composed of elements
*a, b, c*,
its string representation would be
"`a b c`".

**Author:**
:   This class was written and documented by
    Jeremiah Wright while in the Wagner lab.

---

| **Constructor Summary** | |
| --- | --- |
| `WhitespaceSetFormat(Formatter<E> elementFormatter, Parser<E> elementParser)`             Constructs a new whitespace-delimited set format with the provided element format. |
| `WhitespaceSetFormat(SimpleFormat<E> elementFormat)`             Constructs a new whitespace-delimited set format with the provided element format. |


| **Method Summary** | |
| --- | --- |
| `static WhitespaceSetFormat<java.lang.String>` | `getBasic()`             Returns a `WhitespaceSetFormat` that does not format or parse the elements supplied to it. |

| **Methods inherited from class sbrt.shell.text.AbstractLinkedHashSetFormat** |
| --- |
| `format, getDelimiter, getElementFormatter, getElementParser, getRegex, parse` |

| **Methods inherited from class java.lang.Object** |
| --- |
| `clone, equals, finalize, getClass, hashCode, notify, notifyAll, toString, wait, wait, wait` |

| **Methods inherited from interface sbrt.shell.text.Formatter** |
| --- |
| `format` |

| **Constructor Detail** |
| --- |

### WhitespaceSetFormat

```
public WhitespaceSetFormat(SimpleFormat<E> elementFormat)
```

:   Constructs a new whitespace-delimited set format with the
    provided element format.

    **Parameters:**: `elementFormat` - the format of the set-elements.

---


### WhitespaceSetFormat

```
public WhitespaceSetFormat(Formatter<E> elementFormatter,
                           Parser<E> elementParser)
```

:   Constructs a new whitespace-delimited set format with the
    provided element format.

    **Parameters:**: `elementFormatter` - the formatter for the set-elements.: `elementParser` - the parser for the set-elements.


| **Method Detail** |
| --- |

### getBasic

```
public static final WhitespaceSetFormat<java.lang.String> getBasic()
```

:   Returns a `WhitespaceSetFormat` that does not
    format or parse the elements supplied to it. In other
    words, it returns a `WhitespaceSetFormat` with
    the most basic possible functionality. Empty strings are not
    allowed however.

    :   **Returns:**: an `WhitespaceSetFormat` that does not format or parse the elements supplied to it.


---


|  |  |  |  |  |  |  |  |  |  |  |
| --- | --- | --- | --- | --- | --- | --- | --- | --- | --- | --- |
| |  |  |  |  |  |  |  |  | | --- | --- | --- | --- | --- | --- | --- | --- | | **Overview** | **Package** | **Class** | **Use** | **Tree** | **Deprecated** | **Index** | **Help** | | |  |
| **PREV CLASS**   **NEXT CLASS** | **FRAMES**    **NO FRAMES**     **All Classes** |
| SUMMARY: NESTED | FIELD | CONSTR | METHOD | DETAIL: FIELD | CONSTR | METHOD |


---
