# Supplementary material for: The Systems Biology Research Tool: evolvable open-source software
Source: BMC Syst Biol. 2008 Jun 29;2:55. doi: 10.1186/1752-0509-2-55 (PMC2446383; doi:10.1186/1752-0509-2-55)
Supplement: Additional file 1 — SBRT Archive. An archive of the current version of the Systems Biology Research Tool. [file 1752-0509-2-55-S1.zip › sbrt-1.4.0/doc/developers_guide/api/sbrt/shell/text/IntegerFormatV1.html]

IntegerFormatV1


|  |  |  |  |  |  |  |  |  |  |  |
| --- | --- | --- | --- | --- | --- | --- | --- | --- | --- | --- |
| |  |  |  |  |  |  |  |  | | --- | --- | --- | --- | --- | --- | --- | --- | | **Overview** | **Package** | **Class** | **Use** | **Tree** | **Deprecated** | **Index** | **Help** | | |  |
| **PREV CLASS**   **NEXT CLASS** | **FRAMES**    **NO FRAMES**     **All Classes** |
| SUMMARY: NESTED | FIELD | CONSTR | METHOD | DETAIL: FIELD | CONSTR | METHOD |


---


## sbrt.shell.text Class IntegerFormatV1

```
java.lang.Object
  sbrt.shell.text.IntegerFormatV1
```

**All Implemented Interfaces:**: Format, Formatter<java.lang.Integer>, IntegerFormat, Parser<java.lang.Integer>, SimpleFormat<java.lang.Integer>

---

``` public class IntegerFormatV1 extends java.lang.Object implements IntegerFormat ```

This class is a concrete implementation of `IntegerFormat`.

**Author:**
:   This class was written and documented by
    Jeremiah Wright while in the Wagner lab.

---

| **Constructor Summary** | |
| --- | --- |
| `IntegerFormatV1()` |


| **Method Summary** | |
| --- | --- |
| `java.lang.String` | `format(int i)`             Parses the provided string and returns its corresponding integer. |
| `java.lang.String` | `format(java.lang.Integer i)`             Returns a formatted string representation of the provided integer. |
| `java.lang.Integer` | `parse(java.lang.String i)`             Parses the provided string and returns its corresponding integer. |
| `int` | `parsePrimitive(java.lang.String i)`             Parses the provided string and returns its corresponding integer. |

| **Methods inherited from class java.lang.Object** |
| --- |
| `clone, equals, finalize, getClass, hashCode, notify, notifyAll, toString, wait, wait, wait` |

| **Constructor Detail** |
| --- |

### IntegerFormatV1

```
public IntegerFormatV1()
```


| **Method Detail** |
| --- |

### format

```
public java.lang.String format(java.lang.Integer i)
```

:   Returns a formatted string representation of the
    provided integer. See
    Integer.toString(int) for the
    exact formatting rules.

    :   **Specified by:**: `format` in interface `Formatter<java.lang.Integer>` **Specified by:**: `format` in interface `IntegerFormat`
    :   **Parameters:**: `i` - the integer to be formatted. **Returns:**: a formatted string representation of the provided integer.

---


### format

```
public java.lang.String format(int i)
```

:   Parses the provided string and returns its
    corresponding integer. See
    Integer.valueOf(String)
    for the exact formatting rules.

    :   **Specified by:**: `format` in interface `IntegerFormat`
    :   **Parameters:**: `i` - the string to be parsed. **Returns:**: the integer corresponding to the provided string. **Throws:**: `FormatException` - if the provided string is not parsable as a integer.: `java.lang.NullPointerException` - if the provided argument is `null`.

---


### parse

```
public java.lang.Integer parse(java.lang.String i)
```

:   Parses the provided string and returns its
    corresponding integer. See
    Integer.valueOf(String)
    for the exact formatting rules.

    :   **Specified by:**: `parse` in interface `IntegerFormat` **Specified by:**: `parse` in interface `Parser<java.lang.Integer>`
    :   **Parameters:**: `i` - the string to be parsed. **Returns:**: the integer corresponding to the provided string. **Throws:**: `FormatException` - if the provided string is not parsable as a integer.: `java.lang.NullPointerException` - if the provided argument is `null`.

---


### parsePrimitive

```
public int parsePrimitive(java.lang.String i)
```

:   Parses the provided string and returns its
    corresponding integer. See
    Integer.valueOf(String)
    for the exact formatting rules.

    :   **Specified by:**: `parsePrimitive` in interface `IntegerFormat`
    :   **Parameters:**: `i` - the string to be parsed. **Returns:**: the integer corresponding to the provided string. **Throws:**: `FormatException` - if the provided string is not parsable as a integer.: `java.lang.NullPointerException` - if the provided argument is `null`.


---


|  |  |  |  |  |  |  |  |  |  |  |
| --- | --- | --- | --- | --- | --- | --- | --- | --- | --- | --- |
| |  |  |  |  |  |  |  |  | | --- | --- | --- | --- | --- | --- | --- | --- | | **Overview** | **Package** | **Class** | **Use** | **Tree** | **Deprecated** | **Index** | **Help** | | |  |
| **PREV CLASS**   **NEXT CLASS** | **FRAMES**    **NO FRAMES**     **All Classes** |
| SUMMARY: NESTED | FIELD | CONSTR | METHOD | DETAIL: FIELD | CONSTR | METHOD |


---
