# Supplementary material for: The Systems Biology Research Tool: evolvable open-source software
Source: BMC Syst Biol. 2008 Jun 29;2:55. doi: 10.1186/1752-0509-2-55 (PMC2446383; doi:10.1186/1752-0509-2-55)
Supplement: Additional file 1 — SBRT Archive. An archive of the current version of the Systems Biology Research Tool. [file 1752-0509-2-55-S1.zip › sbrt-1.4.0/doc/developers_guide/api/sbrt/shell/text/fba/ChemSpeciesVerifier.html]

ChemSpeciesVerifier


|  |  |  |  |  |  |  |  |  |  |  |
| --- | --- | --- | --- | --- | --- | --- | --- | --- | --- | --- |
| |  |  |  |  |  |  |  |  | | --- | --- | --- | --- | --- | --- | --- | --- | | **Overview** | **Package** | **Class** | **Use** | **Tree** | **Deprecated** | **Index** | **Help** | | |  |
| **PREV CLASS**   **NEXT CLASS** | **FRAMES**    **NO FRAMES**     **All Classes** |
| SUMMARY: NESTED | FIELD | CONSTR | METHOD | DETAIL: FIELD | CONSTR | METHOD |


---


## sbrt.shell.text.fba Class ChemSpeciesVerifier

```
java.lang.Object
  sbrt.shell.text.fba.ChemSpeciesVerifier
```

**All Implemented Interfaces:**: ChemSpeciesFormat, Format, Formatter<java.lang.String>, Parser<java.lang.String>, SimpleFormat<java.lang.String>

---

``` public class ChemSpeciesVerifier extends java.lang.Object implements ChemSpeciesFormat ```

This class is used to ensure chemical species exist in a
given `Fluxome`.

**Author:**
:   This class was written and documented by
    Jeremiah Wright while in the Wagner lab.

---

| **Constructor Summary** | |
| --- | --- |
| `ChemSpeciesVerifier(Fluxome fluxome)`             Constructs a new chemical species verifier for the provided fluxome. |


| **Method Summary** | |
| --- | --- |
| `java.lang.String` | `format(java.lang.String validChemSpecies)`             Returns the provided chemical species after ensuring it exists in the corresponding fluxome of this chemical species verifier. |
| `Fluxome` | `getFluxome()`             Returns the fluxome used to create this chemical species verifier. |
| `java.lang.String` | `parse(java.lang.String sourceChemSpecies)`             Parses the provided string and returns its corresponding chemical species. |

| **Methods inherited from class java.lang.Object** |
| --- |
| `clone, equals, finalize, getClass, hashCode, notify, notifyAll, toString, wait, wait, wait` |

| **Constructor Detail** |
| --- |

### ChemSpeciesVerifier

```
public ChemSpeciesVerifier(Fluxome fluxome)
```

:   Constructs a new chemical species verifier for the
    provided fluxome.

    **Parameters:**: `fluxome` - the fluxome whose chemical species will be used for comparison.


| **Method Detail** |
| --- |

### getFluxome

```
public Fluxome getFluxome()
```

:   Returns the fluxome used to create this chemical species verifier.

    :   **Returns:**: the fluxome used to create this chemical species verifier.

---


### format

```
public java.lang.String format(java.lang.String validChemSpecies)
```

:   Returns the provided chemical species after ensuring it
    exists in the corresponding fluxome of this chemical species verifier.

    :   **Specified by:**: `format` in interface `Formatter<java.lang.String>`
    :   **Parameters:**: `validChemSpecies` - the name of a chemical species in this metabolite name checker's fluxome. **Returns:**: the provided chemical species after ensuring it exists in the corresponding fluxome of this chemical species verifier. **Throws:**: `java.lang.IllegalArgumentException` - if the provided string is not the name of a chemical species in this chemical species verifier's fluxome.

---


### parse

```
public java.lang.String parse(java.lang.String sourceChemSpecies)
```

:   Parses the provided string and returns its
    corresponding chemical species.

    :   **Specified by:**: `parse` in interface `Parser<java.lang.String>`
    :   **Parameters:**: `sourceChemSpecies` - the string to be parsed. **Returns:**: a copy of the provided string with leading and trailing white space removed, or the provided string if it has no leading or trailing white space. **Throws:**: `FormatException` - if the provided string is not a valid chemical species in the fluxome used to create this chemical species verifier.: `java.lang.NullPointerException` - if the provided string is `null`.


---


|  |  |  |  |  |  |  |  |  |  |  |
| --- | --- | --- | --- | --- | --- | --- | --- | --- | --- | --- |
| |  |  |  |  |  |  |  |  | | --- | --- | --- | --- | --- | --- | --- | --- | | **Overview** | **Package** | **Class** | **Use** | **Tree** | **Deprecated** | **Index** | **Help** | | |  |
| **PREV CLASS**   **NEXT CLASS** | **FRAMES**    **NO FRAMES**     **All Classes** |
| SUMMARY: NESTED | FIELD | CONSTR | METHOD | DETAIL: FIELD | CONSTR | METHOD |


---
