# Supplementary material for: The Systems Biology Research Tool: evolvable open-source software
Source: BMC Syst Biol. 2008 Jun 29;2:55. doi: 10.1186/1752-0509-2-55 (PMC2446383; doi:10.1186/1752-0509-2-55)
Supplement: Additional file 1 — SBRT Archive. An archive of the current version of the Systems Biology Research Tool. [file 1752-0509-2-55-S1.zip › sbrt-1.4.0/doc/developers_guide/api/sbrt/shell/text/fba/CatalystVerifier.html]

CatalystVerifier


|  |  |  |  |  |  |  |  |  |  |  |
| --- | --- | --- | --- | --- | --- | --- | --- | --- | --- | --- |
| |  |  |  |  |  |  |  |  | | --- | --- | --- | --- | --- | --- | --- | --- | | **Overview** | **Package** | **Class** | **Use** | **Tree** | **Deprecated** | **Index** | **Help** | | |  |
| **PREV CLASS**   **NEXT CLASS** | **FRAMES**    **NO FRAMES**     **All Classes** |
| SUMMARY: NESTED | FIELD | CONSTR | METHOD | DETAIL: FIELD | CONSTR | METHOD |


---


## sbrt.shell.text.fba Class CatalystVerifier

```
java.lang.Object
  sbrt.shell.text.fba.CatalystVerifier
```

**All Implemented Interfaces:**: CatalystFormat, Format, Formatter<java.lang.String>, Parser<java.lang.String>, SimpleFormat<java.lang.String>

---

``` public class CatalystVerifier extends java.lang.Object implements CatalystFormat ```

This class is used to ensure catalysts exist in a given `CatalyzedFluxome`.

**Author:**
:   This class was written and documented by
    Jeremiah Wright while in the Wagner lab.

---

| **Constructor Summary** | |
| --- | --- |
| `CatalystVerifier(CatalyzedFluxome fluxome)`             Constructs a new catalyst name verifier for the provided fluxome. |


| **Method Summary** | |
| --- | --- |
| `java.lang.String` | `format(java.lang.String catalyst)`             Returns the provided catalyst name after ensuring it exists in the corresponding fluxome of this catalyst name verifier. |
| `CatalyzedFluxome` | `getFluxome()`             Returns the fluxome used to create this catalyst name verifier. |
| `java.lang.String` | `parse(java.lang.String catalystString)`             Parses the provided string and returns its corresponding catalyst name. |

| **Methods inherited from class java.lang.Object** |
| --- |
| `clone, equals, finalize, getClass, hashCode, notify, notifyAll, toString, wait, wait, wait` |

| **Constructor Detail** |
| --- |

### CatalystVerifier

```
public CatalystVerifier(CatalyzedFluxome fluxome)
```

:   Constructs a new catalyst name verifier for the provided fluxome.

    **Parameters:**: `fluxome` - the fluxome whose catalysts will be used for comparison.


| **Method Detail** |
| --- |

### getFluxome

```
public CatalyzedFluxome getFluxome()
```

:   Returns the fluxome used to create this catalyst name verifier.

    :   **Returns:**: the fluxome used to create this catalyst name verifier.

---


### format

```
public java.lang.String format(java.lang.String catalyst)
```

:   Returns the provided catalyst name after ensuring it
    exists in the corresponding fluxome of this catalyst name verifier.

    :   **Specified by:**: `format` in interface `Formatter<java.lang.String>`
    :   **Parameters:**: `catalyst` - the name of a catalyst in this catalyst name verifier's fluxome. **Returns:**: the provided catalyst name. **Throws:**: `java.lang.IllegalArgumentException` - if the provided string is not the name of a catalyst in this catalyst name verifier's fluxome.

---


### parse

```
public java.lang.String parse(java.lang.String catalystString)
```

:   Parses the provided string and returns its
    corresponding catalyst name.

    :   **Specified by:**: `parse` in interface `Parser<java.lang.String>`
    :   **Parameters:**: `catalystString` - the string to be parsed. **Returns:**: a copy of the provided string with leading and trailing white space removed, or the provided string if it has no leading or trailing white space. **Throws:**: `FormatException` - if the provided string is not a valid catalyst name in the fluxome used to create this catalyst name verifier.: `java.lang.NullPointerException` - if the provided string is `null`.


---


|  |  |  |  |  |  |  |  |  |  |  |
| --- | --- | --- | --- | --- | --- | --- | --- | --- | --- | --- |
| |  |  |  |  |  |  |  |  | | --- | --- | --- | --- | --- | --- | --- | --- | | **Overview** | **Package** | **Class** | **Use** | **Tree** | **Deprecated** | **Index** | **Help** | | |  |
| **PREV CLASS**   **NEXT CLASS** | **FRAMES**    **NO FRAMES**     **All Classes** |
| SUMMARY: NESTED | FIELD | CONSTR | METHOD | DETAIL: FIELD | CONSTR | METHOD |


---
