# Supplementary material for: The Systems Biology Research Tool: evolvable open-source software
Source: BMC Syst Biol. 2008 Jun 29;2:55. doi: 10.1186/1752-0509-2-55 (PMC2446383; doi:10.1186/1752-0509-2-55)
Supplement: Additional file 1 — SBRT Archive. An archive of the current version of the Systems Biology Research Tool. [file 1752-0509-2-55-S1.zip › sbrt-1.4.0/doc/developers_guide/api/sbrt/shell/text/fba/package-summary.html]

sbrt.shell.text.fba


|  |  |  |  |  |  |  |  |  |  |  |
| --- | --- | --- | --- | --- | --- | --- | --- | --- | --- | --- |
| |  |  |  |  |  |  |  |  | | --- | --- | --- | --- | --- | --- | --- | --- | | **Overview** | **Package** | Class | **Use** | **Tree** | **Deprecated** | **Index** | **Help** | | |  |
| **PREV PACKAGE**   **NEXT PACKAGE** | **FRAMES**    **NO FRAMES**     **All Classes** |


---

## Package sbrt.shell.text.fba

Provides classes and interfaces for defining formats for objects relevant to
Flux Balance Analysis.

**See:**
  
          **Description**

| **Interface Summary** | |
| --- | --- |
| **CatalystListFormat** | This interface is used to represent the formats of lists of catalyst names. |
| **CatalystSetFormat** | This interface is used to represent the formats of sets of enzyme names. |
| **ConstraintsFileLineFormat<E>** | This interface is used to represent formats for flux constraints files. |
| **FbaOptFileLineFormat** | This interface is used to represent formats of FBA optimization file lines. |
| **FbaOptHeaderFormatter** | This interface is used to represent formatters for headers used in writing files of FBA optimization data. |
| **FbaOptHeaderParser** | This interface is used to represent parsers for headers used in writing files of FBA optimization data. |
| **FluxCapFormat** | This interface is used to represent the formats of lines of flux cap-containing files. |
| **FluxomeSolutionFileLineFormat** | This interface is used to represent formats for solutions to the linear system of equations formed by a fluxome. |
| **FluxVectorFormat** | This interface is used to represent formats for flux vectors. |
| **RxnNameExprFormat<E extends MathExpr<S>,S>** | This interface is used to represent formats for mathematical expressions of reaction names. |
| **RxnNameListFormat** | This interface is used to represent the formats of collections of reaction names. |
| **RxnNameOrExprFormat** | This interface is used to represent formats for reaction names and mathematical expressions of reaction names. |
| **RxnNameSetFormat** | This interface is used to represent the formats of collections of reaction names. |

| **Class Summary** | |
| --- | --- |
| **BiggBracketParserV1** | This class is used to parse bracketed expressions contained in SBML files from the BiGG Database. |
| **BiggRxnNodeParser** | This class is used to make a direct translation from SBML 'reaction' nodes into Systems Biology Research Tool objects. |
| **CatalystListFormatV1** | This class is a concrete implemenation of `CatalystListFormat`. |
| **CatalystSetFormatV1** | This class is a concrete implemenation of `CatalystSetFormat`. |
| **CatalystVerifier** | This class is used to ensure catalysts exist in a given `CatalyzedFluxome`. |
| **ChemSpeciesVerifier** | This class is used to ensure chemical species exist in a given `Fluxome`. |
| **ConstraintsFileLineFormatV1** | This class is a concrete implemenation of `ConstraintsFileLineFormat`. |
| **ConstraintsFileLineFormatV2** | This class is a concrete implemenation of `ConstraintsFileLineFormat`. |
| **FbaOptFileLineFormatV1** | This class is a concrete implementation of `FbaOptFileLineFormat`. |
| **FbaOptHeaderFormatterV1** | This class is a concrete implemenation of `FbaOptHeaderFormatter`. |
| **FbaOptHeaderParserV1** | This class is a concrete implemenation of `FbaOptHeaderParser`. |
| **FluxCapFormatV1** | This class is a concrete implementation of `FluxCapFormat`. |
| **FluxomeSolFileLineFormatV1** | This class is a concrete implemenation of `FluxomeSolutionFileLineFormat`. |
| **FluxVectorFormatV1** | This class is a concrete implemenation of `FluxVectorFormat`. |
| **IrfFormatV1** | This class is used to format irreversible reaction fluxome files. |
| **IrrevRxnFormatV1** | This class is used to format irreversible reactions for use in flux balance analysis. |
| **PalssonRxnNodeParser** | This class is used to make a direct translation from SBML 'reaction' nodes into Systems Biology Research Tool objects. |
| **RxnNameExprFormatV1** | This class is used to format linear combinations of reaction names. |
| **RxnNameListFormatV1** | This class is a concrete implemenation of `RxnNameListFormat`. |
| **RxnNameOrExprFormatV1** | This class is a concrete implemenation of `RxnNameOrExprFormat`. |
| **RxnNameSetFormatV1** | This class is a concrete implemenation of `RxnNameSetFormat`. |
| **RxnNameVerifier** | This class is used to ensure reaction names exist in a given `Fluxome`. |

## Package sbrt.shell.text.fba Description

Provides classes and interfaces for defining formats for objects relevant to
Flux Balance Analysis.

---


|  |  |  |  |  |  |  |  |  |  |  |
| --- | --- | --- | --- | --- | --- | --- | --- | --- | --- | --- |
| |  |  |  |  |  |  |  |  | | --- | --- | --- | --- | --- | --- | --- | --- | | **Overview** | **Package** | Class | **Use** | **Tree** | **Deprecated** | **Index** | **Help** | | |  |
| **PREV PACKAGE**   **NEXT PACKAGE** | **FRAMES**    **NO FRAMES**     **All Classes** |


---
