# Supplementary material for: The Systems Biology Research Tool: evolvable open-source software
Source: BMC Syst Biol. 2008 Jun 29;2:55. doi: 10.1186/1752-0509-2-55 (PMC2446383; doi:10.1186/1752-0509-2-55)
Supplement: Additional file 1 — SBRT Archive. An archive of the current version of the Systems Biology Research Tool. [file 1752-0509-2-55-S1.zip › sbrt-1.4.0/doc/developers_guide/api/sbrt/shell/text/fba/IrfFormatV1.html]

IrfFormatV1


|  |  |  |  |  |  |  |  |  |  |  |
| --- | --- | --- | --- | --- | --- | --- | --- | --- | --- | --- |
| |  |  |  |  |  |  |  |  | | --- | --- | --- | --- | --- | --- | --- | --- | | **Overview** | **Package** | **Class** | **Use** | **Tree** | **Deprecated** | **Index** | **Help** | | |  |
| **PREV CLASS**   **NEXT CLASS** | **FRAMES**    **NO FRAMES**     **All Classes** |
| SUMMARY: NESTED | FIELD | CONSTR | METHOD | DETAIL: FIELD | CONSTR | METHOD |


---


## sbrt.shell.text.fba Class IrfFormatV1

```
java.lang.Object
  sbrt.shell.text.AbstractMapFormat<java.lang.String,ChemRxn>
      sbrt.shell.text.fba.IrfFormatV1
```

**All Implemented Interfaces:**: Format, MapFormat<java.lang.String,ChemRxn>

---

``` public final class IrfFormatV1 extends AbstractMapFormat<java.lang.String,ChemRxn> ```

This class is used to format irreversible reaction fluxome files.

**Author:**
:   This class was written and documented by
    Jeremiah Wright while in the Wagner lab.

---

| **Constructor Summary** | |
| --- | --- |
| `IrfFormatV1()`             Constructs a new irreversible reaction fluxome format. |


| **Method Summary** | |
| --- | --- |
| `PipeListFormat<ChemRxn>` | `getListFormat()`             Returns the format used for lists of reactions. |
| `EqualsMapEntryFormatV1<java.lang.String,java.lang.String>` | `getMapEntryFormat()`             Returns the format used for reaction name-reaction pairs. |
| `IrrevRxnFormatV1` | `getRxnFormat()`             Returns the format used for reactions. |
| `RxnNameFormatV1` | `getRxnNameFormat()`             Returns the format used for reaction names. |
| `PipeSetFormat<java.lang.String>` | `getSetFormat()`             Returns the format used for sets of reaction names. |

| **Methods inherited from class sbrt.shell.text.AbstractMapFormat** |
| --- |
| `formatKey, formatKeys, formatPair, formatPair, formatValue, formatValues, formatValues, getKeyFormatter, getKeyParser, getValueFormatter, getValueParser, parseAllKeys, parseAllPairs, parseAllValues, parseAllValues, parseKey, parsePair, parseValue` |

| **Methods inherited from class java.lang.Object** |
| --- |
| `clone, equals, finalize, getClass, hashCode, notify, notifyAll, toString, wait, wait, wait` |

| **Constructor Detail** |
| --- |

### IrfFormatV1

```
public IrfFormatV1()
```

:   Constructs a new irreversible reaction fluxome format.


| **Method Detail** |
| --- |

### getRxnNameFormat

```
public RxnNameFormatV1 getRxnNameFormat()
```

:   Returns the format used for reaction names.

    :   **Returns:**: the format used for reaction names.

---


### getRxnFormat

```
public IrrevRxnFormatV1 getRxnFormat()
```

:   Returns the format used for reactions.

    :   **Returns:**: the format used for reactions.

---


### getMapEntryFormat

```
public EqualsMapEntryFormatV1<java.lang.String,java.lang.String> getMapEntryFormat()
```

:   Returns the format used for reaction name-reaction pairs.

    :   **Specified by:**: `getMapEntryFormat` in class `AbstractMapFormat<java.lang.String,ChemRxn>`
    :   **Returns:**: the format used for reaction name-reaction pairs.

---


### getSetFormat

```
public PipeSetFormat<java.lang.String> getSetFormat()
```

:   Returns the format used for sets of reaction names.

    :   **Specified by:**: `getSetFormat` in class `AbstractMapFormat<java.lang.String,ChemRxn>`
    :   **Returns:**: the format used for sets of reaction names.

---


### getListFormat

```
public PipeListFormat<ChemRxn> getListFormat()
```

:   Returns the format used for lists of reactions.

    :   **Specified by:**: `getListFormat` in class `AbstractMapFormat<java.lang.String,ChemRxn>`
    :   **Returns:**: the format used for lists of reactions.


---


|  |  |  |  |  |  |  |  |  |  |  |
| --- | --- | --- | --- | --- | --- | --- | --- | --- | --- | --- |
| |  |  |  |  |  |  |  |  | | --- | --- | --- | --- | --- | --- | --- | --- | | **Overview** | **Package** | **Class** | **Use** | **Tree** | **Deprecated** | **Index** | **Help** | | |  |
| **PREV CLASS**   **NEXT CLASS** | **FRAMES**    **NO FRAMES**     **All Classes** |
| SUMMARY: NESTED | FIELD | CONSTR | METHOD | DETAIL: FIELD | CONSTR | METHOD |


---
