# Supplementary material for: The Systems Biology Research Tool: evolvable open-source software
Source: BMC Syst Biol. 2008 Jun 29;2:55. doi: 10.1186/1752-0509-2-55 (PMC2446383; doi:10.1186/1752-0509-2-55)
Supplement: Additional file 1 — SBRT Archive. An archive of the current version of the Systems Biology Research Tool. [file 1752-0509-2-55-S1.zip › sbrt-1.4.0/doc/developers_guide/api/sbrt/shell/text/fba/package-tree.html]

sbrt.shell.text.fba Class Hierarchy


|  |  |  |  |  |  |  |  |  |  |  |
| --- | --- | --- | --- | --- | --- | --- | --- | --- | --- | --- |
| |  |  |  |  |  |  |  |  | | --- | --- | --- | --- | --- | --- | --- | --- | | **Overview** | **Package** | Class | Use | **Tree** | **Deprecated** | **Index** | **Help** | | |  |
| **PREV**   **NEXT** | **FRAMES**    **NO FRAMES**     **All Classes** |


---


## Hierarchy For Package sbrt.shell.text.fba

**Package Hierarchies:**: All Packages

---

## Class Hierarchy

- java.lang.Object
  - sbrt.shell.text.**AbstractMapFormat**<K,V> (implements sbrt.shell.text.MapFormat<K,V>)
    - sbrt.shell.text.fba.**ConstraintsFileLineFormatV1** (implements sbrt.shell.text.fba.ConstraintsFileLineFormat<E>)- sbrt.shell.text.fba.**ConstraintsFileLineFormatV2** (implements sbrt.shell.text.fba.ConstraintsFileLineFormat<E>)- sbrt.shell.text.fba.**FluxCapFormatV1** (implements sbrt.shell.text.fba.FluxCapFormat)- sbrt.shell.text.fba.**FluxomeSolFileLineFormatV1** (implements sbrt.shell.text.fba.FluxomeSolutionFileLineFormat)- sbrt.shell.text.fba.**FluxVectorFormatV1** (implements sbrt.shell.text.fba.FluxVectorFormat)- sbrt.shell.text.fba.**IrfFormatV1**- sbrt.shell.text.fba.**BiggBracketParserV1**- sbrt.shell.text.fba.**BiggRxnNodeParser**- sbrt.shell.text.fba.**CatalystListFormatV1** (implements sbrt.shell.text.fba.CatalystListFormat)- sbrt.shell.text.fba.**CatalystSetFormatV1** (implements sbrt.shell.text.fba.CatalystSetFormat)- sbrt.shell.text.fba.**CatalystVerifier** (implements sbrt.shell.text.chem.CatalystFormat)- sbrt.shell.text.fba.**ChemSpeciesVerifier** (implements sbrt.shell.text.chem.ChemSpeciesFormat)- sbrt.shell.text.fba.**FbaOptFileLineFormatV1** (implements sbrt.shell.text.fba.FbaOptFileLineFormat)- sbrt.shell.text.fba.**FbaOptHeaderFormatterV1** (implements sbrt.shell.text.fba.FbaOptHeaderFormatter)- sbrt.shell.text.fba.**FbaOptHeaderParserV1** (implements sbrt.shell.text.fba.FbaOptHeaderParser)- sbrt.shell.text.fba.**IrrevRxnFormatV1** (implements sbrt.shell.text.SimpleFormat<T>)- sbrt.shell.text.fba.**PalssonRxnNodeParser**- sbrt.shell.text.fba.**RxnNameExprFormatV1** (implements sbrt.shell.text.fba.RxnNameExprFormat<E,S>)- sbrt.shell.text.fba.**RxnNameListFormatV1** (implements sbrt.shell.text.fba.RxnNameListFormat)- sbrt.shell.text.fba.**RxnNameOrExprFormatV1** (implements sbrt.shell.text.fba.RxnNameOrExprFormat)- sbrt.shell.text.fba.**RxnNameSetFormatV1** (implements sbrt.shell.text.fba.RxnNameSetFormat)- sbrt.shell.text.fba.**RxnNameVerifier** (implements sbrt.shell.text.chem.RxnNameFormat<T>)

## Interface Hierarchy

- sbrt.shell.text.**Format**
  - sbrt.shell.text.**MapFormat**<K,V>
    - sbrt.shell.text.fba.**ConstraintsFileLineFormat**<E>- sbrt.shell.text.fba.**FbaOptFileLineFormat**- sbrt.shell.text.fba.**FluxCapFormat**- sbrt.shell.text.fba.**FluxomeSolutionFileLineFormat**- sbrt.shell.text.fba.**FluxVectorFormat**- sbrt.shell.text.**SimpleFormat**<T> (also extends sbrt.shell.text.Formatter<T>, sbrt.shell.text.Parser<T>)
      - sbrt.shell.text.fba.**CatalystListFormat**- sbrt.shell.text.fba.**CatalystSetFormat**- sbrt.shell.text.fba.**RxnNameExprFormat**<E,S>- sbrt.shell.text.fba.**RxnNameListFormat**- sbrt.shell.text.fba.**RxnNameOrExprFormat**- sbrt.shell.text.fba.**RxnNameSetFormat**- sbrt.shell.text.**Formatter**<T>
    - sbrt.shell.text.fba.**FbaOptHeaderFormatter**- sbrt.shell.text.**SimpleFormat**<T> (also extends sbrt.shell.text.Format, sbrt.shell.text.Parser<T>)
        - sbrt.shell.text.fba.**CatalystListFormat**- sbrt.shell.text.fba.**CatalystSetFormat**- sbrt.shell.text.fba.**RxnNameExprFormat**<E,S>- sbrt.shell.text.fba.**RxnNameListFormat**- sbrt.shell.text.fba.**RxnNameOrExprFormat**- sbrt.shell.text.fba.**RxnNameSetFormat**- sbrt.shell.text.**Parser**<T>
      - sbrt.shell.text.fba.**FbaOptHeaderParser**- sbrt.shell.text.**SimpleFormat**<T> (also extends sbrt.shell.text.Format, sbrt.shell.text.Formatter<T>)
          - sbrt.shell.text.fba.**CatalystListFormat**- sbrt.shell.text.fba.**CatalystSetFormat**- sbrt.shell.text.fba.**RxnNameExprFormat**<E,S>- sbrt.shell.text.fba.**RxnNameListFormat**- sbrt.shell.text.fba.**RxnNameOrExprFormat**- sbrt.shell.text.fba.**RxnNameSetFormat**

---


|  |  |  |  |  |  |  |  |  |  |  |
| --- | --- | --- | --- | --- | --- | --- | --- | --- | --- | --- |
| |  |  |  |  |  |  |  |  | | --- | --- | --- | --- | --- | --- | --- | --- | | **Overview** | **Package** | Class | Use | **Tree** | **Deprecated** | **Index** | **Help** | | |  |
| **PREV**   **NEXT** | **FRAMES**    **NO FRAMES**     **All Classes** |


---
