# Supplementary material for: The Systems Biology Research Tool: evolvable open-source software
Source: BMC Syst Biol. 2008 Jun 29;2:55. doi: 10.1186/1752-0509-2-55 (PMC2446383; doi:10.1186/1752-0509-2-55)
Supplement: Additional file 1 — SBRT Archive. An archive of the current version of the Systems Biology Research Tool. [file 1752-0509-2-55-S1.zip › sbrt-1.4.0/doc/developers_guide/api/sbrt/shell/text/fba/RxnNameSetFormatV1.html]

RxnNameSetFormatV1


|  |  |  |  |  |  |  |  |  |  |  |
| --- | --- | --- | --- | --- | --- | --- | --- | --- | --- | --- |
| |  |  |  |  |  |  |  |  | | --- | --- | --- | --- | --- | --- | --- | --- | | **Overview** | **Package** | **Class** | **Use** | **Tree** | **Deprecated** | **Index** | **Help** | | |  |
| **PREV CLASS**   **NEXT CLASS** | **FRAMES**    **NO FRAMES**     **All Classes** |
| SUMMARY: NESTED | FIELD | CONSTR | METHOD | DETAIL: FIELD | CONSTR | METHOD |


---


## sbrt.shell.text.fba Class RxnNameSetFormatV1

```
java.lang.Object
  sbrt.shell.text.fba.RxnNameSetFormatV1
```

**All Implemented Interfaces:**: RxnNameSetFormat, Format, Formatter<java.util.Set<java.lang.String>>, Parser<java.util.Set<java.lang.String>>, SimpleFormat<java.util.Set<java.lang.String>>

---

``` public final class RxnNameSetFormatV1 extends java.lang.Object implements RxnNameSetFormat ```

This class is a concrete implemenation of `RxnNameSetFormat`.

**Author:**
:   This interface was written and documented by
    Jeremiah Wright while in the Wagner lab.

---

| **Field Summary** | |
| --- | --- |
| `static java.lang.String` | `ALL_RXNS`             The string used to indicate every reaction in a fluxome. |


| **Constructor Summary** | |
| --- | --- |
| `RxnNameSetFormatV1(Fluxome fluxome)`             Constructs a new reaction name collection format using the provided fluxome. |


| **Method Summary** | |
| --- | --- |
| `java.lang.String` | `format(java.util.Set<java.lang.String> reactionNames)`             Returns a formatted string representation of the provided set of reaction names. |
| `Fluxome` | `getFluxome()`             Returns the fluxome used to verify reaction names. |
| `RxnNameVerifier` | `getRxnNameChecker()`             Returns the reaction name verifier. |
| `PipeSetFormat<java.lang.String>` | `getSetFormat()`             Returns the format used for sets of reaction names. |
| `java.util.LinkedHashSet<java.lang.String>` | `parse(java.lang.String set)`             Parses the provided string and returns its corresponding collection of reaction names as a `LinkedHashSet`. |

| **Methods inherited from class java.lang.Object** |
| --- |
| `clone, equals, finalize, getClass, hashCode, notify, notifyAll, toString, wait, wait, wait` |

| **Methods inherited from interface sbrt.shell.text.Formatter** |
| --- |
| `format` |

| **Field Detail** |
| --- |

### ALL\_RXNS

```
public static final java.lang.String ALL_RXNS
```

:   The string used to indicate every reaction in a fluxome.

    **See Also:**: Constant Field Values


| **Constructor Detail** |
| --- |

### RxnNameSetFormatV1

```
public RxnNameSetFormatV1(Fluxome fluxome)
```

:   Constructs a new reaction name collection format
    using the provided fluxome.

    **Parameters:**: `fluxome` - the fluxome used to verify reaction names.


| **Method Detail** |
| --- |

### getFluxome

```
public Fluxome getFluxome()
```

:   Returns the fluxome used to verify reaction names.

    :   **Specified by:**: `getFluxome` in interface `RxnNameSetFormat`
    :   **Returns:**: the fluxome used to verify reaction names.

---


### getSetFormat

```
public PipeSetFormat<java.lang.String> getSetFormat()
```

:   Returns the format used for sets of reaction names.

    :   **Returns:**: the format used for sets of reaction names.

---


### getRxnNameChecker

```
public RxnNameVerifier getRxnNameChecker()
```

:   Returns the reaction name verifier.

    :   **Returns:**: the reaction name verifier.

---


### format

```
public java.lang.String format(java.util.Set<java.lang.String> reactionNames)
```

:   Returns a formatted string representation of the
    provided set of reaction names.

    :   **Specified by:**: `format` in interface `RxnNameSetFormat`
    :   **Parameters:**: `reactionNames` - the set of reaction names to be formatted. **Returns:**: a formatted string representation of the provided set of reaction names.

---


### parse

```
public java.util.LinkedHashSet<java.lang.String> parse(java.lang.String set)
```

:   Parses the provided string and returns its
    corresponding collection of reaction names as a
    `LinkedHashSet`. If the string `ALL_RXNS`
    is present the set will contain the name of
    every reaction in this format's fluxome.

    :   **Specified by:**: `parse` in interface `RxnNameSetFormat` **Specified by:**: `parse` in interface `Parser<java.util.Set<java.lang.String>>`
    :   **Parameters:**: `set` - the string to be parsed. **Returns:**: the set of reaction names corresponding to the provided string.


---


|  |  |  |  |  |  |  |  |  |  |  |
| --- | --- | --- | --- | --- | --- | --- | --- | --- | --- | --- |
| |  |  |  |  |  |  |  |  | | --- | --- | --- | --- | --- | --- | --- | --- | | **Overview** | **Package** | **Class** | **Use** | **Tree** | **Deprecated** | **Index** | **Help** | | |  |
| **PREV CLASS**   **NEXT CLASS** | **FRAMES**    **NO FRAMES**     **All Classes** |
| SUMMARY: NESTED | FIELD | CONSTR | METHOD | DETAIL: FIELD | CONSTR | METHOD |


---
