# Supplementary material for: The Systems Biology Research Tool: evolvable open-source software
Source: BMC Syst Biol. 2008 Jun 29;2:55. doi: 10.1186/1752-0509-2-55 (PMC2446383; doi:10.1186/1752-0509-2-55)
Supplement: Additional file 1 — SBRT Archive. An archive of the current version of the Systems Biology Research Tool. [file 1752-0509-2-55-S1.zip › sbrt-1.4.0/doc/developers_guide/api/sbrt/shell/text/fba/FbaOptHeaderFormatter.html]

FbaOptHeaderFormatter


|  |  |  |  |  |  |  |  |  |  |  |
| --- | --- | --- | --- | --- | --- | --- | --- | --- | --- | --- |
| |  |  |  |  |  |  |  |  | | --- | --- | --- | --- | --- | --- | --- | --- | | **Overview** | **Package** | **Class** | **Use** | **Tree** | **Deprecated** | **Index** | **Help** | | |  |
| **PREV CLASS**   **NEXT CLASS** | **FRAMES**    **NO FRAMES**     **All Classes** |
| SUMMARY: NESTED | FIELD | CONSTR | METHOD | DETAIL: FIELD | CONSTR | METHOD |


---


## sbrt.shell.text.fba Interface FbaOptHeaderFormatter

**All Superinterfaces:**: Formatter<java.lang.String>

**All Known Implementing Classes:**: FbaOptHeaderFormatterV1

---

``` public interface FbaOptHeaderFormatter extends Formatter<java.lang.String> ```

This interface is used to represent formatters for headers
used in writing files of FBA optimization data.

**Author:**
:   This interface was written and documented by
    Jeremiah Wright while in the Wagner lab.

---

| **Method Summary** | |
| --- | --- |
| `Fluxome` | `getFluxome()`             Returns the fluxome used to verify reaction names. |
| `java.lang.String` | `getObjValueHeader()`             Returns the header used to indicate the value of the objective function. |
| `boolean` | `isObjValueHeader(java.lang.String s)`             Indicates if the provided string is the header used to indicate the value of the objective function. |

| **Methods inherited from interface sbrt.shell.text.Formatter** |
| --- |
| `format` |

| **Method Detail** |
| --- |

### getFluxome

```
Fluxome getFluxome()
```

:   Returns the fluxome used to verify reaction names.

    :   **Returns:**: the fluxome used to verify reaction names.

---


### getObjValueHeader

```
java.lang.String getObjValueHeader()
```

:   Returns the header used to indicate the value of the
    objective function.

    :   **Returns:**: the header used to indicate the value of the objective function.

---


### isObjValueHeader

```
boolean isObjValueHeader(java.lang.String s)
```

:   Indicates if the provided string is the header used
    to indicate the value of the objective function.

    :   **Parameters:**: `s` - the string to compare. **Returns:**: `true` if the provided string is equal to the string returned by `getObjValueHeader()`; `false` otherwise.


---


|  |  |  |  |  |  |  |  |  |  |  |
| --- | --- | --- | --- | --- | --- | --- | --- | --- | --- | --- |
| |  |  |  |  |  |  |  |  | | --- | --- | --- | --- | --- | --- | --- | --- | | **Overview** | **Package** | **Class** | **Use** | **Tree** | **Deprecated** | **Index** | **Help** | | |  |
| **PREV CLASS**   **NEXT CLASS** | **FRAMES**    **NO FRAMES**     **All Classes** |
| SUMMARY: NESTED | FIELD | CONSTR | METHOD | DETAIL: FIELD | CONSTR | METHOD |


---
