# Supplementary material for: The Systems Biology Research Tool: evolvable open-source software
Source: BMC Syst Biol. 2008 Jun 29;2:55. doi: 10.1186/1752-0509-2-55 (PMC2446383; doi:10.1186/1752-0509-2-55)
Supplement: Additional file 1 — SBRT Archive. An archive of the current version of the Systems Biology Research Tool. [file 1752-0509-2-55-S1.zip › sbrt-1.4.0/doc/developers_guide/api/sbrt/shell/text/fba/package-use.html]

Uses of Package sbrt.shell.text.fba


|  |  |  |  |  |  |  |  |  |  |  |
| --- | --- | --- | --- | --- | --- | --- | --- | --- | --- | --- |
| |  |  |  |  |  |  |  |  | | --- | --- | --- | --- | --- | --- | --- | --- | | **Overview** | **Package** | Class | **Use** | **Tree** | **Deprecated** | **Index** | **Help** | | |  |
| PREV   NEXT | **FRAMES**    **NO FRAMES**     **All Classes** |


---


## **Uses of Package sbrt.shell.text.fba**

| Packages that use sbrt.shell.text.fba | |
| --- | --- |
| **sbrt.shell.io.fba** | Provides classes and interfaces for reading and writing files relevant to Flux Balance Analysis. |
| **sbrt.shell.mng.fba** | Provides classes and interfaces for creating Flux Balance Analysis process managers. |
| **sbrt.shell.text.fba** | Provides classes and interfaces for defining formats for objects relevant to Flux Balance Analysis. |

| Classes in sbrt.shell.text.fba used by sbrt.shell.io.fba | |
| --- | --- |
| ****FbaOptFileLineFormat****             This interface is used to represent formats of FBA optimization file lines. |
| ****FluxCapFormat****             This interface is used to represent the formats of lines of flux cap-containing files. |
| ****RxnNameExprFormat****             This interface is used to represent formats for mathematical expressions of reaction names. |

| Classes in sbrt.shell.text.fba used by sbrt.shell.mng.fba | |
| --- | --- |
| ****RxnNameExprFormat****             This interface is used to represent formats for mathematical expressions of reaction names. |

| Classes in sbrt.shell.text.fba used by sbrt.shell.text.fba | |
| --- | --- |
| ****CatalystListFormat****             This interface is used to represent the formats of lists of catalyst names. |
| ****CatalystSetFormat****             This interface is used to represent the formats of sets of enzyme names. |
| ****CatalystVerifier****             This class is used to ensure catalysts exist in a given `CatalyzedFluxome`. |
| ****ConstraintsFileLineFormat****             This interface is used to represent formats for flux constraints files. |
| ****FbaOptFileLineFormat****             This interface is used to represent formats of FBA optimization file lines. |
| ****FbaOptHeaderFormatter****             This interface is used to represent formatters for headers used in writing files of FBA optimization data. |
| ****FbaOptHeaderParser****             This interface is used to represent parsers for headers used in writing files of FBA optimization data. |
| ****FluxCapFormat****             This interface is used to represent the formats of lines of flux cap-containing files. |
| ****FluxomeSolutionFileLineFormat****             This interface is used to represent formats for solutions to the linear system of equations formed by a fluxome. |
| ****FluxVectorFormat****             This interface is used to represent formats for flux vectors. |
| ****IrrevRxnFormatV1****             This class is used to format irreversible reactions for use in flux balance analysis. |
| ****RxnNameExprFormat****             This interface is used to represent formats for mathematical expressions of reaction names. |
| ****RxnNameExprFormatV1****             This class is used to format linear combinations of reaction names. |
| ****RxnNameListFormat****             This interface is used to represent the formats of collections of reaction names. |
| ****RxnNameOrExprFormat****             This interface is used to represent formats for reaction names and mathematical expressions of reaction names. |
| ****RxnNameSetFormat****             This interface is used to represent the formats of collections of reaction names. |
| ****RxnNameSetFormatV1****             This class is a concrete implemenation of `RxnNameSetFormat`. |
| ****RxnNameVerifier****             This class is used to ensure reaction names exist in a given `Fluxome`. |

---


|  |  |  |  |  |  |  |  |  |  |  |
| --- | --- | --- | --- | --- | --- | --- | --- | --- | --- | --- |
| |  |  |  |  |  |  |  |  | | --- | --- | --- | --- | --- | --- | --- | --- | | **Overview** | **Package** | Class | **Use** | **Tree** | **Deprecated** | **Index** | **Help** | | |  |
| PREV   NEXT | **FRAMES**    **NO FRAMES**     **All Classes** |


---
