# Supplementary material for: The Systems Biology Research Tool: evolvable open-source software
Source: BMC Syst Biol. 2008 Jun 29;2:55. doi: 10.1186/1752-0509-2-55 (PMC2446383; doi:10.1186/1752-0509-2-55)
Supplement: Additional file 1 — SBRT Archive. An archive of the current version of the Systems Biology Research Tool. [file 1752-0509-2-55-S1.zip › sbrt-1.4.0/doc/developers_guide/api/sbrt/shell/text/fba/RxnNameExprFormatV1.html]

RxnNameExprFormatV1


|  |  |  |  |  |  |  |  |  |  |  |
| --- | --- | --- | --- | --- | --- | --- | --- | --- | --- | --- |
| |  |  |  |  |  |  |  |  | | --- | --- | --- | --- | --- | --- | --- | --- | | **Overview** | **Package** | **Class** | **Use** | **Tree** | **Deprecated** | **Index** | **Help** | | |  |
| **PREV CLASS**   **NEXT CLASS** | **FRAMES**    **NO FRAMES**     **All Classes** |
| SUMMARY: NESTED | FIELD | CONSTR | METHOD | DETAIL: FIELD | CONSTR | METHOD |


---


## sbrt.shell.text.fba Class RxnNameExprFormatV1

```
java.lang.Object
  sbrt.shell.text.fba.RxnNameExprFormatV1
```

**All Implemented Interfaces:**: RxnNameExprFormat<LinearComb<java.lang.String>,java.lang.String>, Format, Formatter<LinearComb<java.lang.String>>, Parser<LinearComb<java.lang.String>>, SimpleFormat<LinearComb<java.lang.String>>

---

``` public final class RxnNameExprFormatV1 extends java.lang.Object implements RxnNameExprFormat<LinearComb<java.lang.String>,java.lang.String> ```

This class is used to format linear combinations of reaction names.

**Author:**
:   This class was written and documented by
    Jeremiah Wright while in the Wagner lab.

---

| **Constructor Summary** | |
| --- | --- |
| `RxnNameExprFormatV1(Fluxome fluxome)`             Constructs a new reaction name expression format using the provided fluxome. |


| **Method Summary** | |
| --- | --- |
| `java.lang.String` | `format(LinearComb<java.lang.String> rxnNameExpr)`             Returns a formatted string representation of the provided linear combination of reaction names. |
| `Fluxome` | `getFluxome()`             Returns the fluxome used to verify reaction names. |
| `LinearCombFormatV1<java.lang.String>` | `getLinerCombFormat()`             Returns the format used for linear combination. |
| `RxnNameVerifier` | `getRxnNameChecker()`             Returns the reaction name verifier. |
| `LinearComb<java.lang.String>` | `parse(java.lang.String rxnNameExpr)`             Parses the provided string and returns its corresponding linear combination of reaction names. |

| **Methods inherited from class java.lang.Object** |
| --- |
| `clone, equals, finalize, getClass, hashCode, notify, notifyAll, toString, wait, wait, wait` |

| **Methods inherited from interface sbrt.shell.text.Formatter** |
| --- |
| `format` |

| **Constructor Detail** |
| --- |

### RxnNameExprFormatV1

```
public RxnNameExprFormatV1(Fluxome fluxome)
```

:   Constructs a new reaction name expression format
    using the provided fluxome.

    **Parameters:**: `fluxome` -


| **Method Detail** |
| --- |

### getFluxome

```
public Fluxome getFluxome()
```

:   Returns the fluxome used to verify reaction names.

    :   **Specified by:**: `getFluxome` in interface `RxnNameExprFormat<LinearComb<java.lang.String>,java.lang.String>`
    :   **Returns:**: the fluxome used to verify reaction names.

---


### getRxnNameChecker

```
public RxnNameVerifier getRxnNameChecker()
```

:   Returns the reaction name verifier.

    :   **Returns:**: the reaction name verifier.

---


### getLinerCombFormat

```
public LinearCombFormatV1<java.lang.String> getLinerCombFormat()
```

:   Returns the format used for linear combination.

    :   **Returns:**: the format used for linear combination.

---


### format

```
public java.lang.String format(LinearComb<java.lang.String> rxnNameExpr)
```

:   Returns a formatted string representation of the
    provided linear combination of reaction names.

    :   **Parameters:**: `rxnNameExpr` - the linear combination to be formatted. **Returns:**: a formatted string representation of the provided linear combination of reaction names.

---


### parse

```
public LinearComb<java.lang.String> parse(java.lang.String rxnNameExpr)
```

:   Parses the provided string and returns its
    corresponding linear combination of reaction names.

    :   **Specified by:**: `parse` in interface `Parser<LinearComb<java.lang.String>>`
    :   **Parameters:**: `rxnNameExpr` - the string to be parsed. **Returns:**: the linear combination of reaction names corresponding to the provided string.


---


|  |  |  |  |  |  |  |  |  |  |  |
| --- | --- | --- | --- | --- | --- | --- | --- | --- | --- | --- |
| |  |  |  |  |  |  |  |  | | --- | --- | --- | --- | --- | --- | --- | --- | | **Overview** | **Package** | **Class** | **Use** | **Tree** | **Deprecated** | **Index** | **Help** | | |  |
| **PREV CLASS**   **NEXT CLASS** | **FRAMES**    **NO FRAMES**     **All Classes** |
| SUMMARY: NESTED | FIELD | CONSTR | METHOD | DETAIL: FIELD | CONSTR | METHOD |


---
