# Supplementary material for: The Systems Biology Research Tool: evolvable open-source software
Source: BMC Syst Biol. 2008 Jun 29;2:55. doi: 10.1186/1752-0509-2-55 (PMC2446383; doi:10.1186/1752-0509-2-55)
Supplement: Additional file 1 — SBRT Archive. An archive of the current version of the Systems Biology Research Tool. [file 1752-0509-2-55-S1.zip › sbrt-1.4.0/doc/developers_guide/api/sbrt/shell/text/fba/RxnNameListFormat.html]

RxnNameListFormat


|  |  |  |  |  |  |  |  |  |  |  |
| --- | --- | --- | --- | --- | --- | --- | --- | --- | --- | --- |
| |  |  |  |  |  |  |  |  | | --- | --- | --- | --- | --- | --- | --- | --- | | **Overview** | **Package** | **Class** | **Use** | **Tree** | **Deprecated** | **Index** | **Help** | | |  |
| **PREV CLASS**   **NEXT CLASS** | **FRAMES**    **NO FRAMES**     **All Classes** |
| SUMMARY: NESTED | FIELD | CONSTR | METHOD | DETAIL: FIELD | CONSTR | METHOD |


---


## sbrt.shell.text.fba Interface RxnNameListFormat

**All Superinterfaces:**: Format, Formatter<java.util.List<java.lang.String>>, Parser<java.util.List<java.lang.String>>, SimpleFormat<java.util.List<java.lang.String>>

**All Known Implementing Classes:**: RxnNameListFormatV1

---

``` public interface RxnNameListFormat extends SimpleFormat<java.util.List<java.lang.String>> ```

This interface is used to represent the formats of collections
of reaction names.

**Author:**
:   This interface was written and documented by
    Jeremiah Wright while in the Wagner lab.

---

| **Method Summary** | |
| --- | --- |
| `java.lang.String` | `format(java.util.List<java.lang.String> reactionNames)`             Returns a formatted string representation of the provided list of reaction names. |
| `Fluxome` | `getFluxome()`             Returns the fluxome used to verify reaction names. |
| `java.util.List<java.lang.String>` | `parse(java.lang.String reactionNames)`             Parses the provided string and returns the list of reaction names it contains. |

| **Methods inherited from interface sbrt.shell.text.Formatter** |
| --- |
| `format` |

| **Method Detail** |
| --- |

### getFluxome

```
Fluxome getFluxome()
```

:   Returns the fluxome used to verify reaction names.

    :   **Returns:**: the fluxome used to verify reaction names.

---


### format

```
java.lang.String format(java.util.List<java.lang.String> reactionNames)
```

:   Returns a formatted string representation of the
    provided list of reaction names.

    :   **Parameters:**: `reactionNames` - the list of reaction names to be formatted. **Returns:**: a formatted string representation of the provided list of reaction names.

---


### parse

```
java.util.List<java.lang.String> parse(java.lang.String reactionNames)
```

:   Parses the provided string and returns the
    list of reaction names it contains.

    :   **Specified by:**: `parse` in interface `Parser<java.util.List<java.lang.String>>`
    :   **Parameters:**: `reactionNames` - the string to be parsed. **Returns:**: the list of reaction names contained in the provided string.


---


|  |  |  |  |  |  |  |  |  |  |  |
| --- | --- | --- | --- | --- | --- | --- | --- | --- | --- | --- |
| |  |  |  |  |  |  |  |  | | --- | --- | --- | --- | --- | --- | --- | --- | | **Overview** | **Package** | **Class** | **Use** | **Tree** | **Deprecated** | **Index** | **Help** | | |  |
| **PREV CLASS**   **NEXT CLASS** | **FRAMES**    **NO FRAMES**     **All Classes** |
| SUMMARY: NESTED | FIELD | CONSTR | METHOD | DETAIL: FIELD | CONSTR | METHOD |


---
