# Supplementary material for: The Systems Biology Research Tool: evolvable open-source software
Source: BMC Syst Biol. 2008 Jun 29;2:55. doi: 10.1186/1752-0509-2-55 (PMC2446383; doi:10.1186/1752-0509-2-55)
Supplement: Additional file 1 — SBRT Archive. An archive of the current version of the Systems Biology Research Tool. [file 1752-0509-2-55-S1.zip › sbrt-1.4.0/doc/developers_guide/api/sbrt/shell/text/fba/RxnNameOrExprFormatV1.html]

RxnNameOrExprFormatV1


|  |  |  |  |  |  |  |  |  |  |  |
| --- | --- | --- | --- | --- | --- | --- | --- | --- | --- | --- |
| |  |  |  |  |  |  |  |  | | --- | --- | --- | --- | --- | --- | --- | --- | | **Overview** | **Package** | **Class** | **Use** | **Tree** | **Deprecated** | **Index** | **Help** | | |  |
| **PREV CLASS**   **NEXT CLASS** | **FRAMES**    **NO FRAMES**     **All Classes** |
| SUMMARY: NESTED | FIELD | CONSTR | METHOD | DETAIL: FIELD | CONSTR | METHOD |


---


## sbrt.shell.text.fba Class RxnNameOrExprFormatV1

```
java.lang.Object
  sbrt.shell.text.fba.RxnNameOrExprFormatV1
```

**All Implemented Interfaces:**: RxnNameOrExprFormat, Format, Formatter<java.lang.Object>, Parser<java.lang.Object>, SimpleFormat<java.lang.Object>

---

``` public final class RxnNameOrExprFormatV1 extends java.lang.Object implements RxnNameOrExprFormat ```

This class is a concrete implemenation of `RxnNameOrExprFormat`.

**Author:**
:   This class was written and documented by
    Jeremiah Wright while in the Wagner lab.

---

| **Constructor Summary** | |
| --- | --- |
| `RxnNameOrExprFormatV1(Fluxome fluxome)`             Constructs a new reaction name and expression format. |


| **Method Summary** | |
| --- | --- |
| `java.lang.String` | `format(java.lang.Object rxnNameOrExpression)`             Returns the string representation of the provided object. |
| `Fluxome` | `getFluxome()`             Returns the fluxome used to verify reaction names. |
| `RxnNameExprFormatV1` | `getLinearCombFormat()`             Returns the reaction name expression format. |
| `RxnNameVerifier` | `getRxnNameChecker()`             Returns the reaction name verifier. |
| `java.lang.Object` | `parse(java.lang.String rxnNameOrExpression)`             Parses the provided string and returns its corresponding object. |

| **Methods inherited from class java.lang.Object** |
| --- |
| `clone, equals, finalize, getClass, hashCode, notify, notifyAll, toString, wait, wait, wait` |

| **Constructor Detail** |
| --- |

### RxnNameOrExprFormatV1

```
public RxnNameOrExprFormatV1(Fluxome fluxome)
```

:   Constructs a new reaction name and expression format.

    **Parameters:**: `fluxome` - the fluxome used to verify reaction names.


| **Method Detail** |
| --- |

### getFluxome

```
public Fluxome getFluxome()
```

:   Returns the fluxome used to verify reaction names.

    :   **Specified by:**: `getFluxome` in interface `RxnNameOrExprFormat`
    :   **Returns:**: the fluxome used to verify reaction names.

---


### getRxnNameChecker

```
public RxnNameVerifier getRxnNameChecker()
```

:   Returns the reaction name verifier.

    :   **Returns:**: the reaction name verifier.

---


### getLinearCombFormat

```
public RxnNameExprFormatV1 getLinearCombFormat()
```

:   Returns the reaction name expression format.

    :   **Returns:**: the reaction name expression format.

---


### format

```
public java.lang.String format(java.lang.Object rxnNameOrExpression)
```

:   Returns the string representation of the provided
    object.

    :   **Specified by:**: `format` in interface `Formatter<java.lang.Object>`
    :   **Parameters:**: `rxnNameOrExpression` - a reaction name or `LinearComb` of reaction names. **Returns:**: the string representation of the provided object.

---


### parse

```
public java.lang.Object parse(java.lang.String rxnNameOrExpression)
```

:   Parses the provided string and returns its corresponding
    object.

    :   **Specified by:**: `parse` in interface `Parser<java.lang.Object>`
    :   **Parameters:**: `rxnNameOrExpression` - a string representation of a reaction name or expression. **Returns:**: the reaction name or expression contained in the provided string.


---


|  |  |  |  |  |  |  |  |  |  |  |
| --- | --- | --- | --- | --- | --- | --- | --- | --- | --- | --- |
| |  |  |  |  |  |  |  |  | | --- | --- | --- | --- | --- | --- | --- | --- | | **Overview** | **Package** | **Class** | **Use** | **Tree** | **Deprecated** | **Index** | **Help** | | |  |
| **PREV CLASS**   **NEXT CLASS** | **FRAMES**    **NO FRAMES**     **All Classes** |
| SUMMARY: NESTED | FIELD | CONSTR | METHOD | DETAIL: FIELD | CONSTR | METHOD |


---
