# Supplementary material for: The Systems Biology Research Tool: evolvable open-source software
Source: BMC Syst Biol. 2008 Jun 29;2:55. doi: 10.1186/1752-0509-2-55 (PMC2446383; doi:10.1186/1752-0509-2-55)
Supplement: Additional file 1 — SBRT Archive. An archive of the current version of the Systems Biology Research Tool. [file 1752-0509-2-55-S1.zip › sbrt-1.4.0/doc/developers_guide/api/sbrt/shell/text/fba/class-use/CatalystVerifier.html]

Uses of Class sbrt.shell.text.fba.CatalystVerifier


|  |  |  |  |  |  |  |  |  |  |  |
| --- | --- | --- | --- | --- | --- | --- | --- | --- | --- | --- |
| |  |  |  |  |  |  |  |  | | --- | --- | --- | --- | --- | --- | --- | --- | | **Overview** | **Package** | **Class** | **Use** | **Tree** | **Deprecated** | **Index** | **Help** | | |  |
| PREV   NEXT | **FRAMES**    **NO FRAMES**     **All Classes** |


---


## **Uses of Class sbrt.shell.text.fba.CatalystVerifier**

| Packages that use CatalystVerifier | |
| --- | --- |
| **sbrt.shell.text.fba** | Provides classes and interfaces for defining formats for objects relevant to Flux Balance Analysis. |

| Uses of CatalystVerifier in sbrt.shell.text.fba | |
| --- | --- |

| Methods in sbrt.shell.text.fba that return CatalystVerifier | |
| --- | --- |
| `CatalystVerifier` | `CatalystListFormatV1.getEnzymeChecker()`             Returns the catalyst name verifier. |
| `CatalystVerifier` | `CatalystSetFormatV1.getEnzymeChecker()`             Returns the catalyst name verifier. |

---


|  |  |  |  |  |  |  |  |  |  |  |
| --- | --- | --- | --- | --- | --- | --- | --- | --- | --- | --- |
| |  |  |  |  |  |  |  |  | | --- | --- | --- | --- | --- | --- | --- | --- | | **Overview** | **Package** | **Class** | **Use** | **Tree** | **Deprecated** | **Index** | **Help** | | |  |
| PREV   NEXT | **FRAMES**    **NO FRAMES**     **All Classes** |


---
