# Supplementary material for: The Systems Biology Research Tool: evolvable open-source software
Source: BMC Syst Biol. 2008 Jun 29;2:55. doi: 10.1186/1752-0509-2-55 (PMC2446383; doi:10.1186/1752-0509-2-55)
Supplement: Additional file 2 — SBRT Processes. Descriptions of the 51 processes currently implemented in the Systems Biology Research Tool. [file 1752-0509-2-55-S2.pdf]

Descriptions of the 51 processes currently implemented in the Systems Biology Research Tool.

Category	Process Name	Brief Description
Flux Optimization	FBA Optimization	Used to compute the optimal value of a flux or linear combination of fluxes in a stoichiometric network.
	Reaction Deletion	Used to compute the effect of deleting sets of reactions in a stoichiometric network.
	Catalyst Deletion	Used to compute the effect of deleting sets of catalysts in a stoichiometric network.
	Objective Function Analysis	Used to compute the optimal values of multiple objective functions for a stoichiometric network.
	Constraint Variation	Used to compute the optimal values of a single objective function for multiple sets of flux constraints.
	Constraint Variation-Reaction Deletion	Used to compute the combined effects of deleting reactions and varying the flux constraints in a stoichiometric network.
	Constraint Variation-Catalyst Deletion	Used to compute the combined effects of deleting catalysts and varying the flux constraints in a stoichiometric network.
	Constraint Variation-Objective Function Analysis	Used to compute the optimal values of multiple objective functions for multiple sets of flux constraints.
Flux Variability	Simple Flux Intervals	Used to compute the intervals of fluxes in a stoichiometric network in the simplest possible way.
	Constrained Reverse Reaction Flux Intervals	Used to compute the intervals of fluxes in a stoichiometric network after constraining the fluxes of reversible reactions.
	Flux Cap Identification	Used to create *caps* for each unbounded flux in a stoichiometric network.
	Mahadevan-Schilling Flux Intervals	Used to compute the Mahadevan-Schilling flux intervals in a stoichiometric network.
	Constraint Variation-Simple Flux Intervals	Used to compute the *simple* flux intervals for multiple sets of flux constraints.
	Constraint Variation-Constrained Reverse Reaction Flux Intervals	Used to compute *constrained reverse reaction* flux intervals for multiple sets of flux constraints.
	Constraint Variation-Mahadevan-Schilling Flux Intervals	Used to compute Mahadevan-Schilling flux intervals for multiple sets of flux constraints.

**Chemical Reaction Pathway Identification**	Extreme Current Identification	Used to identify the extreme currents in stoichiometric networks.
	WW Network Reduction	Used to reduce the size of stoichiometric networks for the purpose of identifying the cycles they contain.
	MS Network Reduction	Used to reduce the size of stoichiometric networks for the purpose of identifying the cycles they contain.
	SLP Cycle Identification	Used to identify the cycles in stoichiometric networks.
**Flux Space Sampling**	Random Constraint Generator	Used to generate random flux constraints.
	Random Objective Function Generator	Used to generate random objective functions.
	Initial Point Generator	Used to compute an initial flux vector for use in CD Hit-and-Run Analysis.
	Coordinate Direction Hit-and-Run Analysis	Used to compute random, uniformly-distributed flux vectors from the interior flux space.
	Space Variation-Initial Point Generator	Used to compute initial flux vectors for use in Space Variation-CD Hit-and-Run Analysis.
	Space Variation-Coordinate Direction Hit-and-Run Analysis	Used to compute random, uniformly-distributed flux vectors from the interiors of multiple flux spaces.
**Flux Data Analysis**	Flux Activity Analysis	Used to analyze the activity of fluxes in a collection of flux vectors.
	Flux Plasticity Analysis	Used to analyze the plasticity of fluxes in a collection of flux interval vectors.
**Stoichiometric Network Utilities**	Simple Reaction File Reader	Used to translate files containing a list of chemical reactions into human-readable *FBA Reaction Files* .
	Palsson-SBML File Reader	Used to read SBML files from Dr. Palsson's website.
	BiGG-SBML File Reader	Used to read SBML files from the BiGG Database.
	Palsson-SBML File Translation	Used to translate SBML files from Dr. Palsson's website into human-readable *FBA Reaction Files* and *Reaction-Catalyst Association Files* .
	BiGG-SBML File Translation	Used to translate SBML files from the BiGG Database into human-readable *FBA Reaction Files* and *Reaction-Catalyst Association Files* .
	Metatool File Writer	Used to convert *FBA Reaction Files* into input files for Metatool.
	Network Information Gatherer	Used to gather basic information about a stoichiometric network.
	FBA System Solver	Used to solve the equation $Sv = 0$.

**Graph Theory**	Path Identification in a Directed Graph	Used to identify the simple paths in a directed graph.
	Cycle Identification in a Directed Graph	Used to identify the simple cycles in a directed graph.
**Geometry**	Coordinate Directions Hit-and-Run	Used to generate random interior points within convex polytopes.
**Algebra**	Linear System Solver	Used to solve systems of linear equations using Mathematica.
	Multiple-Vectors File Conversion	Used to convert a single multiple-vectors file into multiple single-vector files.
	Single-Vector Files Conversion	Used to convert multiple single-vector files into a single multiple-vectors file.
	Matrix File Conversion	Used to convert a matrix into a list of linear combinations.
	Linear Combination File Combination	Used to convert a list of linear combinations into a matrix.
**Combinatorics**	Single-Element Unions	Used to compute single-element unions of collections of sets.
	Strict Single-Element Unions	Used to compute strict single-element unions of collections of sets.
**Statistics**	Correlation Estimation	Used to compute a variety of correlation coefficients using *R*.
	Kendall's Tau Correlation	Used to compute Kendall's tau correlation statistics.
	Mann-Whitney U Test	Used to compute Mann-Whitney U statistics.
**General Utilities**	Interval Comparison	Used to compare intervals for equality within a given tolerance.
	Numerical Vector Comparison	Used to compare numerical vectors for equality within a given tolerance.
	Variable Participation	Used to group mathematical expressions based on the variables they contain.
