# Supplementary material for: The Systems Biology Research Tool: evolvable open-source software
Source: BMC Syst Biol. 2008 Jun 29;2:55. doi: 10.1186/1752-0509-2-55 (PMC2446383; doi:10.1186/1752-0509-2-55)
Supplement: Additional file 3 — Performance Comparisons. A description of performance comparisons between the Systems Biology Research Tool and the COBRA Toolbox. [file 1752-0509-2-55-S3.pdf]

Supplementary material

Performance comparisons. Of all existing packages, the COBRA Toolbox is most similar to the SBRT in terms of the computational procedures offered by both. Because of these similarities, we performed a comparative performance analysis of some capabilities offered by both packages. Specifically, we carried out 5 analyses using the *in silico* model of *Saccharomyces cerevisiae* metabolism iND750 [1]. In analyses A and B, the model was provided a minimal growth-supporting medium with glucose as the sole carbon and energy source. This was achieved by setting the maximum glucose supply rate to an arbitrary value of 1, constraining the supply rates of oxygen, ammonium, sulfate, phosphate, and water to be unbounded in the positive direction, and setting the supply rates of all other extracellular metabolites to zero. In analyses A and B, the variability of all fluxes in the network and the effect of all single-gene deletions on the maximum biomass production rate were determined, respectively.

In analyses C, D, and E, the iND750 model was sequentially provided 100 randomly generated media. Each of these media was created beforehand by setting the maximum supply rate of 58 (one half of the total) randomly chosen extracellular metabolites to the value 10 and setting the remaining supply rates to zero and by ensuring each medium supported biomass production. Identical sets of media were used in analyses C, D, and E and by both the Systems Biology Research Tool and the COBRAToolbox. In analyses C, D, and E, the maximum biomass production rate, the variability of all fluxes, and the effect of all single-gene deletions on the maximum biomass production rate were computed, respectively.

Software versions. The Systems Biology Research Tool 1.1.0 and the COBRAToolbox 1.3.3 were used for all performance comparisons. The SBRT ran within Sun's Java HotSpot(TM) 64-Bit Server VM (build 1.6.0_03-b05, mixed mode) and used GLPK 4.8 to solve all linear programs and Xerces-J 2.1.0 to parse SBML files. The COBRAToolbox ran within MATLAB 7.2.0 and used GLPK 4.23 to solve all linear programs and libsbml 2.3.4 to parse SBML files. GLPKMEX 2.4 and the SBMLToolbox 2.0.2 were used by the COBRAToolbox to enable communication with GLPK and libsbml, respectively.

Program execution. All performance comparisons were made on a Dell Precision 490 computer equipped with a 2.33 GHz Intel Xeon processor with Kubuntu 7.10 (AMD64) as the operating system. A bash script was used to execute 10 programs sequentially for each analysis for each software package. The time was recorded before each program began and after each program finished execution to determine the total running time. A perl script (`memmon`) was used to frequently sample the contents of `/proc/meminfo` to monitor the memory usage during each program execution. Memory monitoring began before each program was executed to establish a baseline, and the maximum memory consumption during program execution was measured relative to this baseline.

Source code and data. The scripts, MATLAB m files, and input files used to perform these analyses, and the data generated, are all contained in the file http://www.bioc.uzh.ch/wagner/software/SBRT/suppl_material.zip.

References

1. Duarte NC, Herrgard MJ, Palsson BO: **Reconstruction and Validation of Saccharomyces cerevisiae iND750, a Fully Compartmentalized Genome-Scale Metabolic Model.** *Genome Res* 2004, **14:**1298-1309.
